# Protein intake in cancer: Does it improve nutritional status and/or modify tumour response to chemotherapy?

**DOI:** 10.1002/jcsm.13276

**Published:** 2023-09-04

**Authors:** Martin Boutière, Cécile Cottet‐Rousselle, Céline Coppard, Karine Couturier, Catherine Féart, Morgane Couchet, Christelle Corne, Christophe Moinard, Charlotte Breuillard

**Affiliations:** ^1^ Université Grenoble Alpes, Laboratory of Fundamental and Applied Bioenergetics (LBFA) Grenoble France; ^2^ Université Grenoble Alpes, INSERM, CNRS, Institute for Advanced Biosciences (IAB) Grenoble France; ^3^ Université de Bordeaux, INSERM, BPH, U1219 Bordeaux France; ^4^ Université Grenoble Alpes, Grenoble Alpes University Hospital, Institute of Biology, Laboratory of Metabolic Diseases Grenoble France

**Keywords:** Cancer, Chemotherapy, Protein diet, Tumour, Cachexia, Malnutrition

## Abstract

**Background:**

Combating malnutrition and cachexia is a core challenge in oncology. To limit muscle mass loss, the use of proteins in cancer is encouraged by experts in the field, but it is still debated due to their antagonist effects. Indeed, a high protein intake could preserve lean body mass but may promote tumour growth, whereas a low‐protein diet could reduce tumour size but without addressing cachexia. Here we used a realistic rodent model of cancer and chemotherapy to evaluate the influence of different protein intakes on cachexia, tumour response to chemotherapy and immune system response. The goal is to gain a closer understanding of the effect of protein intake in cancer patients undergoing chemotherapy.

**Methods:**

Female Fischer 344 rats were divided into six groups: five groups (*n* = 14 per group) with cancer (Ward colon tumour) and chemotherapy were fed with isocaloric diets with 8%, 12%, 16%, 24% or 32% of caloric intake from protein and one healthy control group (*n* = 8) fed a 16% protein diet, considered as a standard diet. Chemotherapy included two cycles, 1 week apart, each consisting of an injection of CPT‐11 (50 mg/kg) followed by 5‐fluorouracil (50 mg/kg) the day after. Food intake, body weight, and tumour size were measured daily. On day 9, the rats were euthanized and organs were weighed. Body composition was determined and protein content and protein synthesis (SUnSET method) were measured in the muscle, liver, intestine, and tumour. Immune function was explored by flow cytometry.

**Results:**

Cancer and chemotherapy led to a decrease in body weight characterized by a decrease of both fat mass (−56 ± 3%, *P* < 0.05) and fat‐free mass (−8 ± 1%, *P* < 0.05). Surprisingly, there was no effect of protein diet on body composition, muscle or tumour parameters (weight, protein content, or protein synthesis) but a high cumulative protein intake was positively associated with a high relative body weight and high fat‐free mass. The immune system was impacted by cancer and chemotherapy but not by the different amount of protein intake.

**Conclusions:**

Using a realistic model of cancer and chemotherapy, we demonstrated for the first time that protein intake did not positively or negatively modulate tumour growth. Moreover, our results suggested that a high cumulative protein intake was able to improve moderately nutritional status in chemotherapy treated cancer rodents. Although this work cannot be evaluated clinically for ethical reasons, it nevertheless brings an essential contribution to nutrition management for cancer patients.

## Introduction

The prevalence of malnutrition among patients with cancer is about 20% and can reach more than 70% depending on patient age, cancer type and cancer stage. Malnutrition in cancer leads to weight loss and then decrease the tolerance to chemotherapy, forcing oncologists to reduce chemotherapy dose and thus chemotherapy efficacy.^1^, ^2^, ^3^, ^4^, ^5^, ^6^, ^7^ It is a stark fact that 10–20% of mortality in cancer patients is due to malnutrition rather than malignancy itself.^1^, ^4^ Combating malnutrition and cachexia is therefore a core challenge in oncology, as underlined by experts from the ESPEN (European Society for Clinical Nutrition and Metabolism) and the ESMO (European Society for Medical Oncology).^1^, ^2^, ^3^

Both ESPEN and ESMO recommend implementing an appropriate nutritional support in cancer. However, combining efficient cancer treatments while maintaining a satisfying nutritional status is a complex challenge: the adopted nutritional strategies do not interfere with the treatment, but do feed the patient without feeding the tumour (even though the ESPEN expert group rules out this possibility).^2^, ^5^ In a recent position paper, Bozzetti and Stanga analysed 12 publications evaluating the effect of nutritional support on tumour cell proliferation.^8^ They reported many clinical or methodological concerns which prevented drawn definite conclusion. In the current clinical practice, many clinicians are reluctant to risk of supplementing patients for fear of nourishing the tumour, as evoked by Bozzetti and Stanga and by Ford *et al*.^8^, ^9^

Regarding the protein intake, the picture complex further. Most of experts agreed that relevant data is scarce and that further studies are required to define the appropriate protein intake. However, the same experts recommend increasing protein intake from 0.8 g/kg/day for healthy people to 1.2–1.5 g/kg/day in cancer patients.^1^, ^2^, ^3^, ^5^, ^10^ From a mechanistic point of view, there is a strong rationale to increase the protein intake of patients. Indeed, using proteins which activate *mTORC1* (mammalian target of rapamycin complex 1) will increase muscle protein synthesis (MPS) and thus the patient muscle mass.^11^, ^12^, ^13^ Furthermore, a high‐protein rich diet could activate the immune system to help fight against tumour cells.^14^ While this approach could be beneficial for the patient, it could also be beneficial for the tumour: if increased dietary protein intakes induce an increased protein synthesis in the tumour, *via* the same mTOR signalling, it could thereby promote tumour growth. Besides, mTOR inhibitors are used as anti‐cancer drugs.^15^, ^16^, ^17^

Conversely, a low‐protein diet would limit tumour growth by increasing tumour immunosurveillance.^18^, ^19^ However, adopting this dietary strategy could have harmful consequences for patients, by worsening their undernutrition and cachexia status. Investigation is therefore needed to unravel this complex issue.

To date, to the best of our knowledge, the impact of the amount of protein ingested in both muscle wasting and tumour growth has never been evaluated in a realistic research model involving cancer and chemotherapy. To addresses this gap, we evaluated the influence of different protein diets on muscle wasting, tumour growth and immune system response in a tumour‐bearing rat model undergoing chemotherapy. The objective of this work was to gain a firm understanding of the effect of protein intake in oncology and determine the optimal dose of protein that would reduce cancer cachexia to minimum while minimizing tumour growth. This work has important implications for improving the management and effectiveness of treatment for cancer.

## Methods

### Animals

All the procedures were registered as compliant with European Directives on animal care and use for research purposes (APAFIS#11492‐2017112715178992), approved by the institutional animal care and use committee affiliated to the animal facility of the Grenoble Alpes University (B3842110001), and endorsed by the French Ministry of Research (9998_LBFA‐U1055).

Seventy‐eight female Fischer 344 rats (10–12 weeks old, 130–160 g) were housed for the 8‐day acclimatization period at six per cage in a temperature‐controlled facility (22 ± 2°C) on a 12 h light/dark cycle. Water and food were available *ad libitum*.

### Experimental design

Relative body weight, relative food intake and relative tumour volume of each animal were compared against the baseline (day 0 value).

#### Choice of the animal model

This model, developed by Cao *et al*.^20^ and characterized by V. E. Baracos's team,^21^, ^22^ is a realistic preclinical model involving tumour and treatment (i.e chemotherapy). Interestingly, it is closer to malnutrition observed in cancer patients (i.e. body weight loss >5% in 6 months)^23^ compared with majority of murine models.^24^, ^25^ This model, mimicking early cancer consequences, appears more realistic than others which mimic advanced clinical situations.

#### Tumour

**Figure 1:**
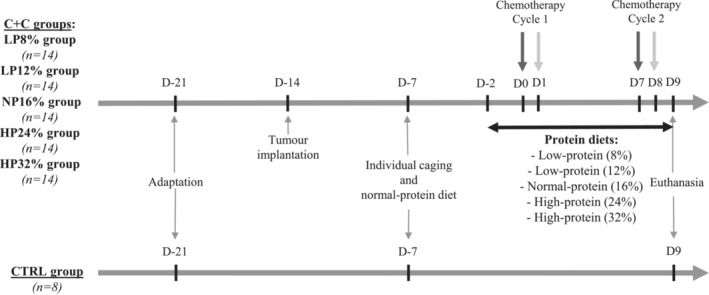
Experimental design. After a 1‐week acclimatization period (D‐14), 70 11‐week‐old female fisher rats received tumour injection (C + C rats). One week later (D‐7), all the animals (*n* = 78) received the normal‐protein diet. Then, after 13 days of tumour growth (D‐2), the rats received either the normal‐protein diet (NP16%, *n* = 14) or a low‐protein diet with 8% protein (LP8%, *n* = 14), a low‐protein diet with 12% protein (LP12%, *n* = 14), a high‐protein diet with 24% protein (HP24%, *n* = 14) or a high‐protein diet with 32% protein (HP32%, *n* = 14). Two days later, the first chemotherapy cycle was initiated: irinotecan (CPT‐11; 50 mg/kg; dark grey arrow) at D0 and 5‐fluorouracil (5‐FU; 50 mg/kg; light grey arrow) at D1. One week later, the rats received the second cycle of chemotherapy (CPT‐11 at D7 and 5‐FU at D8). The rats were sacrificed at D9 for analysis.

After an 8‐day acclimatization period (*Figure* 1), 70 ‘cancer + chemotherapy’ rats (‘C + C’ rats) were injected with Ward colon tumour. We thank Pr VE Baracos (Cross Cancer Institute, University of Alberta) for the gift of the Ward colorectal carcinoma.^21^, ^22^ The other rats constituted the healthy control group without tumour or chemotherapy (‘Ctrl’, *n* = 8). Pieces of non‐necrotic tumour (0.1 g) were subcutaneously transplanted onto the flank of the animals to serve for tumour growth monitoring.^20^, ^21^, ^22^ As tumour growth is heterogeneous, each C + C rat group was composed of 14 rats. Tumour volume was measured every day in three dimensions with a calliper to determine length (L, cm), width (W, cm) and height (H, cm). Tumour volume was then calculated using the following formula: 0.5 × L × W × H cm^3^.

#### Diets

One week after tumour injection (*Figure* 1), tumour‐bearing and healthy control rats were housed in individual cages in order to perform food intake measurement. All rats received a standard semi‐purified diet (AIN93G: 15.9% protein, 7.4% lipids, 61.7% carbohydrates; SAFE, Augy, France), called ‘normal‐protein diet’, *ad libitum* (*Table* 1). Body weight and food intake were monitored daily.

**Table 1:** Composition of the diets

	LP8%	LP12%	NP16% normal‐protein diet	HP24%	HP32%
Pregelatinized cornstarch	491.0	444.3	397.5	301.9	207.4
Casein	88	134	180	274	367
Maltodextrin	142	142	142	142	142
Sucrose	110	110	110	110	110
Soya oil	70	70	70	70	70
Crude cellulose	50	50	50	50	50
Minerals PM AIN 93 M/G 3.5%	35	35	35	35	35
Vitamins PV AIN 93 M/G 1%	10	10	10	10	10
L‐cystine	1.5	2.2	3.0	4.6	6.1
Bitartrate choline	2.5	2.5	2.5	2.5	2.5
Protein %	8.0	12.0	15.9	24.0	32.0
Fat %	7.3	7.3	7.4	7.5	7.6
Minerals %	2.5	2.6	2.7	2.8	3.0
Cellulose %	3.6	3.6	3.6	3.6	3.5
Starch %	56.3	52.1	47.9	39.3	30.8
Sugars %	13.8	13.8	13.8	13.8	13.9
ENA %	71.1	67.1	63.1	55.0	46.9
ATWATER (kcal/kg)	3821.4	3824.8	3828.4	3835.5	3842.6
ATWATER (MJ/kg)	16.0	16.0	16.0	16.1	16.1
Nitrogen‐calorie ratio (kcal/g)	274	174	125	75	50

One week after tumour injection, rats received the standard normal‐protein diet, which is a nutritionally complete semi‐synthetic diet (AIN93G ‐ 15.9% protein, 7.4% lipids, 61.7% carbohydrates; SAFE, Augy, France; NP16%). Two days before the start of chemotherapy, C + C rats were divided into five groups to receive one of five diets (i.e. LP8%, LP12%, NP16%, HP24% or HP32%). Control rats received the NP16% diet only.

After 12 days of tumour growth, when they had reached approximatively 1 cm^3^, the C + C rats were divided into five groups according to body weight and tumour size, to ensure homogeneity in the five groups at that time of the study. Each group then received one of the five iso‐caloric diets stratified into five different protein (casein) contents (*Table* 1). The Ctrl group and one C + C group (‘NP16%’, *n* = 14) still received the normal‐protein diet, which contained 16% protein. Two C + C groups received a ‘low‐protein’ diet with either 8% protein (‘LP8%’, *n* = 14), that is, 50% of the protein content of the normal‐protein diet, or 12% protein (‘LP12%’, *n* = 14), that is, 75% of the protein content of the normal‐protein diet. The last two groups received a ‘high‐protein’ diet with either 24% protein (‘HP24%’, *n* = 14), that is, 150% of the protein content of the normal‐protein diet, or 32% protein (‘HP32%’, *n* = 14), that is, 200% of the protein content of the normal‐protein diet. All the diets were available *ad libitum*. In addition to the content of protein in the different diets, we also computed the cumulative protein intake by measuring the total protein intake of each rat during the 9 days of the intervention, thereafter referred as ‘cumulative protein intake’.

#### Chemotherapy

The chemotherapy cycles were started at D0, that is, 14 days after tumour implantation and 2 days after receiving the different diets (*Figure* 1). The tumour was treated with a combination of irinotecan (7‐ethyl‐10‐[4‐(1‐piperidino)‐1‐piperidino]carbonyloxy‐camptothecin; CPT‐11) and five fluorouracil (5‐FU) solutions (Centralized Cytotoxic Drug Reconstitution Unit (URCC), Michalon University Hospital, Grenoble, France). One cycle of chemotherapy corresponds to an intraperitoneal injection of CPT‐11 (50 mg/kg; Medac S.A.S, Lyon, France) at D0 and D7, followed by an intraperitoneal injection of 5‐FU (50 mg/kg; Accord Healthcare, Lille, France) the day after (D1 and D8).^20^, ^21^, ^22^ Fifteen minutes before each injection of CPT‐11, the rats received an injection of atropine (1 mg/kg; Aguettan, Lyon, France) to prevent and relieve early cholinergic symptoms.

### Euthanasia and sample collection

At D9, rats in post‐absorptive state (i.e. 3 h of fasting) were euthanized by decapitation.

Blood was collected in tube of lithium heparin, centrifuged (2000 *g* for 15 min at 4°C), and plasma was aliquoted: a part was frozen in liquid nitrogen and stored at −80°C until analysis and a part was treated (see below) and then stored at −80°C until amino acids measurements. The pellets were directly used for immune exploration (see below).

Thymus, kidney (left), heart, pancreas, adrenal gland (left), muscles (*extensor digitorum longus* and *soleus*; right and left), urogenital, retroperitoneal, subcutaneous and brown adipose tissue were removed and weighed. The proximal part of jejunum, ileum and colon were scraped and mucosa were collected and weighed. Only jejunum and ileum mucosa were frozen in liquid nitrogen for biochemical analysis. *Tibialis* (right and left) and the liver were weighed, frozen in liquid nitrogen and stored at −80°C for further analysis. The entire weight of the dissected tumour was recorded, and three samples of the tissue were taken at the tumour margin, avoiding any necrotic central part: Two of them were frozen in liquid nitrogen for biochemical assays, and the third one was placed, like Peyer's patches which were removed from the jejunum, in 2 mL of RPMI1640 medium containing 10% FBS and 1% antibiotics for immune analysis. The spleen was weighed and collected in RPMI1640 containing 1% antibiotics for immune study.

Body composition was determined as follows: the total fat‐free mass was included the heart, kidney, pancreas, adrenal gland, liver, spleen, thymus, muscles, viscera, skin, head and carcass weight; the total fat mass included the urogenital, retroperitoneal and subcutaneous adipose tissue weight.

### Plasma amino acids

After euthanasia, plasma (see above) was deproteinized with 10% (*w*/*v*) sulfosalicylic acid and centrifuged (10,600 *g* for 10 min at 4°C). Amino acid (AA) concentrations were measured by UPLC‐MS (Acquity UPLC H‐Class QDa, Waters) using a CORTECS UPLC C18 column after a derivatization step (AccQTag Ultra®kit, Waters).

### Protein content

Frozen *tibialis*, liver, tumour, jejunal and ileal mucosa were homogenized in 10 volumes of ice‐cold 10% trichloroacetic acid with 0.5 mmol/L EDTA. After delipidation with acetone, the pellets were solubilized in NaOH 1N (4 mL per 0.1 g) overnight at 37°C, and total protein content was determined using a BCA kit (Pierce™ BCA Protein Assay Kit; ThermoScientific, Rockford, IL).

### Protein expression

#### Protein extraction

Frozen *tibialis*, liver, tumour, jejunal and ileal mucosa were homogenized at 4°C in extraction buffer (Mammalian buffer [Merck KGaA, Darmstadt, Germany] with dithiothreitol 1 mM, protease inhibitor 1×, phosphatase inhibitor 1×, EDTA 1 mM, EGTA 1 mM) using a ball extractor (30 Hz for 2 × 1 min at 4°C). After centrifugation (20,800 *g* for 20 min at 4°C), the supernatant was collected and assayed using the BCA kit (ThermoScientific) allowing to standardize the samples with 30 μg of proteins per 10 μL of diluted solution of 3× Blue Loading Buffer (187.5 mM Tris–HCl (pH 6.8 at 25°C), 6% (*w*/*v*) SDS (sodium dodecyl sulphate), 30% glycerol, 0.03% (w/v) bromophenol blue) and 30× Reducing Agent (1.25 M dithiothreitol). The dilution was heated for 10 min at 95°C, centrifuged, and the supernatant was frozen.

#### Protein separation

Extracted proteins were loaded onto a SDS–polyacrylamide gel electrophoresis (15%) and transferred onto a nitrocellulose membrane (AmershamTM Protran TM; GE Healthcare, Germany). Proteins were revealed by staining the membrane with Ponceau S dye (Sigma‐Aldrich). After incubation in blocking buffer (10 mM Tris–HCl pH 8.0, 150 mM NaCl, 0.05% Tween 20, 5% milk powder), the membranes were incubated overnight at 4°C with primary antibody. Primary antibody (see the reference below) was then removed and a 1 h incubation was done with horseradish peroxidase‐conjugated mouse or rabbit secondary antibody (1:10 000 or 1:5000 dilution, respectively; Jackson ImmunoResearch Laboratories, Baltimore, MD). Immunoblots were visualized on an ImageQuant LAS4000 system (GE Healthcare, Buckinghamshire, UK) using the ECL Select™ Western Blotting Detection Reagents (GE Healthcare). Western blot images are illustrated in *Figures* S1 and S2.

#### mTOR pathway activation

mTOR pathway activation was analysed with the phosphorylated form of 4E‐BP1 on serine 65 (1:1000 dilution; Cell Signalling Technology, Ozyme, France). This target was chosen as well representative of mTORC1 activation.^26^, ^27^ Results obtained were normalized by the total form of anti‐4E‐BP1 (1:1000 dilution; Cell Signalling Technology).

#### Protein synthesis

The SUnSETmethod is a new technique to measure relative protein synthesis which has been compared with classical tracers' methods. Indeed, Goodman *et al*. demonstrated that puromycin labelling compared with the flooding dose method (using ^3^H‐phenylalanine) displays identical results.^28^, ^29^ In brief, 30 min before euthanasia, puromycin was injected by intraperitoneal way.

Protein synthesis was quantified using the anti‐puromycin antibody clone 12D10 (1:1000 dilution; Millipore, Temecula, CA) and normalized over Ponceau S quantification.

#### Proteolytic markers

Atrogin‐1 and Murf‐1 encode for components of the ubiquitin‐proteasome pathway.^30^ They were quantified using anti‐Atrogin‐1 (1:1000 dilution) and anti‐Murf‐1 (1:1000 dilution) antibodies (Abcam, Cambridge, UK), and normalized over Ponceau S quantification.

### Immune analysis

After centrifugation, the pellet was diluted with PBS and 200 μL was collected to be labelled with antibodies as described below. The organs (spleen, Peyer's patches and tumour) were crushed using a syringe plunger, or chopped with scissors before being filtered through a sieve. The cell suspensions obtained were washed with complete RPMI1640 medium containing 10% FBS and 1% antibiotics (except the spleen cells which were washed in complete RPMI1640 medium without FBS) then centrifuged at 600 *g* for 5 min at 4°C. The pellets, including spleen cells, were suspended in 5 mL of complete medium. A subsample of 200 μL was collected, diluted in 300 μL of PBS, and centrifuged at 600 *g* for 5 min at 4°C. The pellets were incubated in 100 μL of 1× Viobility fixable dye (Miltenyi, Bergisch Gladbach, Germany) for 20 min at room temperature in the dark. After dilution in 400 μL of PBS, the cell suspensions were centrifuged. The pellets were incubated in the mix of antibodies freshly prepared in PBS‐10%FBS for 20 min at 4°C in the dark (including samples for isotypes control and Fluorescence Minus One control) (CD25‐BV421, CD3‐BV605 and CD161‐BV786 from BD Biosciences; CD45R‐VioBright‐FITC, CD11b/c‐PE and RT1B‐PerCP‐Vio700 from Miltenyi; CD4‐PE‐Cy7, CD8a‐APC and CD45‐APC‐Cy7 from Biolegend). Cells were washed twice in PBS‐10%FBS and then fixed in 1× FACS lysing buffer (BD Biosciences). After two washes in PBS, 250 μL aliquots of the samples were kept for 48 h at 4°C in the dark before analysis in a FACS BD‐LSRII flow cytometer (BD Biosciences, Le Pont‐De‐Claix, France) using BD FACSDiva 6.3.1 software for data acquisition and Diva software for analysis. The analysed immune populations are described in *Table* S1.

### Statistical analysis

Results are reported as means ± SEM. A *t*‐test was used to study the C + C effect (between the Ctrl group and the NP16% group). A one‐way ANOVA was used to test for effects of protein diets. Normality and homogeneity of the variance were verified prior to statistical analysis. If the normality test (Shapiro–Wilk) failed (at *P* < 0.05), a Mann–Whitney test and a Kruskal–Wallis one‐way analysis were used. Differences were considered statistically significant at a *P*‐value < 0.05. In addition, we considered the HP24% and the HP32% groups as a single group with ‘high protein diet’, which has been compared with the C + C control group (NP16%) and then with the ‘low protein diet’ group (i.e. LP8% + LP12%).

Linear regression models were used to assess the association between the cumulative protein intake and several markers between D0 and D9 of (i) the rodents characteristics including body weight, total fat mass and total fat‐free mass and (ii) the tumour growth (considered individually), including tumour weight and tumour volume. Finally, the weight loss between D0 and D9 has been considered as a dichotomous variable based on the median of weight loss and whatever the C + C group. Several reasons justified this choice: first, the variability in body weight during the study experimental period is highly heterogeneous between rodents of all groups and also heterogeneous in each rodent group. Second, we prefered to apply a statistical defined threshold to compare the animals as there was no clinical thresholds to better describe this variable. In that case, we chose to apply the median of all data to respect a similar sample size in each group. The SAS statistical package (version 9.4, SAS Institute Inc., Cary, NC, USA) was used for these analyses.

## Results

As explained above, Ward colon tumour‐bearing rats were fed with isocaloric diets with low protein diet with 8% (LP8% group) or 12% (LP12% group) of caloric intake from protein, with standard diet with 16% (NP16% group) of proteins, or with high protein diet with 24% (HP24% group) or 32% (HP32% group) of proteins. Two days after, they received one cycle of chemotherapy, consisting of an injection of CPT‐11 (50 mg/kg) followed by 5‐fluorouracil (50 mg/kg) the day after, and a second cycle 1 week after. One healthy control group (*n* = 8) were fed a 16% protein diet. The rats were euthanized on day 9.

No diarrhoea has been observed, and no animals died during the study in any of the groups.

### Nutritional status

#### Food and protein intake

**Figure 2:**
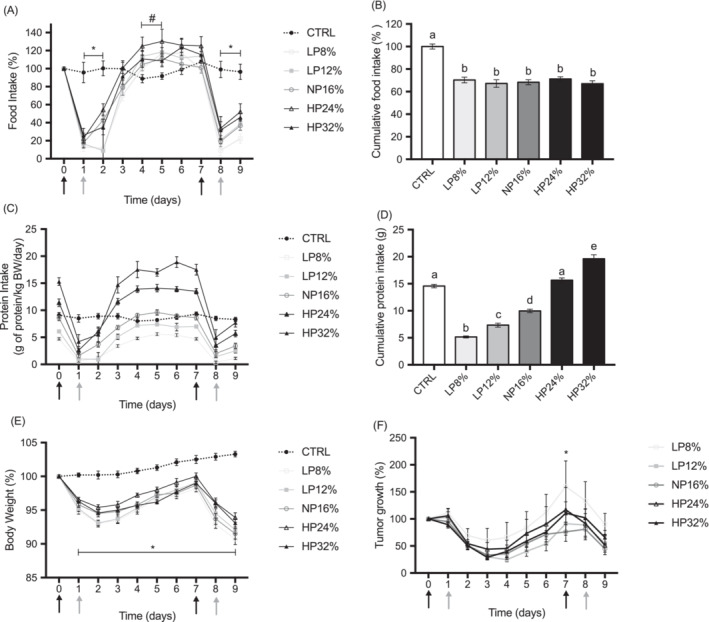
Food intake, protein intake and relative body weight of rats and relative tumour size. (A) Relative food intake compared to the first day of chemotherapy for each animal, (B) cumulative food intake at D9, (C) protein intake (g of protein/kg rat BW/day), (D) cumulative protein intake at D9, (E) relative body weight compared with the first day of chemotherapy for each animal, and (F) relative tumour size compared to the first day of chemotherapy for each animal. Control rats have received the normal‐protein diet (CTRL; *n* = 8) and tumour‐bearing rats received either the normal‐protein diet (NP16%, *n* = 14) or a low‐protein diet with 8% protein (LP8%, *n* = 14), a low‐protein diet with 12% protein (LP12%, *n* = 14), a high‐protein diet with 24% protein (HP24%, *n* = 14) or a high‐protein diet with 32% protein (LP32%, *n* = 14). The tumour‐bearing rats received the chemotherapy cycle of irinotecan (CPT‐11; 50 mg/kg) at D0 and D7 (dark grey arrow) and 5‐fluorouracil (5‐FU; 50 mg/kg) at D1 and D8 (light grey arrow). Results are expressed as mean ± SEM. Significant differences are reported as (A) and (E) * CTRL vs. LP8%, LP12%, NP16%, HP24% and HP32%, # CTRL vs. HP24%; (B) and (D) unalike letters; (F) * LP8% vs. NP16%.

Food intake decreased drastically in all five C + C groups at D1, then increased progressively until D5 to reach the same level as the control group in all the C + C groups, except the HP24% group which had higher food intake than controls (*Figure* 2A). All the C + C groups showed a second substantial decrease after the second cycle of chemotherapy (i.e. D8). From D0 to D9, food intakes were similar in all C + C groups, as illustrated by cumulative food intake, and lower than the Ctrl group (i.e. 70%) (*Figure* 2B).

Due to individual variations in overall food intake and chemotherapy‐induced anorexia, protein intake varied from 0.4 ± 0.2 to 5.6 ± 0.3 g/kg/day in LP8%‐group rats, from 1.0 ± 0.3 to 7.4 ± 0.3 g/kg/day in LP12%‐group rats, from 1.6 ± 0.4 to 9.6 ± 0.5 g/kg/day in NP16%‐group rats, from 2.6 ± 0.7 to 14.1 ± 0.7 g/kg/day in HP24%‐group rats, and from 4.2 ± 1.3 to 17.5 ± 1.0 g/kg/day in HP32%‐group rats (*Figure* 2C). Protein intake in the Ctrl‐group varied from 8.0 ± 0.6 to 9.3 ± 0.4 g/kg/day.

Measurements of cumulated protein intake at D9 (*Figure* 2D) showed that low‐protein‐diet rats and normal‐protein‐diet rats ingested less protein than the Ctrl‐group of rats. The HP24%‐group of rats ingested the same amount of protein as the Ctrl‐group of rats, and the HP32%‐group of rats ingested a higher amount of protein than the Ctrl‐group of rats.

As expected, cancer and chemotherapy does lead to anorexia, which was not modified by protein amount in the diet.

#### Body weight, body composition and organ weights

At D0, animal weight was the same in all six groups (*Table* 2). The weight of the rats of the five C + C groups decreased between D0 and D2, then increased between D2 and D7 and decreased again between D7 and D9 to fall to 91%–94% of initial body weight (*Figure* 2E). The difference in body weight between Ctrl‐group rats and NP16%‐group rats was significant from D1 to D9, and there was no difference between the five C + C groups (*Figure* 2E).

**Table 2:** Body and organ weights

	Cancer and chemotherapy effect			Protein diet effects				
	CTRL	NP16%	*P*	LP8%	LP12%	NP16%	HP24%	HP32%
Body weight (g)								
Initial (D0)	166 ± 3	165 ± 2	NS	167 ± 2 ^a^	166 ± 3 ^a^	165 ± 2 ^a^	166 ± 2 ^a^	165 ± 2 ^a^
Final (D9)	172 ± 3	151 ± 2	0.001	151 ± 3 ^a^	153 ± 3 ^a^	151 ± 2 ^a^	156 ± 3 ^a^	153 ± 2 ^a^
Fat‐free mass								
Organs (mg)								
Heart	508 ± 11	477 ± 8	0.0299	481 ± 6 ^a^	486 ± 9 ^a^	477 ± 8 ^a^	499 ± 9 ^a^	469 ± 7 ^a^
Kidney	562 ± 10	546 ± 8	NS	521 ± 7 ^a^	536 ± 10 ^a.b^	546 ± 8 ^a.b.c^	569 ± 11 ^b.c^	577 ± 10 ^b.c^
Pancreas	460 ± 58	430 ± 30	NS	379 ± 29 ^a^	431 ± 38 ^a^	430 ± 30 ^a^	402 ± 36 ^a^	424 ± 33 ^a^
Adrenal gland	29 ± 1	29 ± 1	NS	29 ± 1 ^a^	28 ± 1 ^a^	29 ± 1 ^a^	29 ± 1 ^a^	30 ± 1 ^a^
Liver (g)	6.1 ± 0.2	5.6 ± 0.1	0.049	5.2 ± 0.1 ^a^	5.6 ± 0.2 ^a.b^	5.6 ± 0.1 ^a.b^	5.9 ± 0.2 ^b^	5.7 ± 0.1 ^a.b^
Spleen	523 ± 13	495 ± 11	NS	476 ± 14 ^a^	470 ± 20 ^a^	495 ± 11 ^a^	500 ± 15 ^a^	490 ± 15 ^a^
Thymus	313 ± 68	85 ± 7	*<*0.001	77 ± 9 ^a^	76 ± 12 ^a^	85 ± 7 ^a^	84 ± 9 ^a^	80 ± 8 ^a^
Muscles (mg)								
EDL	74 ± 1	70 ± 1	NS	70 ± 1 ^a^	73 ± 1 ^a^	70 ± 1 ^a^	73 ± 1 ^a^	72 ± 1 ^a^
Tibialis	296 ± 5	286 ± 5	NS	280 ± 4 ^a^	289 ± 5 ^a^	286 ± 5 ^a^	293 ± 5 ^a^	290 ± 4 ^a^
Soleus	66 ± 1	67 ± 1	NS	64 ± 2 ^a^	66 ± 2 ^a^	67 ± 1 ^a^	67 ± 1 ^a^	65 ± 1 ^a^
Total fat‐free mass (g)	147 ± 2	135 ± 2	<0.001	133 ± 2 ^a^	135 ± 3 ^a^	135 ± 2 ^a^	138 ± 2 ^a^	135 ± 2 ^a^
Adipose tissue (g)								
Urogenital	5.2 ± 0.4	2.4 ± 0.2	<0.001	2.7 ± 0.2 ^a^	3.0 ± 0.2 ^a^	2.4 ± 0.2 ^a^	2.7 ± 0.1 ^a^	2.8 ± 0.2 ^a^
Retro	1.2 ± 0.1	0.5 ± 0.06	<0.001	0.6 ± 0.03 ^a^	0.6 ± 0.05 ^a^	0.5 ± 0.06 ^a^	0.6 ± 0.05 ^a^	0.6 ± 0.06 ^a^
Subcutaneous	7.2 ± 0.4	3.1 ± 0.3	<0.001	3.7 ± 0.3 ^a.b^	4.3 ± 0.3 ^a^	3.1 ± 0.3 ^b^	4.0 ± 0.3 ^a.b^	3.7 ± 0.2 ^a.b^
Total fat mass	13.6 ± 0.7	6.0 ± 0.4	<0.001	7.0 ± 0.5 ^a.b^	7.9 ± 0.4 ^a^	6.0 ± 0.4 ^b^	7.3 ± 0.4 ^a.b^	7.1 ± 0.4 ^a.b^
Brown (mg)	24 ± 1	14 ± 1	<0.001	14 ± 1 ^a^	15 ± 1 ^a^	14 ± 1 ^a^	13 ± 1 ^a^	13 ± 1 ^a^
Bowel (mg)								
Length (cm)	92 ± 1	90 ± 1	NS	90 ± 1 ^a^	89 ± 1 ^a^	90 ± 1 ^a^	90 ± 1 ^a^	90 ± 1 ^a^
Jejunal mucosa	281 ± 11	271 ± 20	NS	262 ± 17 ^a^	249 ± 15 ^a^	271 ± 20 ^a^	237 ± 14 ^a^	250 ± 12 ^a^
Ileal mucosa	270 ± 19	249 ± 16	NS	251 ± 11 ^a^	258 ± 17 ^a^	249 ± 16 ^a^	236 ± 18 ^a^	232 ± 13 ^a^
Colon mucosa	128 ± 17	132 ± 7	NS	143 ± 7 ^a^	133 ± 9 ^a^	132 ± 7 ^a^	129 ± 7 ^a^	148 ± 12 ^a^

Control rats have received the normal‐protein diet (CTRL; *n* = 8) and tumour‐bearing rats received either the normal‐protein diet (NP16%, *n* = 14) or a low‐protein diet with 8% protein (LP8%, *n* = 14), a low‐protein diet with 12% protein (LP12%, *n* = 14), a high‐protein diet with 24% protein (HP24%, *n* = 14) or a high‐protein diet with 32% protein (LP32%, *n* = 14). Two days later, for the tumour‐bearing rats, the first chemotherapy cycle was initiated: irinotecan (CPT‐11; 50 mg/kg) at D0 and 5‐fluorouracil (5‐FU; 50 mg/kg) at D1. One week later, they received the second cycle of chemotherapy (CPT‐11 at D7 and 5‐FU at D8). All rats were then sacrificed at D9 for analysis. Data are presented as mean ± SEM. Significant differences are grey‐backgrounded. For protein diet effects, values with different superscript letters are statistically different (*P* < 0.05).

EDL, extensor digitorum longus.

On the other hand, animals fed with a high protein diet (HP24% or HP32%) had a higher relative body weight compared with the animals fed with low (LP8% or LP12%) or normal protein diet (NP16%), whatever the tumour mass (β coef = 2.00, Standard error = 0.699, *P* = 0.006 and β coef = 2.25, Standard error = 0.79, *P* = 0.007, respectively). In the same way, cumulative protein intake was positively associated with relative body weight (*Table* 3). A high cumulative protein intake was associated with a lower odds of a body weight loss > to 7.2% (median of body weight loss) whatever the relative volume of the tumour or the weight of the tumour (OR = 0.806 (95% CI 0.724–0.899), *P* < 0.0001).

**Table 3:** Cumulative protein intake and impact on animals and tumour characteristics.

	β coefficient^a^	SE	*P*
C + C animals			
Relative body weight (%)^b^	0.332	0.083	0.0002
Fat‐free mass (g)	0.436	0.177	0.016
Fat mass (g)	0.088	0.054	0.111
Tumour of the C + C animals			
Relative volume (%)^c^	−1.240	1.275	0.334
Mass (g)	−0.023	0.029	0.431

C + C, cancer + chemotherapy; SE, standard error.

^a^ Bivariate linear regression models of the association between cumulative protein intake and each characteristic of interest.

^b^ Relative body weight of animals has been calculated at D9 as the percentage of body weight compared with D0 (the baseline).

^c^ Relative tumour volume has been calculated at D9 as the percentage of tumour volume compared with D0 and log transformed to ensure normality.

Decreased body weight of C + C rats was due to decreased total fat‐free mass (−8 ± 1%, *P* < 0.05) and decreased total fat mass (−56 ± 3%, *P* < 0.05) (*Table* 2). Concerning organ weights, only the liver, thymus and heart mass were lower in the NP16%‐group rats compared with the Ctrl‐group rats.

In the C + C groups, cumulative protein intake was positively associated with total fat‐free mass (*Table* 3) but not with total fat mass (*Table* 3). Concerning organ weights, the only significant differences between the five C + C groups were for kidney, liver and subcutaneous adipose tissue weights and total fat mass (*Table* 2).

Taken together, these data confirmed the cachexia observed in this animal model, and suggested that high protein diet improve animal body weight *via* an increased total fat‐free mass.

#### Tissue protein content, protein synthesis and proteolysis

In muscle, there were no significant differences in protein content. In the same way, protein synthesis in the post‐absorptive state was not different between the Ctrl‐group and the NP16%‐group, with no diet effect in cancer groups. As further confirmation, mTOR activation pathway (the key activator of protein synthesis) was not significantly different between the Ctrl‐group and the NP16%‐group, nor between the five C + C groups. Likewise, atrogin‐1 and MURF‐1 expression were not different between the Ctrl‐group and the NP16%‐group, nor between the five C + C groups (*Table* 4; see Western blot images in *Figure* S1).

**Table 4:** Protein metabolism

	Cancer and chemotherapy effect			Protein diet effects				
	CTRL	NP16%	*P*	LP8%	LP12%	NP16%	HP24%	HP32%
**Liver**								
Protein content								
mg	600 ± 66	557 ± 25	NS	500 ± 26 ^a^	583 ± 26 ^a^	557 ± 25 ^a^	597 ± 29 ^a^	586 ± 34 ^a^
g/100 g tissue	9.8 ± 0.9	10.0 ± 0.4	NS	9.5 ± 0.4 ^a^	10.4 ± 0.4 ^a^	10.0 ± 0.4 ^a^	10.2 ± 0.4 ^a^	10.2 ± 0.5 ^a^
Protein synthesis								
AU	0.36 ± 0.17	0.19 ± 0.11	NS	0.16 ± 0.03 ^a^	0.17 ± 0.03 ^a^	0.19 ± 0.10 ^a^	0.14 ± 0.06 ^a^	0.13 ± 0.04 ^a^
**Tibialis**								
Protein content								
mg	58.7 ± 2.4	52.6 ± 3.9	NS	47.3 ± 2.7 ^a^	50.9 ± 3.6 ^a^	52.6 ± 3.9 ^a^	51.9 ± 3.8 ^a^	52.1 ± 3.6 ^a^
g/100 g muscle	19.8 ± 0.7	18.4 ± 1.1	NS	17.2 ± 1.0 ^a^	17.8 ± 1.2 ^a^	18.4 ± 1.1 ^a^	17.7 ± 1.1 ^a^	18.1 ± 1.1 ^a^
Protein synthesis								
AU	0.74 ± 0.13	0.71 ± 0.16	NS	0.68 ± 0.11 ^a^	0.66 ± 0.16 ^a^	0.71 ± 0.16 ^a^	0.61 ± 0.10 ^a^	0.64 ± 0.06 ^a^
**mTORC1 activation**								
4EBP1 phospho (AU)	0.21 ± 0.05	0.15 ± 0.04	NS	0.14 ± 0.05 ^a^	0.14 ± 0.03 ^a^	0.15 ± 0.04 ^a^	0.14 ± 0.02 ^a^	0.15 ± 0.03 ^a^
Proteolyis								
Atrogin‐1 (AU)	0.18 ± 0.04	0.18 ± 0.03	NS	0.18 ± 0.04 ^a^	0.18 ± 0.03 ^a^	0.18 ± 0.03 ^a^	0.16 ± 0.03 ^a^	0.19 ± 0.04 ^a^
Murf‐1 (AU)	0.20 ± 0.09	0.20 ± 0.08		0.20 ± 0.09	0.16 ± 0.05	0.20 ± 0.08	0.17 ± 0.05	0.19 ± 0.07
**Jejunum**								
Protein content								
mg/10 cm	15.0 ± 1.5	11.6 ± 0.9	0.0440	12.5 ± 0.9 ^a^	12.0 ± 0.6 ^a^	11.6 ± 0.9 ^a^	11.3 ± 0.5 ^a^	11.3 ± 0.9 ^a^
g/100 mucosa	5.3 ± 0.4	4.5 ± 0.3	NS	4.6 ± 0.2 ^a^	5.0 ± 0.3 ^a^	4.5 ± 0.3 ^a^	4.8 ± 0.2 ^a^	4.6 ± 0.2 ^a^
Protein synthesis								
AU	0.61 ± 0.22	0.31 ± 0.18	NS	0.27 ± 0.06 ^a^	0.20 ± 0.18 ^a^	0.31 ± 0.18 ^a^	0.89 ± 0.06 ^a^	0.28 ± 0.10 ^a^
**Ileon**								
Protein content								
mg/10 cm	17.5 ± 2.0	15.9 ± 1.9	NS	15.5 ± 0.9 ^a^	15.6 ± 1.1 ^a^	15.9 ± 1.9 ^a^	14.1 ± 1.3 ^a^	14.2 ± 0.9 ^a^
g/100 g mucosa	6.9 ± 0.4	6.2 ± 0.3	NS	6.2 ± 0.2 ^a^	6.1 ± 0.1 ^a^	6.2 ± 0.3 ^a^	6.0 ± 0.3 ^a^	6.1 ± 0.2 ^a^
Protein synthesis								
AU	1.03 ± 0.40	0.99 ± 0.44	NS	1.09 ± 0.32 ^a^	0.81 ± 0.44 ^a^	0.99 ± 0.44 ^a^	0.89 ± 0.31 ^a^	1.31 ± 0.74 ^a^

Control rats have received the normal‐protein diet (CTRL; *n* = 8) and tumour‐bearing rats received either the normal‐protein diet (NP16%, *n* = 14) or a low‐protein diet with 8% protein (LP8%, *n* = 14), a low‐protein diet with 12% protein (LP12%, *n* = 14), a high‐protein diet with 24% protein (HP24%, *n* = 14) or a high‐protein diet with 32% protein (LP32%, *n* = 14). Two days later, for the tumour‐bearing rats, the first chemotherapy cycle was initiated: irinotecan (CPT‐11; 50 mg/kg) at D0 and 5‐fluorouracil (5‐FU; 50 mg/kg) at D1. One week later, they received the second cycle of chemotherapy (CPT‐11 at D7 and 5‐FU at D8). All rats were then sacrificed at D9 for analysis.

Data are presented as mean ± SEM. Significant differences are grey‐backgrounded. For protein diet effects, values with different superscript letters are statistically different (*P* < 0.05).

Concerning the liver and the intestine, there were no significant differences in tissue protein content, neither in protein synthesis in the post‐absorptive state, whatever the tissue and group considered (*Table* 4 and *Figure* S1).

In our study, protein metabolism in muscle and in gastrointestinal tract seems not modified neither by cancer associated to chemotherapy, nor by protein diet.

#### Plasma amino acids levels

Plasma arginine, asparagine, citrulline, isoleucine, leucine, methionine, proline, threonine and valine concentrations were lower in the NP16% group compared with the Ctrl rats, whereas plasma cystine and glycine concentrations were higher in the NP16% group compared with the Ctrl rats (*Table* 5).

**Table 5:** Plasma amino acid concentrations

(μmol/L)	Cancer and chemotherapy effect			Protein diet effects				
	CTRL	NP16%	*P*	LP8%	LP12%	NP16%	HP24%	HP32%
Arginine	146 ± 9	125 ± 5	0.05	104 ± 5^a^	120 ± 3^a,b^	125 ± 5^b^	117 ± 5^a,b^	115 ± 5^a,b^
Asparagine	70 ± 5	53 ± 3	0.003	59 ± 4^a^	59 ± 3^a^	53 ± 3^a^	57 ± 3^a^	57 ± 3^a^
Citrulline	80 ± 4	41 ± 5	0.0001	46 ± 5^a^	42^a^ ± 5^a^	41 ± 5^a^	38 ± 3^a^	37 ± 4^a^
Cystine	40 ± 3	47 ± 1	0.02	42 ± 2^a^	50 ± 1^b^	47 ± 1^a,b^	47 ± 2^a,b^	46 ± 2^a,b^
Glutamic acid	120 ± 8	99 ± 8	NS	111 ± 5^a^	100 ± 6^a,b^	99 ± 8^a,b^	92 ± 4^a,b^	87 ± 5^b,d^
Glutamine	894 ± 44	894 ± 28	NS	1001 ± 22^a^	959 ± 25^a,b^	894 ± 28^b,c^	888 ± 26^b,c^	844 ± 20^c^
Glycine	141 ± 8	228 ± 12	0.0001	250 ± 10^a^	239 ± 11^a,b^	228 ± 12^a,b^	207 ± 8^b.c^	188 ± 4^c^
Isoleucine	140 ± 11	105 ± 6	0.005	113 ± 6^a^	102 ± 4^a^	105 ± 6^a^	108 ± 4^a^	119 ± 7^a^
Leucine	237 ± 18	193 ± 10	0.03	200 ± 9^a^	186 ± 9^a^	193 ± 10^a^	196 ± 8^a^	214 ± 13^a^
Methionine	61 ± 4	53 ± 2	0.0585	51 ± 2^a^	52 ± 1^a^	53 ± 2^a^	53 ± 1^a^	52 ± 2^a^
Proline	350 ± 29	172 ± 16	0.0001	159 ± 17^a^	160 ± 12^a^	172 ± 16^a^	164 ± 16^a^	190 ± 21^a^
Serine	253 ± 13	276 ± 11	NS	321 ± 16^a^	338 ± 14^a^	276 ± 11^b^	252 ± 7^b,c^	227 ± 6^c^
Threonine	499 ± 30	351 ± 31	0.005	336 ± 23^a,b^	433 ± 39^a^	351 ± 31^a,b^	335 ± 23^a,b^	291 ± 14^b^
Valine	320 ± 24	240 ± 12	0.003	234 ± 10^a,b^	227 ± 9^a^	240 ± 12^a,b^	246 ± 10^a,b^	279 ± 16^b^
Total	4856 ± 275	4273 ± 158	0.06	4327 ± 147^a^	4443 ± 136^a^	4273 ± 158^a^	4097 ± 81^a^	4047 ± 146^a^

Control rats have received the normal‐protein diet (CTRL; *n* = 8) and tumour‐bearing rats received either the normal‐protein diet (NP16%, *n* = 14) or a low‐protein diet with 8% protein (LP8%, *n* = 14), a low‐protein diet with 12% protein (LP12%, *n* = 14), a high‐protein diet with 24% protein (HP24%, *n* = 14) or a high‐protein diet with 32% protein (LP32%, *n* = 14). Two days later, for the tumour‐bearing rats, the first chemotherapy cycle was initiated: irinotecan (CPT‐11; 50 mg/kg) at D0 and 5‐fluorouracil (5‐FU; 50 mg/kg) at D1. One week later, they received the second cycle of chemotherapy (CPT‐11 at D7 and 5‐FU at D8). All rats were then sacrificed at D9 for analysis. Only AAs with significantly differences are represented. Data are presented as mean ± SEM. For protein diets effect, values with different superscript letters are statistically different (*P* < 0.05).

Plasma glutamine, glycine, serine and threonine decreased with increased dietary protein intake. Conversely, valine increased with increased dietary protein intake.

Plasma amino acids levels were altered by cancer and chemotherapy, but not restored to normal with dietary proteins, suggesting the importance of protein quality.

### Tumour response to chemotherapy

#### Relative tumour volume, weight, protein content and protein synthesis

At D0, the five C + C groups had a similar mean tumour volume (*Data not shown*). Relative tumour size decreased in each group between D0 and D2 and between D7 and D8, that is, during the two chemotherapy cycles (*Figure* 2F), as reported by other authors using the same model.^21^, ^22^ There was no difference in relative tumour size between the five C + C groups (*Figure* 2F) and relative tumour volume was not modified by cumulative protein intake (*Table* 3).

Daily evolution of tumour growth according to cumulative protein intake showed no specific trend at any time (*Data not shown*).

Tumour mass was the same in the different groups (*Table* 6) and was not associated with cumulative protein intake (*Table* 3).

**Table 6:** Tumour‐response to chemotherapy

Tumour	Protein diets effect				
	LP8%	LP12%	NP16%	HP24%	HP32%
Weight (g)	1.9 ± 0.4^a^	1.5 ± 0.4^a^	1.3 ± 0.3^a^	1.4 ± 0.3^a^	1.9 ± 0.4^a^
Protein content					
mg	197 ± 36^a^	136 ± 36^a^	140 ± 28^a^	167 ± 37^a^	172 ± 35^a^
mg/g tissue	118 ± 4^a^	119 ± 2^a^	112 ± 3^a^	112 ± 2^a^	116 ± 2^a^
Protein synthesis					
AU	0.15 ± 0.03^a^	0.12 ± 0.03^a^	0.17 ± 0.06^a^	0.18 ± 0.05^a^	0.10 ± 0.02^a^

Tumour‐bearing rats received either the normal‐protein diet (NP16%, *n* = 14) or a low‐protein diet with 8% protein (LP8%, *n* = 14), a low‐protein diet with 12% protein (LP12%, *n* = 14), a high‐protein diet with 24% protein (HP24%, *n* = 14) or a high‐protein diet with 32% protein (LP32%, *n* = 14). Two days later, the first chemotherapy cycle was initiated: irinotecan (CPT‐11; 50 mg/kg) at D0 and 5‐fluorouracil (5‐FU; 50 mg/kg) at D1. One week later, they received the second cycle of chemotherapy (CPT‐11 at D7 and 5‐FU at D8). All rats were then sacrificed at D9 for analysis. Data are presented as mean ± SEM. Values with different superscript letters are statistically different (*P* < 0.05).

Protein content and synthesis did not change in response to protein loads (*Table* 6 and *Figure* S2).

Taken together, we could not detect any significant effect of nutrition on tumour response to chemotherapy.

### Immune system

Immune cell population was modified in the NP16% group, with higher CD4+/CD45 + and CD45R + RTB1/CD45 + populations in blood and lower CD3 + CD11+/CD45 + and CD3 + CD11 + RTB1+/CD45 + populations in spleen compared with the Ctrl group (*Figure* S2). Cancer and chemotherapy had no effect on immune cell populations in the Peyer's patches (*Figure* S3).

There was no effect of protein diets on immune‐system responses whatever the immune cell population or organ studied (*Figure* S3).

Immune system was only modified by cancer and chemotherapy, not by protein loads.

## Discussion

The role of protein intake in cancer is a matter of debate and has been for years.^5^, ^9^ It is well known that a high protein intake increases muscle mass in healthy people, but data in patients with cancer are too sparse to draw firm conclusions.^5^ Furthermore, the effects of modulated protein intake on tumour growth with parallel cancer treatment are scarcely documented. From a conceptual standpoint, an increase of protein intake would promote tumour growth, as cancerous cells are cells with a high turnover and thus high amino acid requirements. However, immune cells also have high AA requirements and they are the first line of defence against tumour growth. Thus, the final outcome of modulating protein intake is difficult to anticipate. Some papers have shown that a small amount of protein could reduce tumour growth by decreasing mTOR pathway activation^18^ or by decreasing the tumour immunosurveillance.^19^ However, all these papers have only limited value here, because the models used failed to include cancer treatment (chemotherapy, radiotherapy, etc). Moreover, the research needed is not possible in humans due to ethical reasons. To address this issue, we used a relevant animal model of cancer and chemotherapy from clinical setting, to evaluate the influence of protein load on tumour growth and cachexia. To the best of our knowledge, this is the first time that such study has been performed.

Although the effect seems modest, we were able to show that higher protein intakes are more favourable from a nutritional point of view. Unfortunately, it is difficult to compare our findings with clinical data, because (i) it is unethical to give a low‐protein diet to cancer patients and (ii) there is only little data available on the effects of high‐protein diets on muscle mass, and all of them associate high protein intake with higher energy intake, whereas in our study, the diets were iso‐caloric to specifically individualize the protein effect in our study [for reviews, see previous works^31^, ^32^, ^33^]. Several hypotheses can be advanced to explain the weak effect of protein load on muscle mass found here. First, for the conceptional basis of this original work, it was essential for all the groups to be iso‐energetic in order to individualize the effect of protein load, and to avoid energy effect. However, in such conditions, the nitrogen–calorie ratio (*Table* 1) for HP32% and HP24% groups (i.e. 50.1 and 74.9 kcal/g, respectively) was too low compared with the recommended values that are between 100 and 150 kcal/g for optimal assimilation of the nitrogen intake.^1^ Indeed, energy is needed to metabolize the proteins approached by the diet. We can assume that bringing more calories would lead to a better use of proteins to activate protein synthesis, and thus to an improvement of the nutritional status. Therefore, we can conclude that the low‐protein diets had no effect, and that high protein‐normo calorie diets had a modest effect on nutritional status, but further studies with the use of high‐protein and high‐energy diets are needed to give a definitive opinion on the interest of dietary proteins to improve nutritional status in cancer patients. Second, one may hypothesize that the duration of the diet intervention was too short (i.e 11 days) to induce a significant detrimental or beneficial effect on muscle mass. Actually, this is highly unlikely, because a short‐term nutritional intervention (3–4 days) is sufficient to affect protein homeostasis in other model of protein‐energy malnutrition.^34^, ^35^ Furthermore, in the same tumour‐bearing rats with chemotherapy, 9 days of diet with different type of fibres modified relative animal body weight, suggesting that 11 days of protein diet should be enough to improve protein metabolism.^36^ Third, protein intake *per se* cannot efficiently cover the specific AA requirements induced by cancer and chemotherapy. This is well illustrated by the AA profiles that were profoundly affected in the C + C rats and not restored to normal by the increase of protein intake, as well discussed by van der Meij *et al*.^37^ Quality of the dietary protein, and thus of AAs, seems to be important for muscle anabolism in cancer patients. More precisely, essential AA and branched‐chain AA are particularly efficient to activate MPS in cancer patient, like in healthy people.^37^, ^38^ This explains why a group of experts highlights that meat‐based proteins are more interesting than plant‐based proteins to improve muscle mass in cancer patients.^9^ One perspective for research would be to develop personalized nutritional support for cancer patients, as proposed by Berard *et al*. in a pioneering study on surgical patients (i.e. customized AA pattern in the artificial nutrition).^39^

As high‐protein intakes seemed to have beneficial effects on nutritional status, it was important to test their potential effects on tumour size—especially because expert‐group guidelines on nutritional support for cancer patients stipulate that ‘*Theoretical arguments that nutrients ‘feed the tumour*’ *are not supported by evidence related to clinical outcome and should not be used to refuse, diminish, or stop feeding*’.^2^ However, this assertion does not appear to be based on any hard data but is mainly based on expert opinion, as specified by Prado *et al*. and by Ford *et al*.^5^, ^9^ Moreover, this same group of experts recommends a protein intake of 1.2 to 1.5 g/kg/day. However, there are very few studies on the role of dietary protein in tumour growth in realistic conditions (associated with cancer treatment).

In our model, a low protein intake had no effect on tumour size, whereas two studies found that a low‐protein diet decreased tumour size.^18^, ^19^ However, in these studies, the animals received no treatment with any real clinical relevance. The protein effect observed in these studies, that is, decreased tumour size with low protein intake, could be too weak compared with the effect of chemotherapy, which would explain why we found no effect of protein intake levels on tumour growth size.

Concerning the tumour‐response to chemotherapy, even if our data support an absence of protein effects on the growth of Ward colon tumour and on CPT‐11/5‐FU‐based chemotherapy, it is impossible at present to extend these findings to other tumours and treatments. Additional studies on other models are mandatory. However, while all cancers and treatments may be different, the correlated undernutrition has always the same consequences. Furthermore, the metabolic changes and the modification of nutritional requirements may be different, but the resistance to renutrition will stay the same.

In terms of nutritional status, as discussed above, given that the diets tested here were iso‐caloric (to specifically individualize the protein effect), the nitrogen–calorie ratio in the high‐protein diet groups could be too low to enable meaningful assimilation of nitrogen. Further studies using high‐protein and high‐energy diets would help conclude on the effect of high‐protein diet on cachexia and tumour growth. Another limitation is the chemotherapy‐induced anorexia, which induces a low protein intake and thus leads to a situation where nutritional intakes fail to meet nutritional needs. Furthermore, the protein composition used here is also inadequate on a qualitative level, given the differences observed for the plasma AAs. Using artificial nutrition in order to normalize caloric and nutritional goals would be an instructive way forward to confirm our results.

## Conclusions

This study demonstrated, for the first time, in this realistic model of tumour‐bearing rats receiving chemotherapy and different protein diets, that low protein intake has no influence on nutritional status, while high protein intake improved, even if in a modest way, nutritional status. Moreover, this model allowed to show that different protein diets have no deleterious or beneficial effect on tumour response to chemotherapy, or on immune‐system response. Even if this work suggests that it is interesting to give high protein diet to cancer patients to improve nutritional status, without any risk concerning tumour response to chemotherapy, it must now be validated in other cancer models that include treatments. Our work is a first step towards realistic recommendations for patients, based on experimental data mirroring the conditions of real‐world clinical nutrition management.

## Funding

Martin Boutière and Charlotte Breuillard were supported by a grant from a non‐profit organization ‘Fonds pour les Maladies Chroniques’ and Charlotte Breuillard by a second grant from a non‐profit learned society ‘Société Francophone de Nutrition Clinique et Métabolique’ (SFNCM). The cost of the experiments was supported by these two grants and by recurrent grant from Univ. Grenoble Alpes. The authors of this manuscript certify that they comply with the ethical guidelines for authorship and publishing in the *Journal of Cachexia, Sarcopenia and Muscle*.^40^

## Conflict of interest statement

Martin Boutière, Cécile Cottet‐Rousselle, Céline Coppard, Karine Couturier, Catherine Féart, Morgane Couchet, Christelle Corne, Christophe Moinard and Charlotte Breuillard declare that they have no conflict of interest.

## Supporting information


**Figure S1.** Western blot images of tissue protein synthesis and muscle proteolysis. Western blot images (A) Tibialis total protein synthesis, (B) Tibialis 4EBP1 phosphorylation on serine 65, (C) Tibialis Atrogin1 expression, (D) Tibialis Murf1 expression, (E) Liver total protein synthesis, (F) Jejunal mucosa total protein synthesis and (G) Ileal mucosa total protein synthesis. (A) (C) (D) (E) (F) (G) Western Blot images of puromycin followed by ponceau membrane, (B) Western Blot images of 4EBP1 phosphorylation on serine 65 followed by total form of 4E‐BP1.

**Figure S2.** Western blot images of tumor protein synthesis. Western blot images of puromycin followed by ponceau membrane.

**Figure S3.** Immune exploration in blood

**Table S1** Cell populations analyzed for immune exploration
