# Supplementary material for: Protein intake in cancer: Does it improve nutritional status and/or modify tumour response to chemotherapy?
Source: J Cachexia Sarcopenia Muscle. 2023 Sep 4;14(5):2003–15. doi: 10.1002/jcsm.13276 (PMC10570073; doi:10.1002/jcsm.13276)
Supplement: Supplementary file 1 — Figure S1. Western blot images of tissue protein synthesis and muscle proteolysis. Western blot images (A) Tibialis total protein synthesis, (B) Tibialis 4EBP1 phosphorylation on serine 65, (C) Tibialis Atrogin1 expression, (D) Tibialis Murf1 expression, (E) Liver total protein synthesis, (F) Jejunal mucosa total protein synthesis and (G) Ileal mucosa total protein synthesis. (A) (C) (D) (E) (F) (G) Western Blot images of puromycin followed by ponceau membrane, (B) Western Blot images of 4EBP1 phosphorylation on serine 65 followed by total form of 4E‐BP1. [file JCSM-14-2003-s004.docx]

(A)


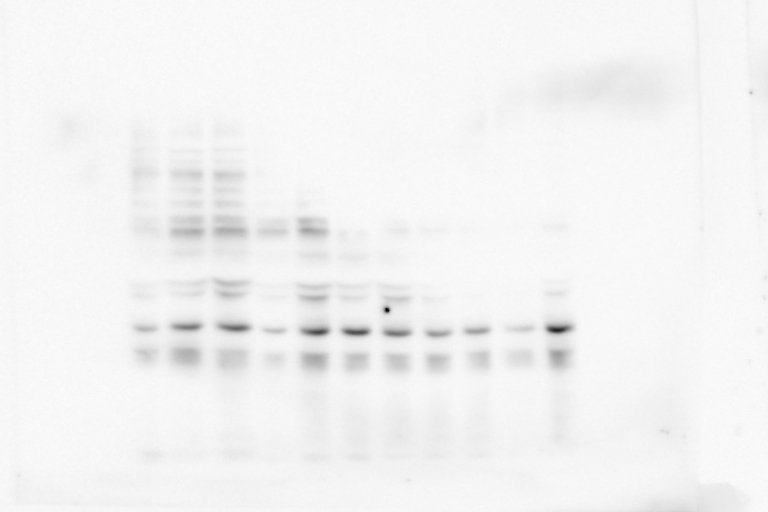

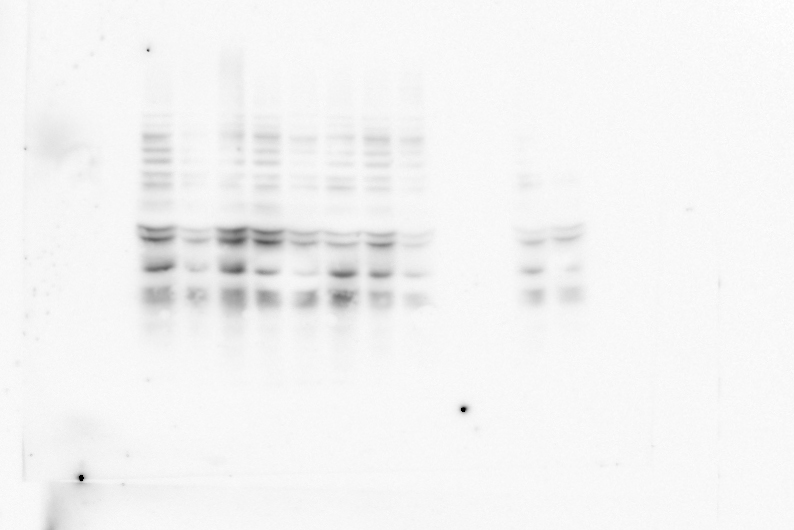

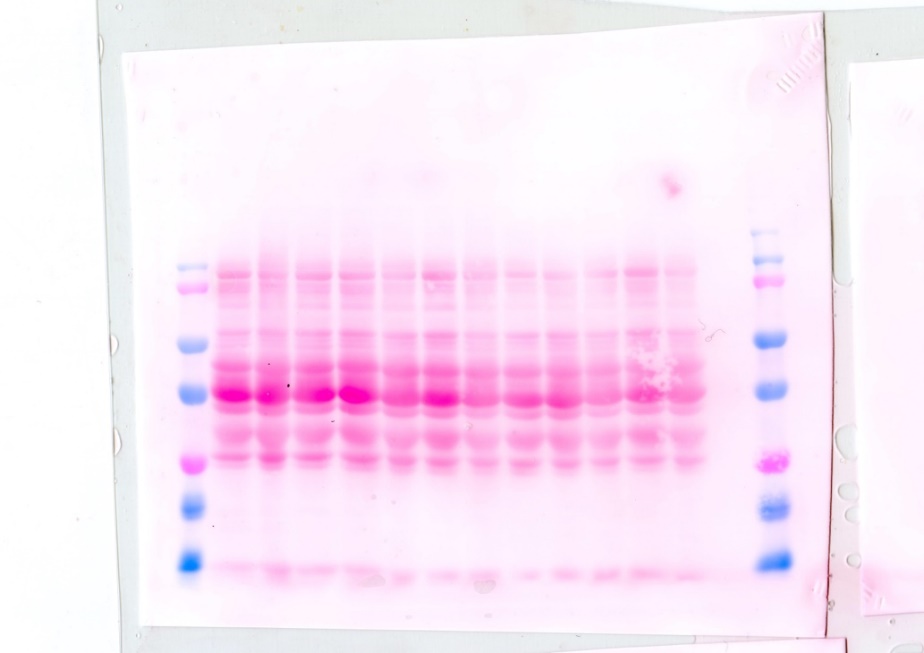

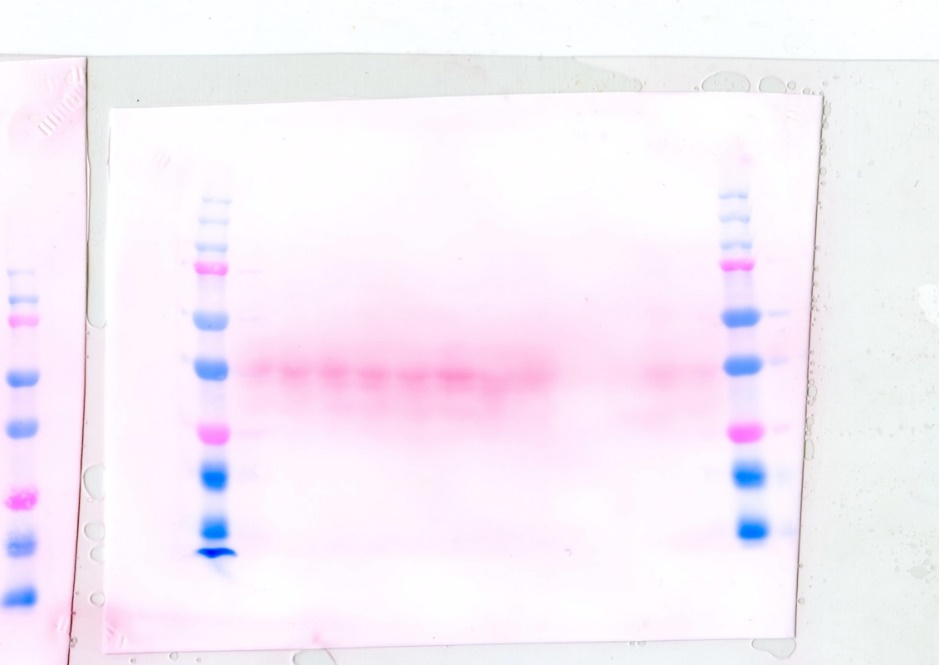


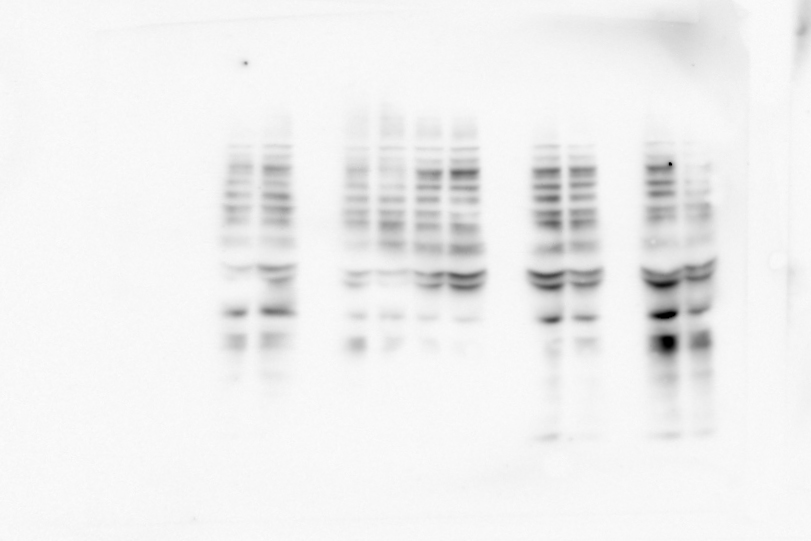

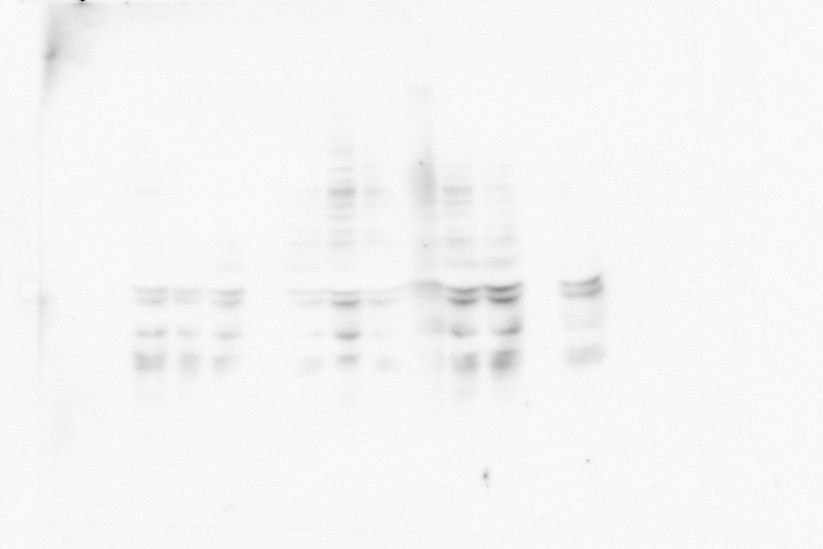

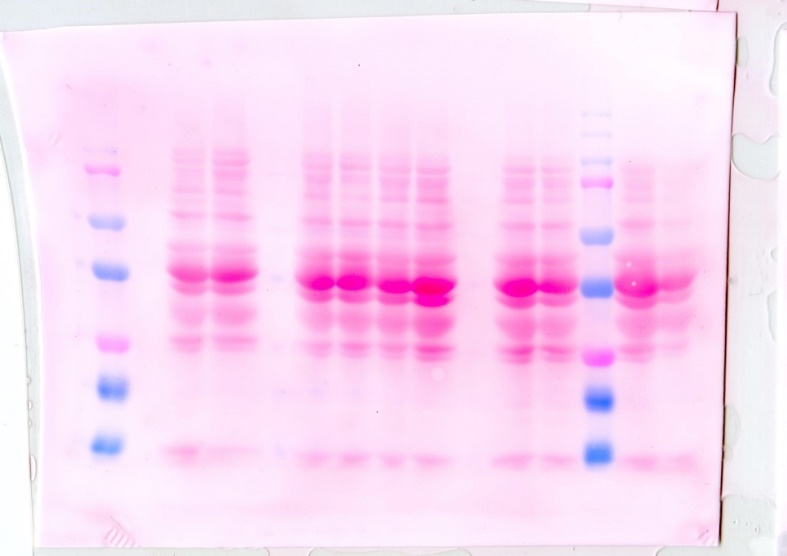

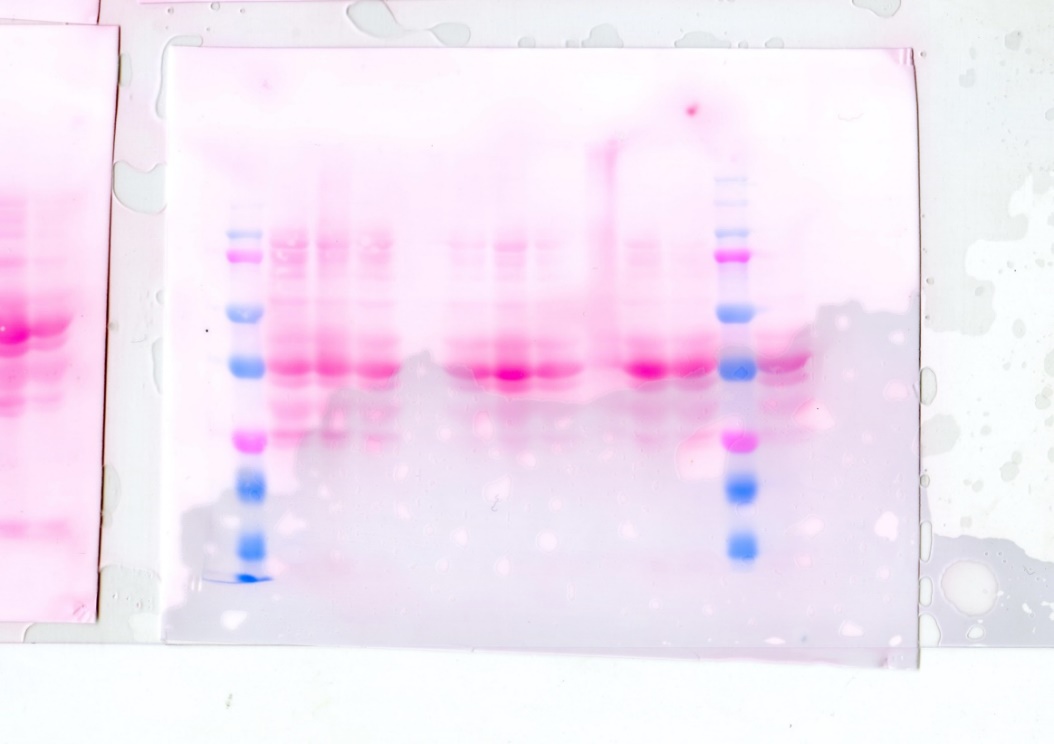


(B)


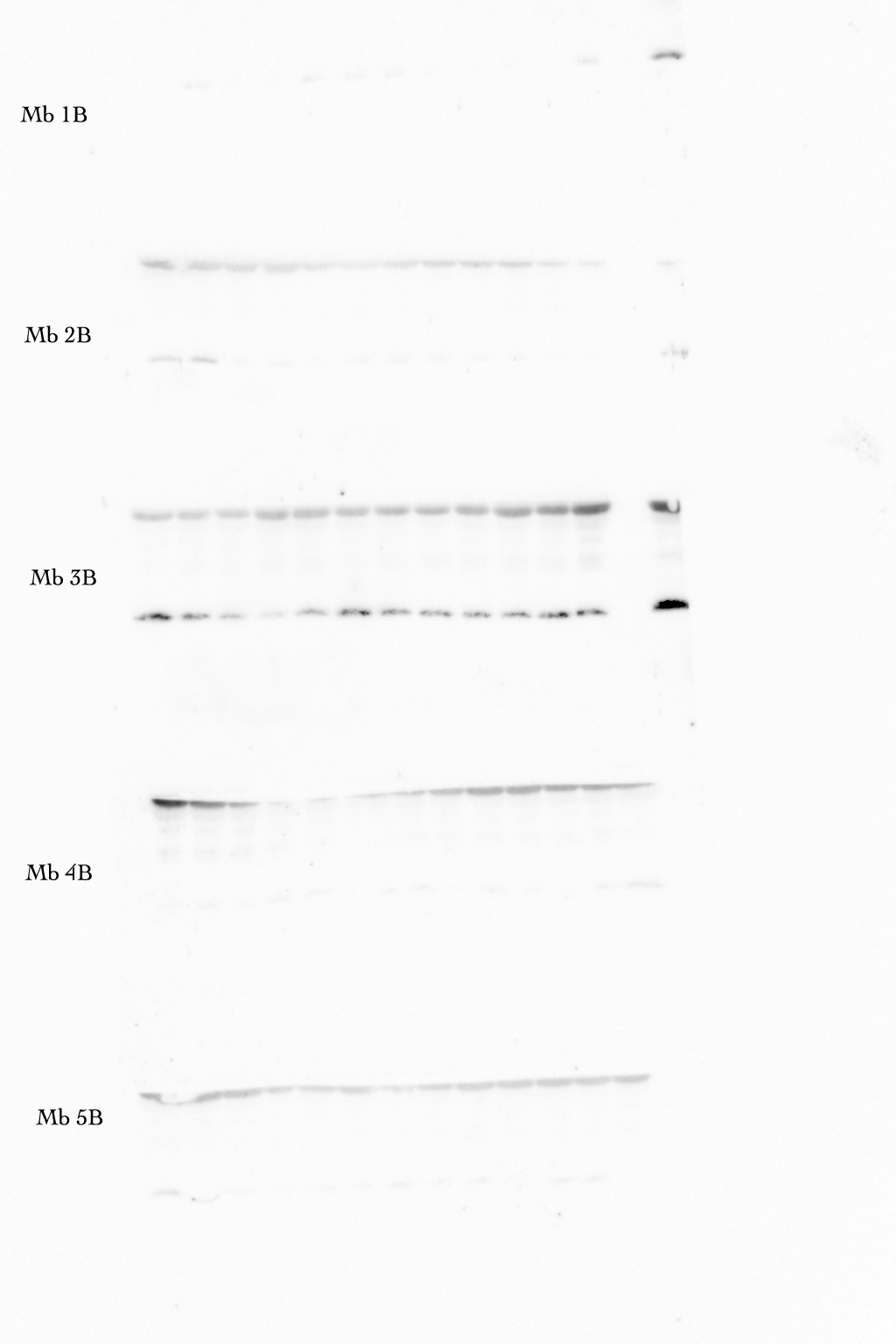

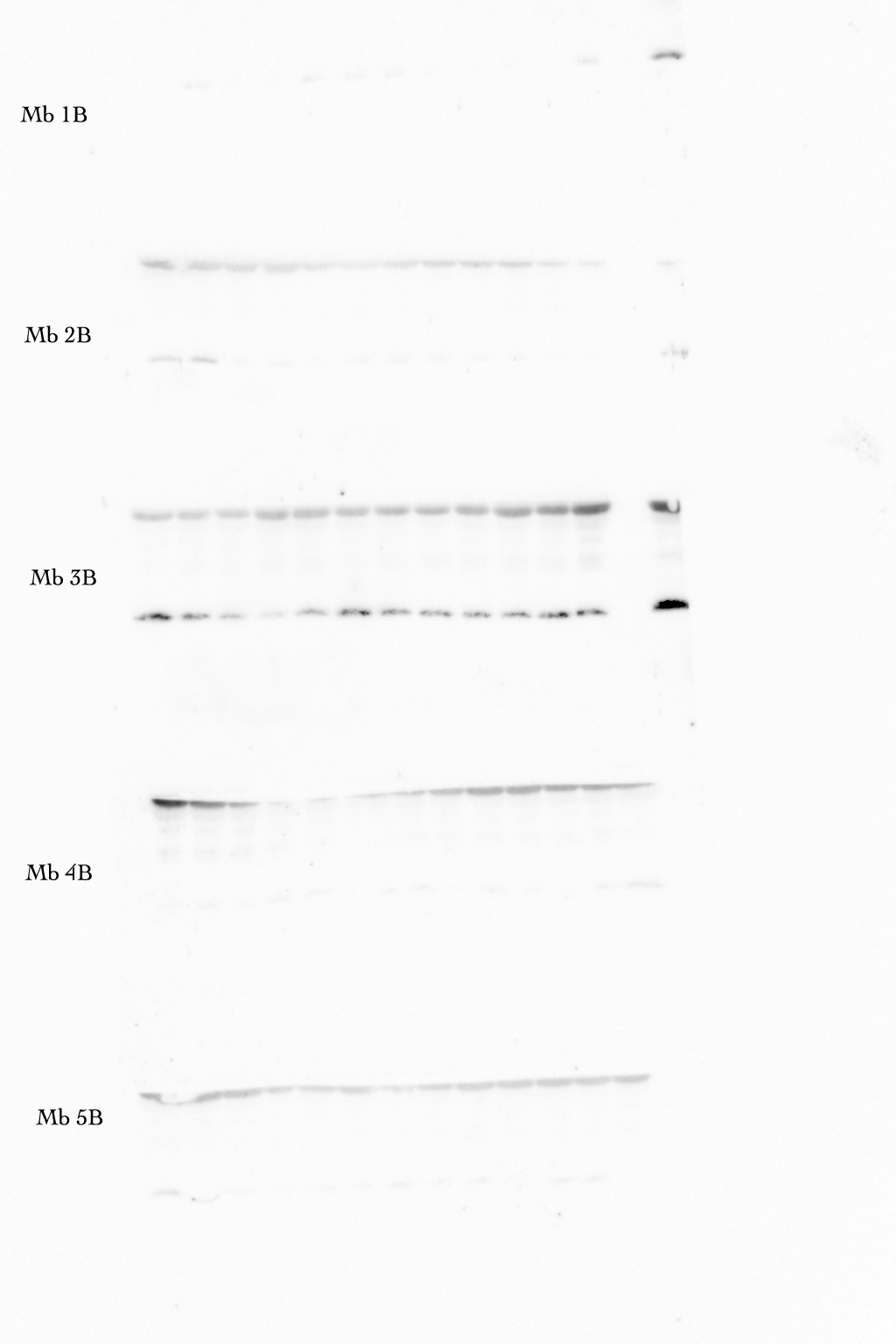

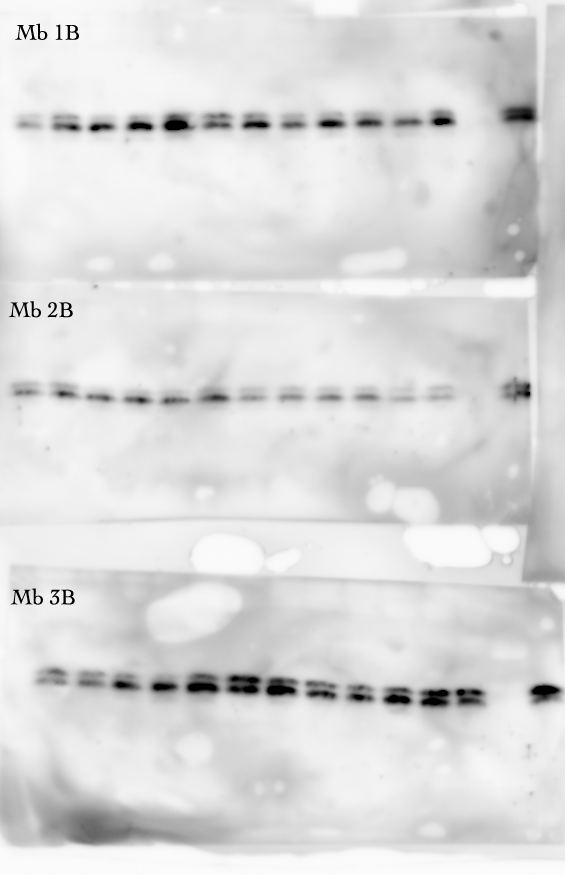

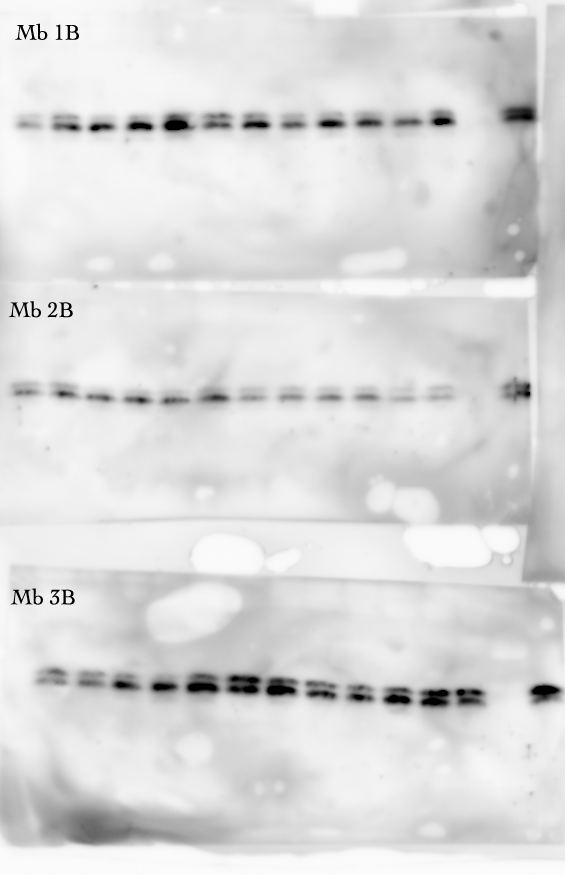


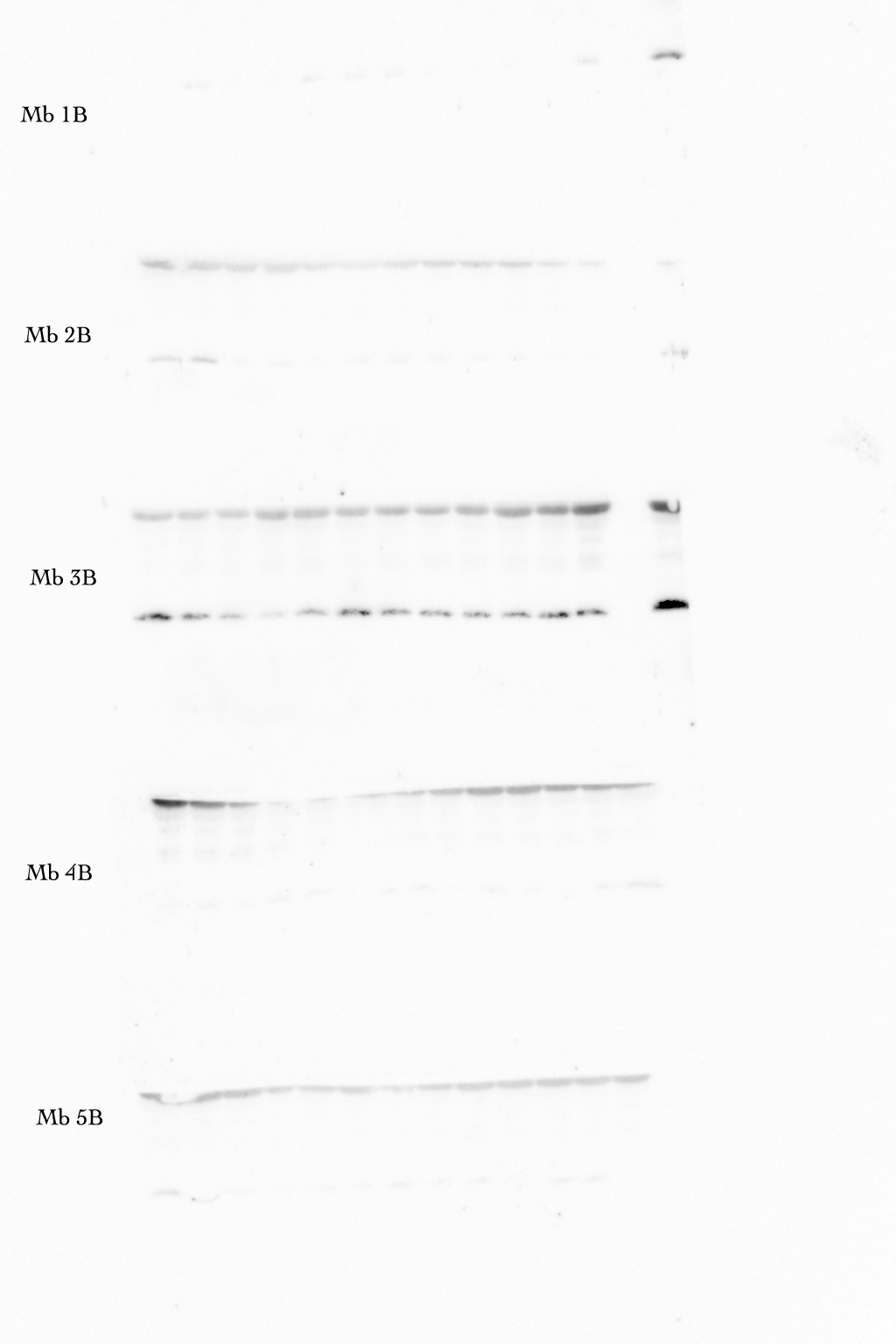

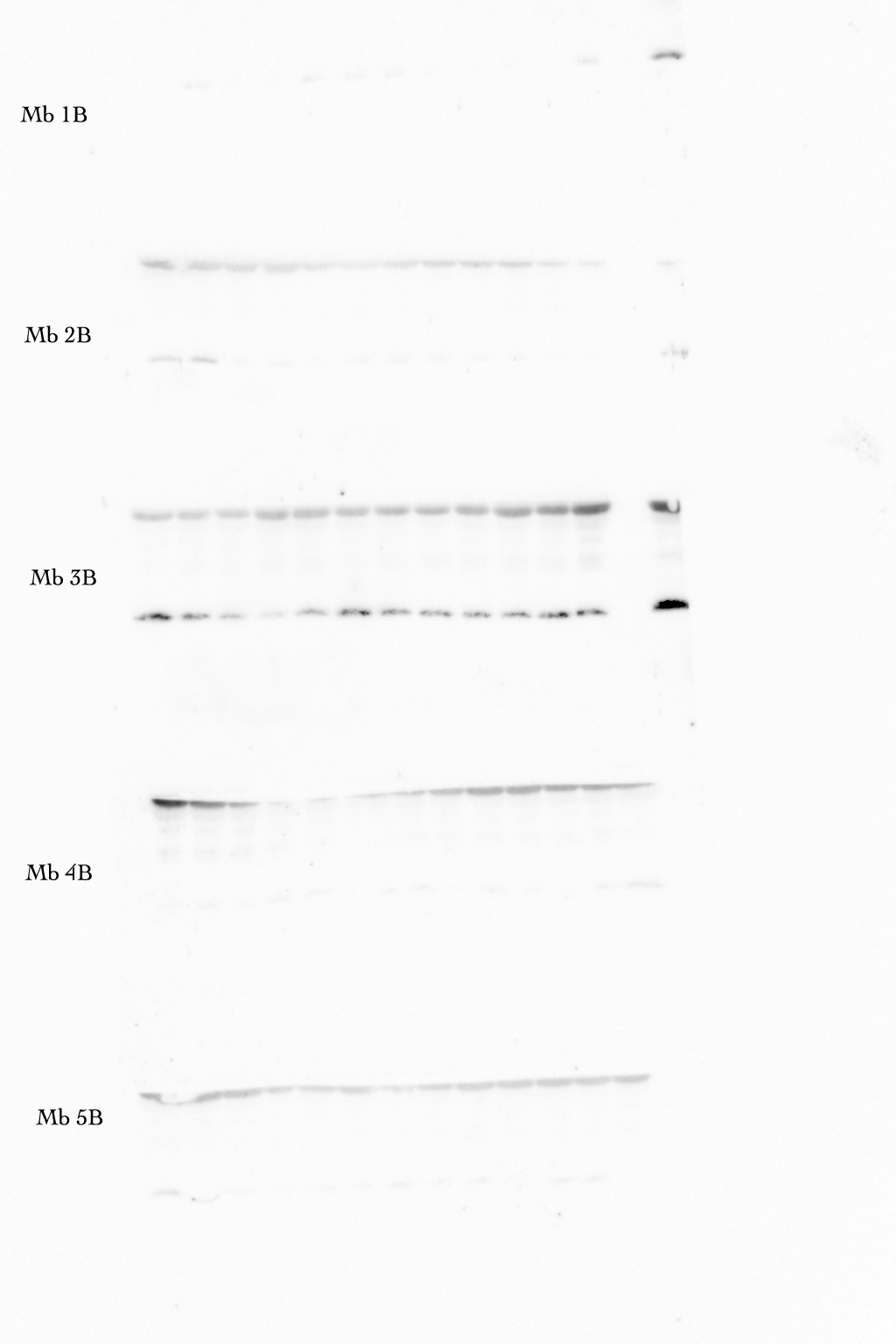

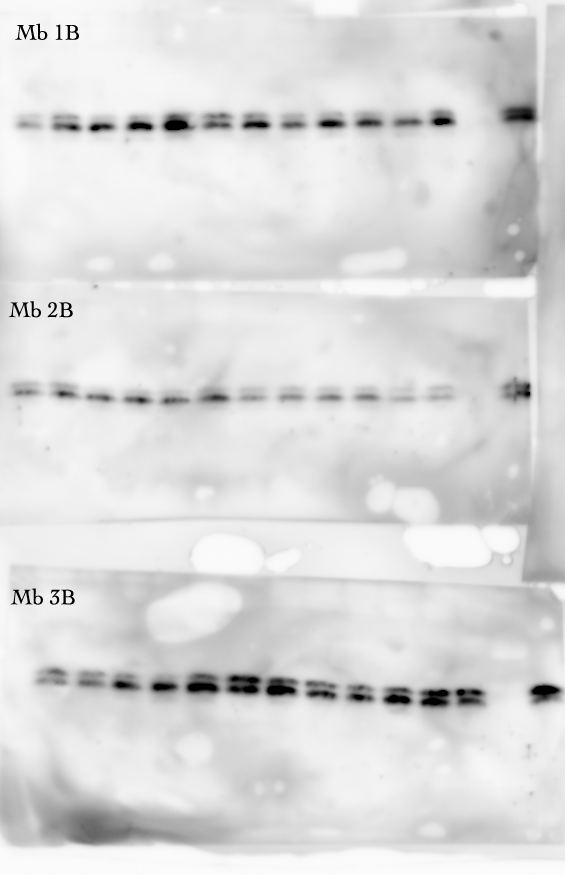

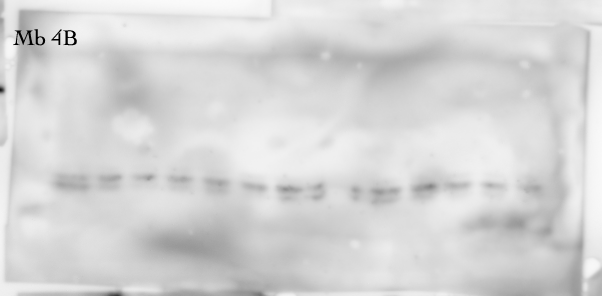


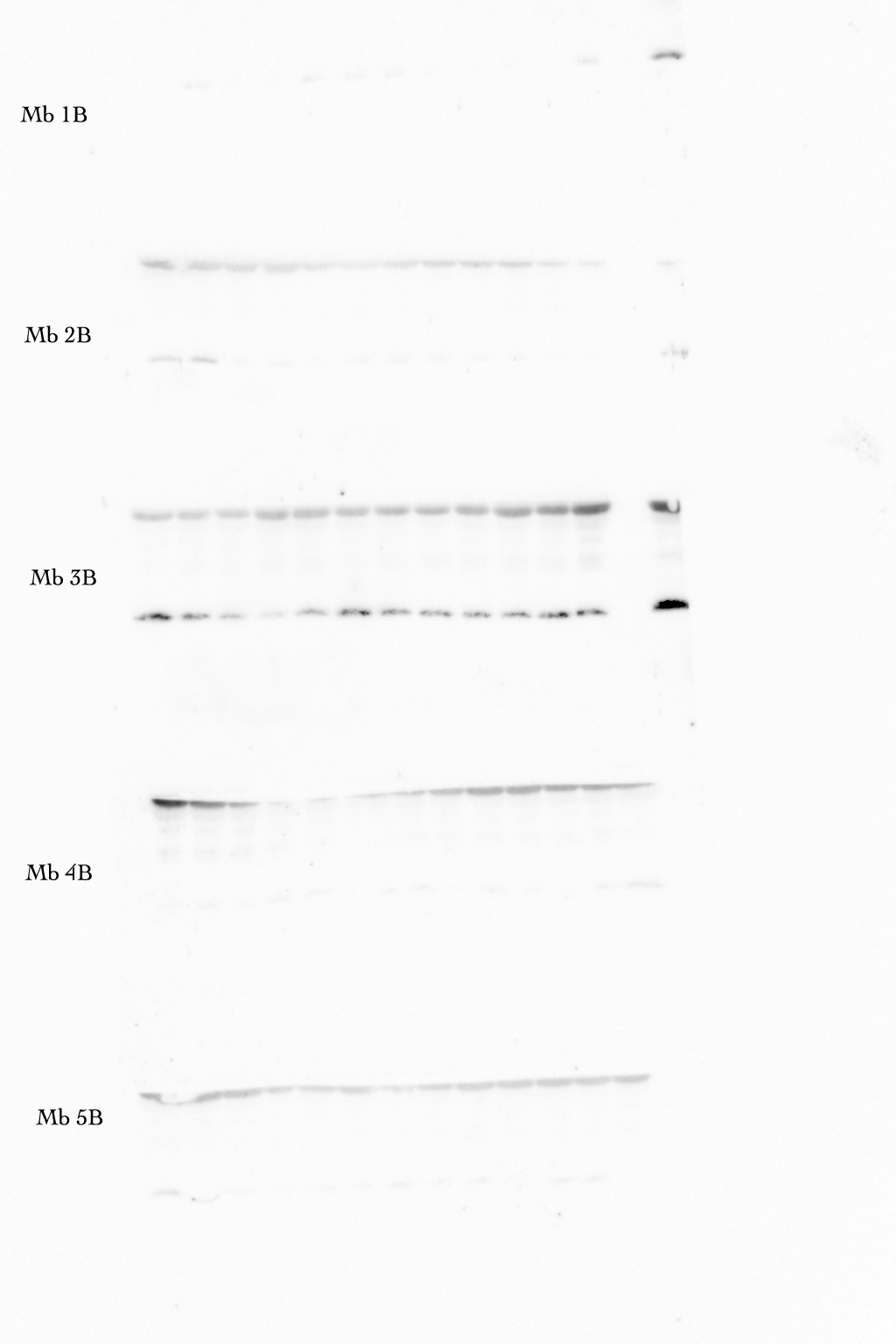


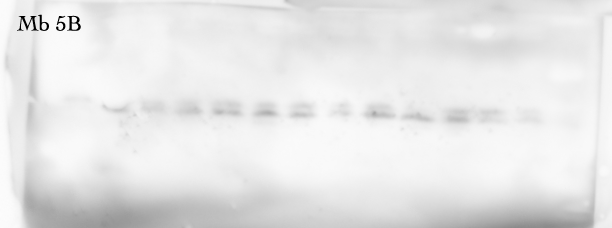
(C)


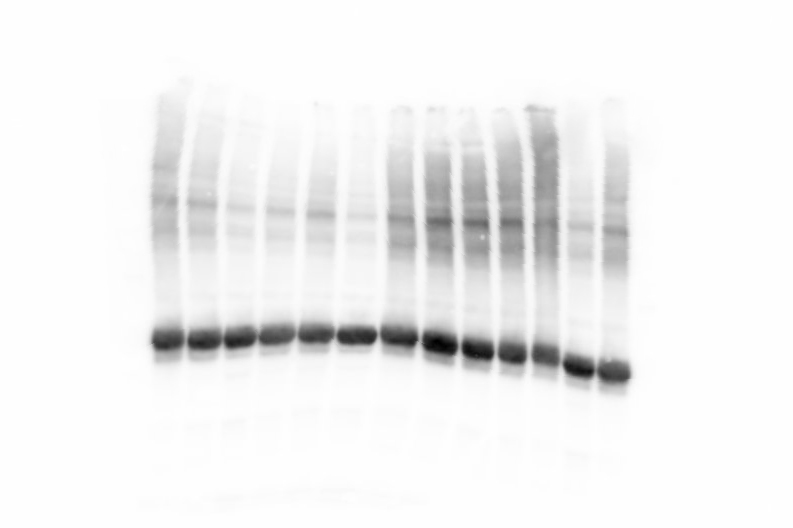

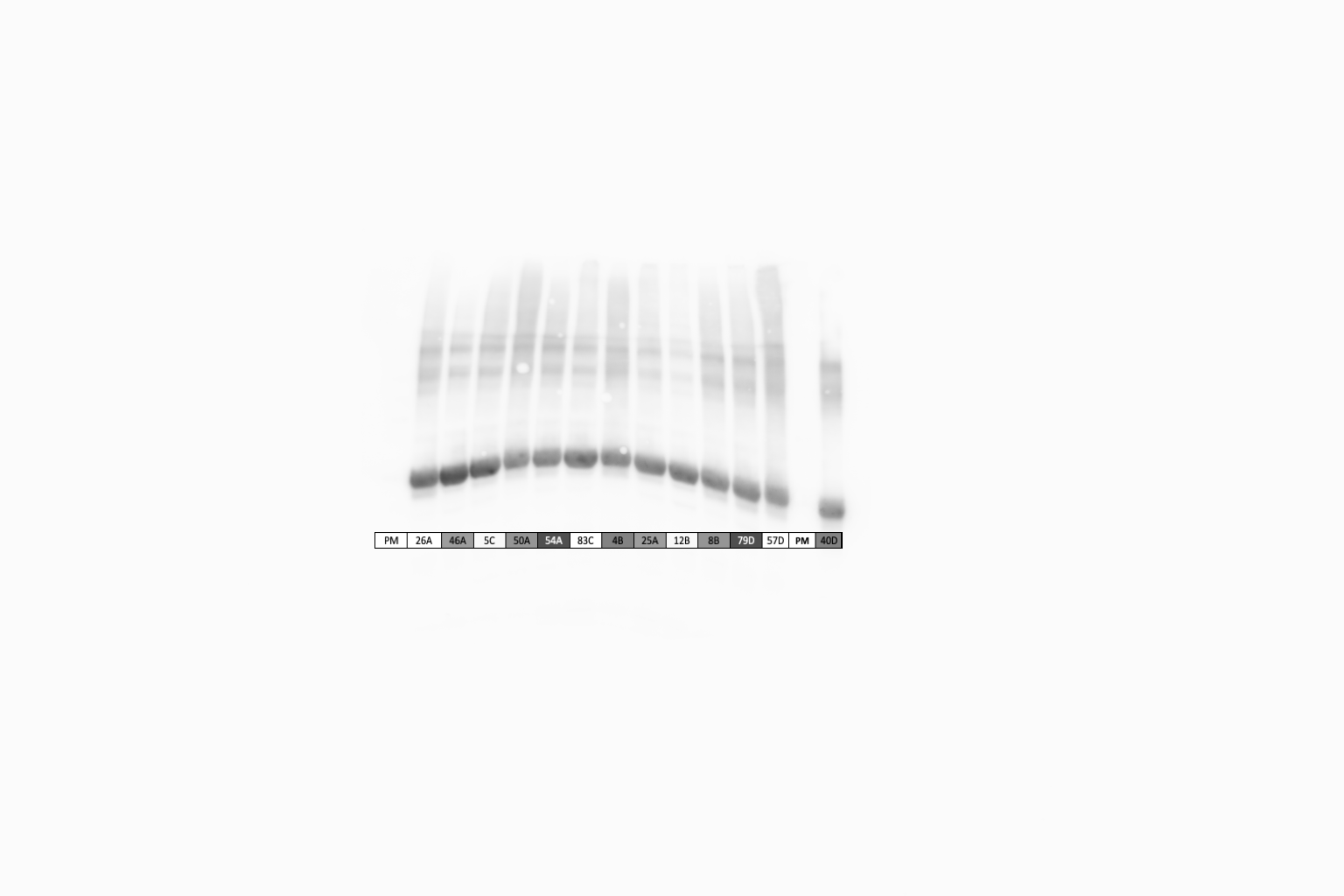


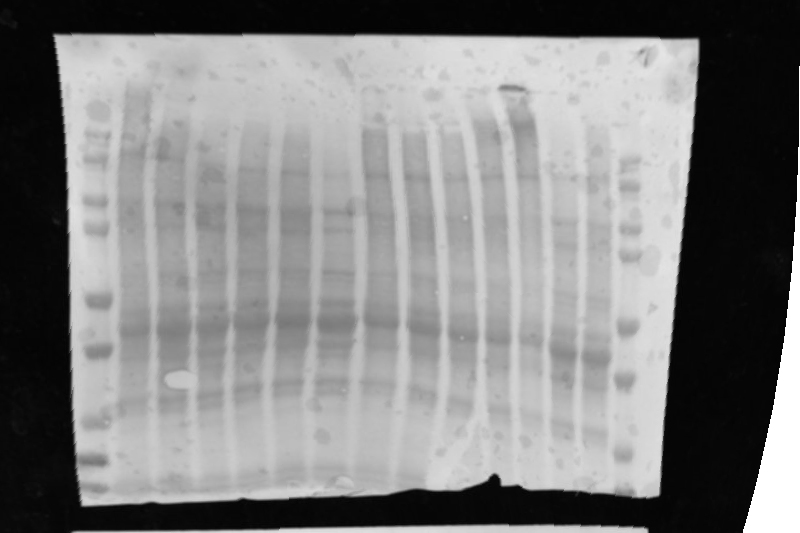

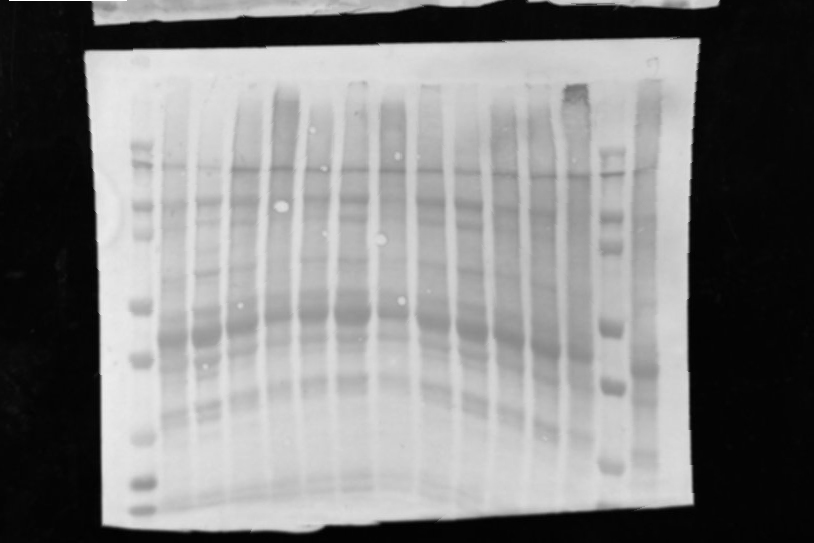


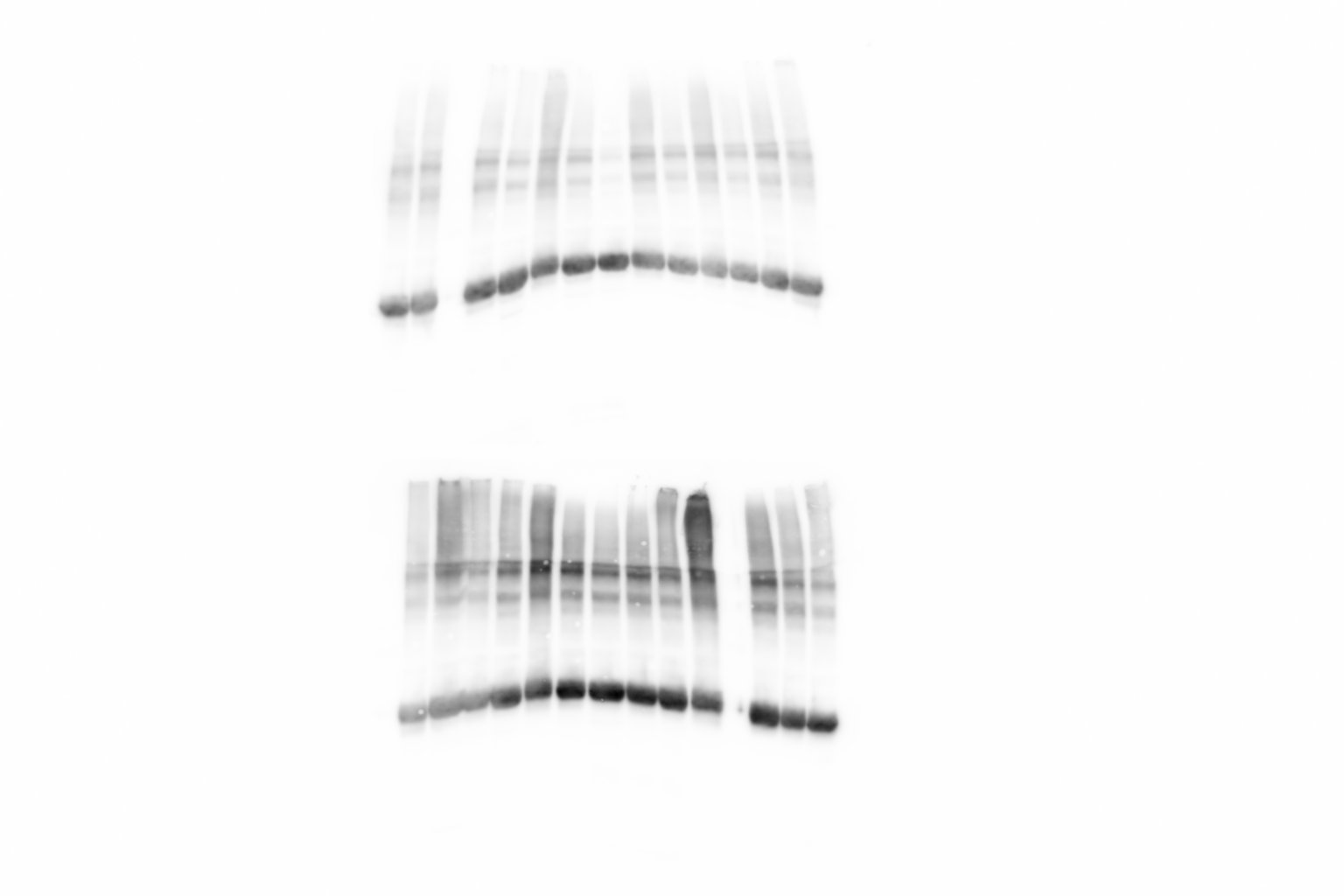

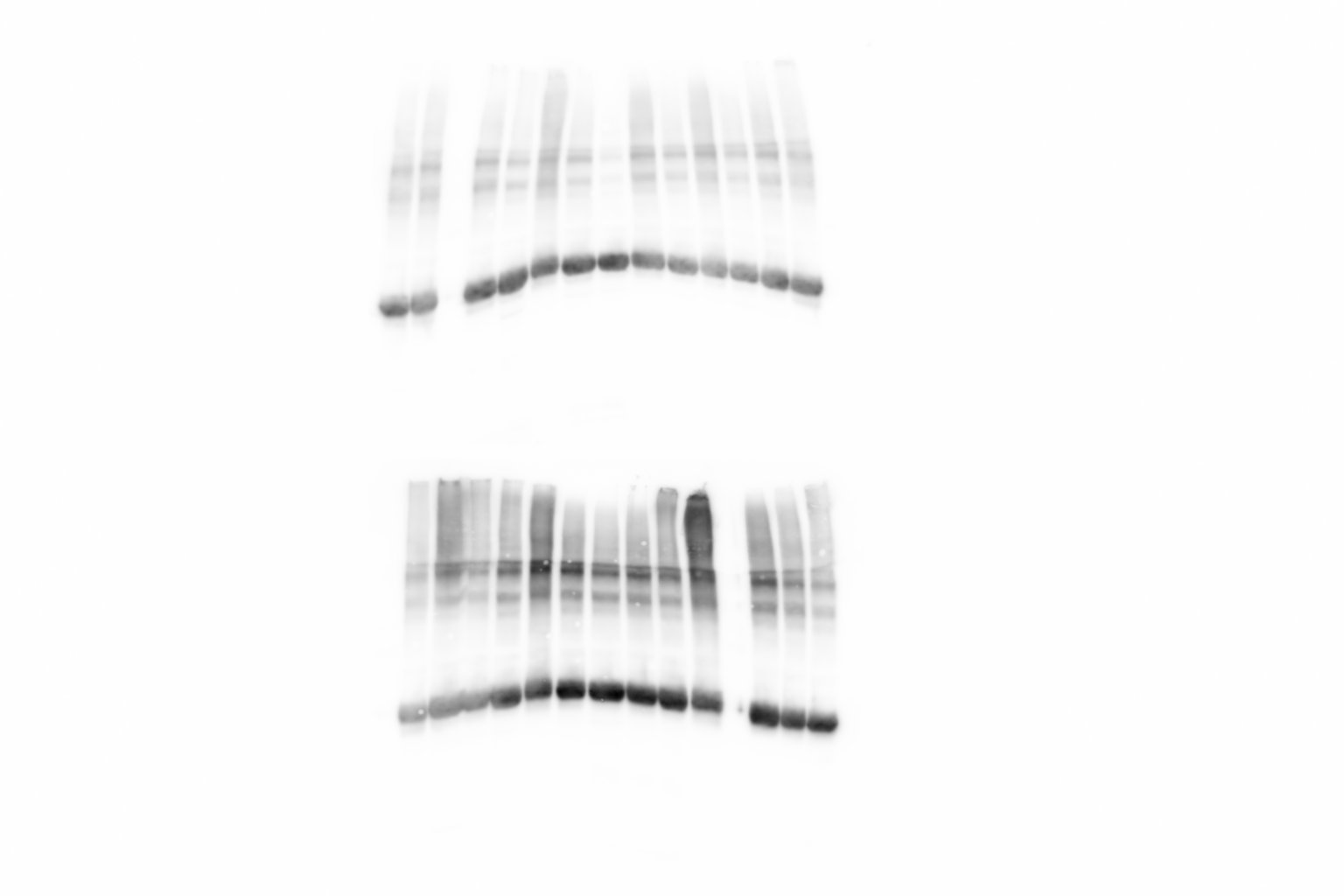

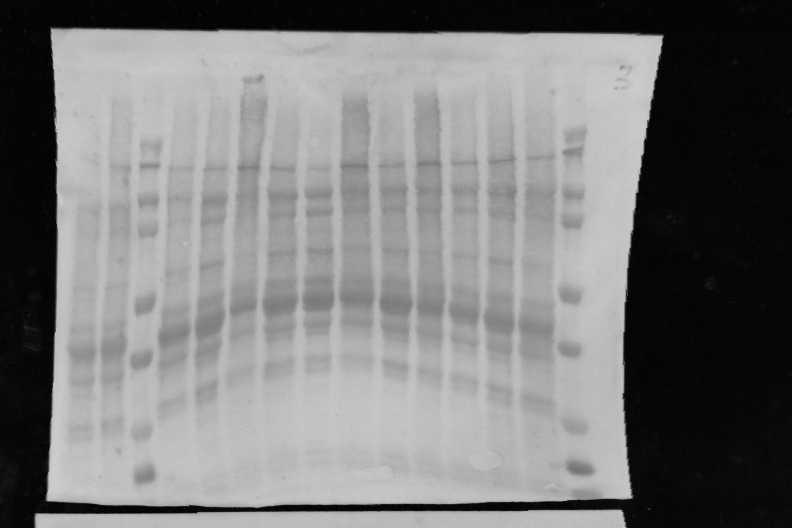

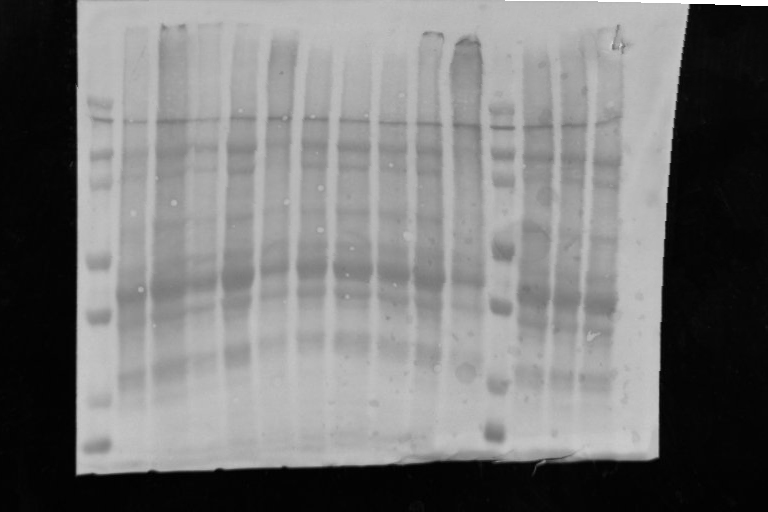


(D)


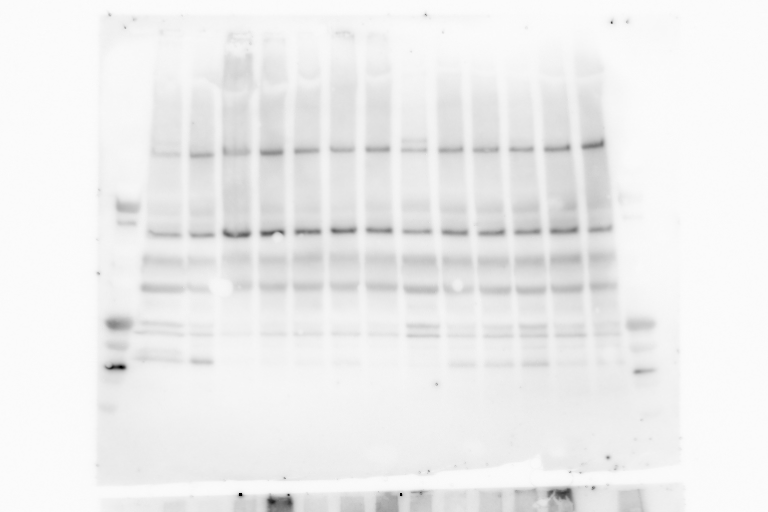

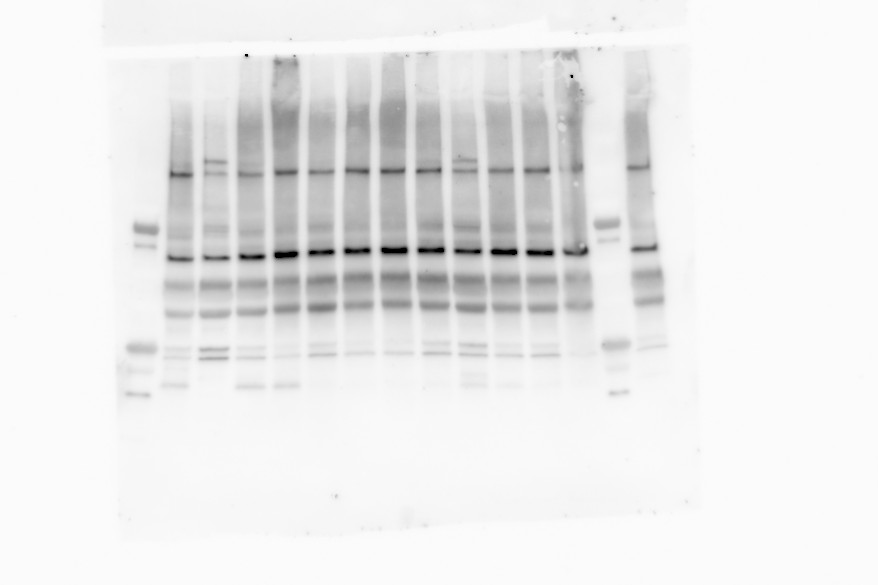

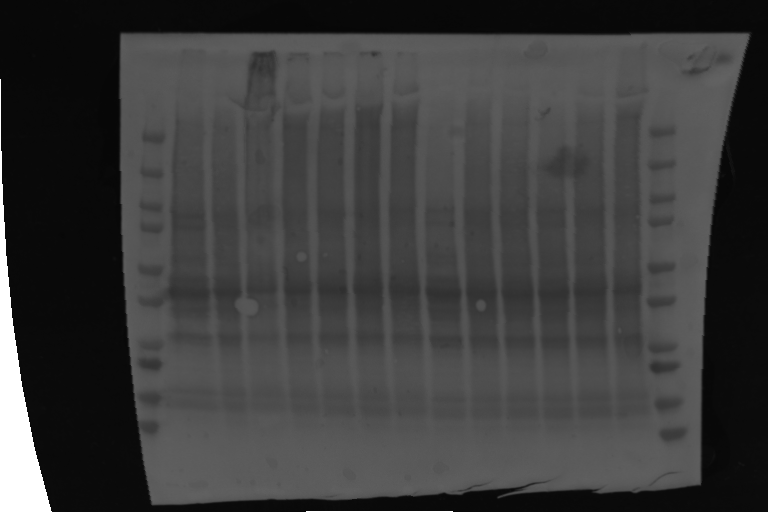

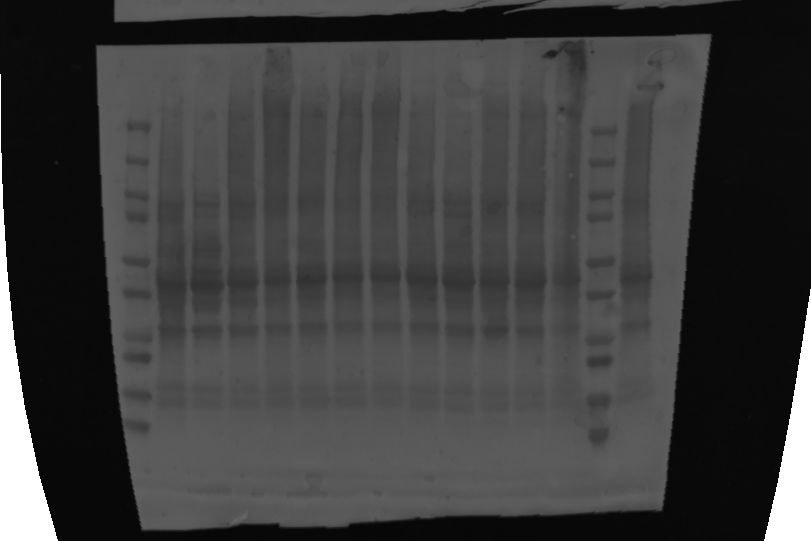


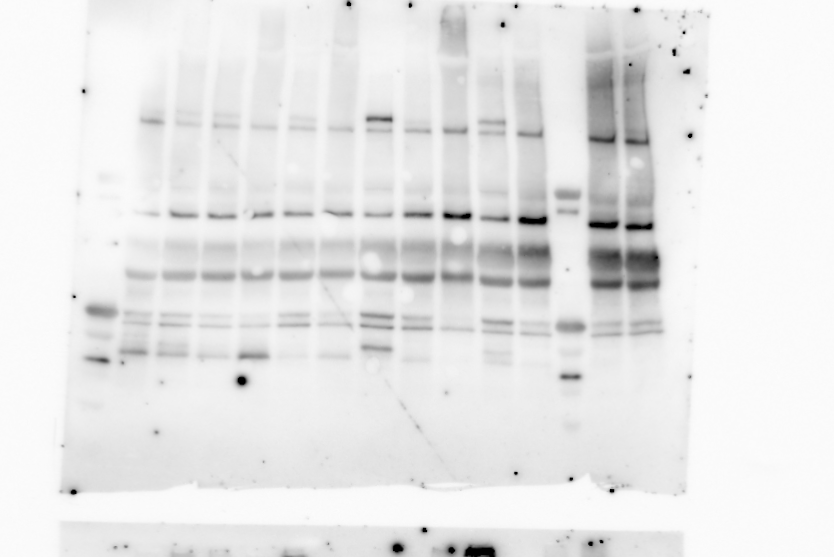

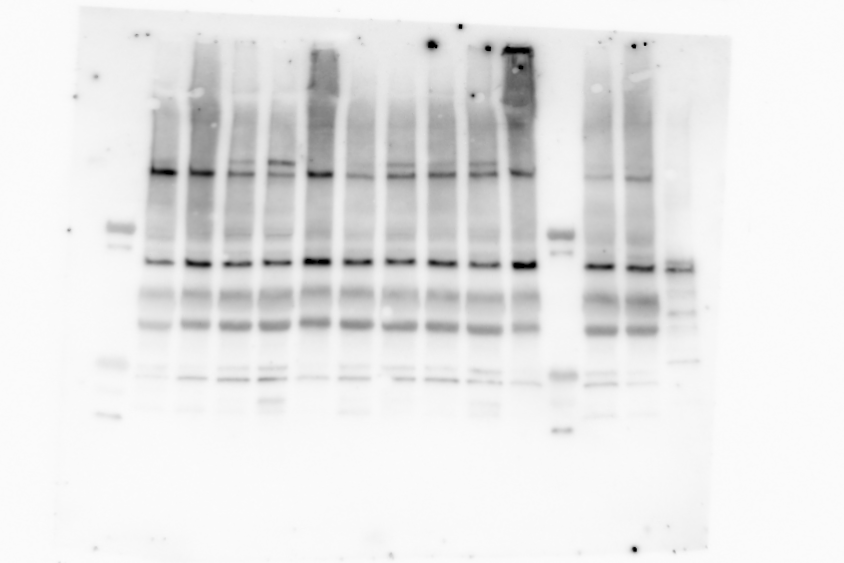

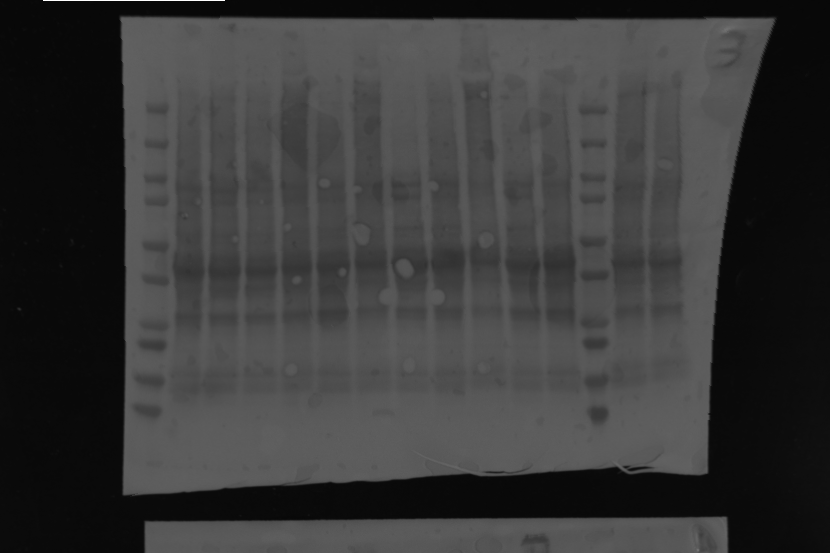

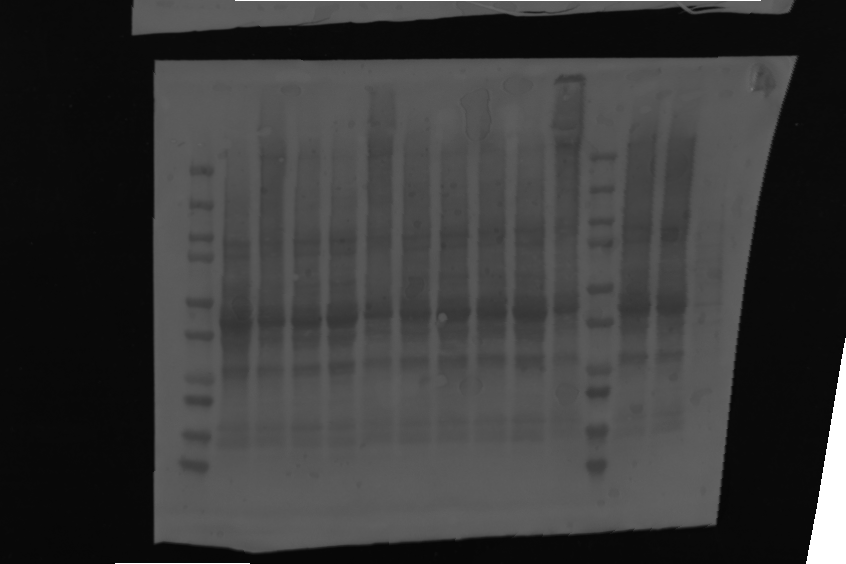


(E)


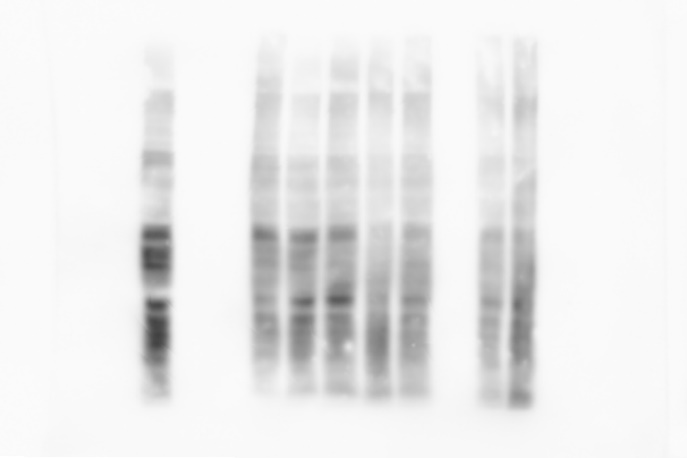

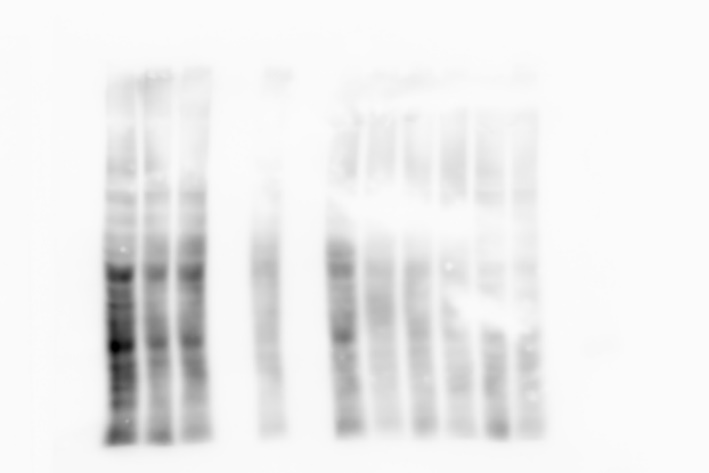

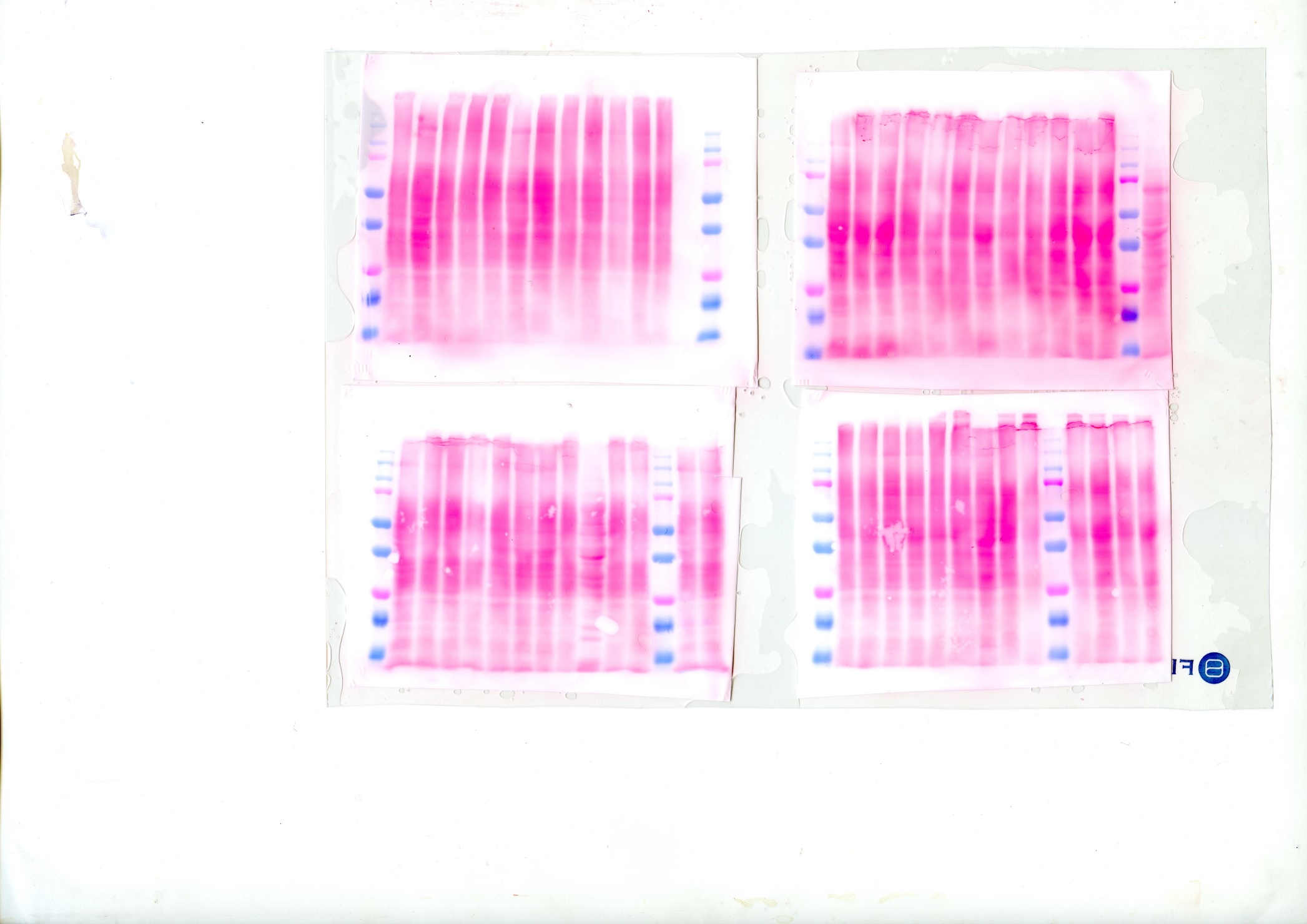

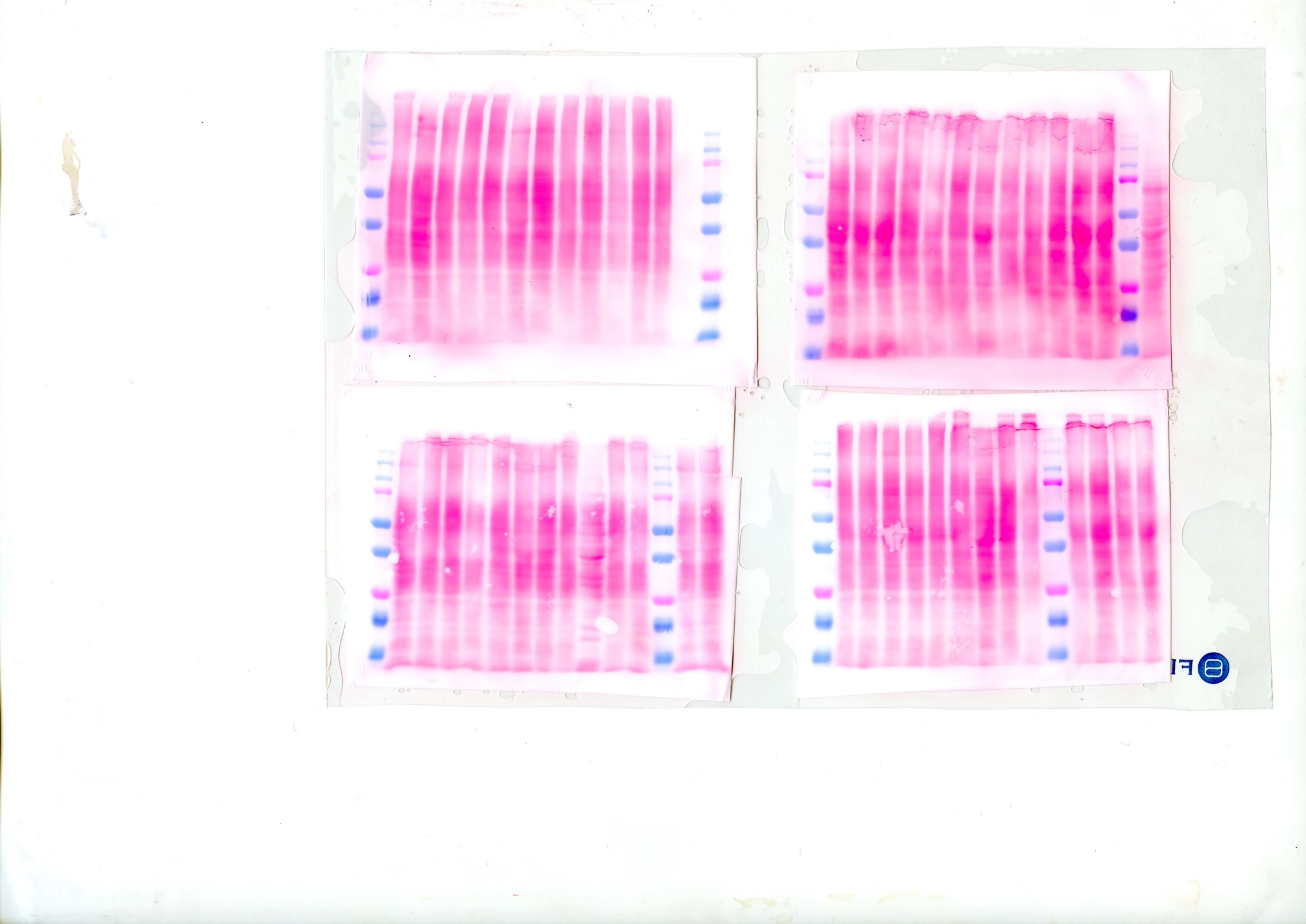


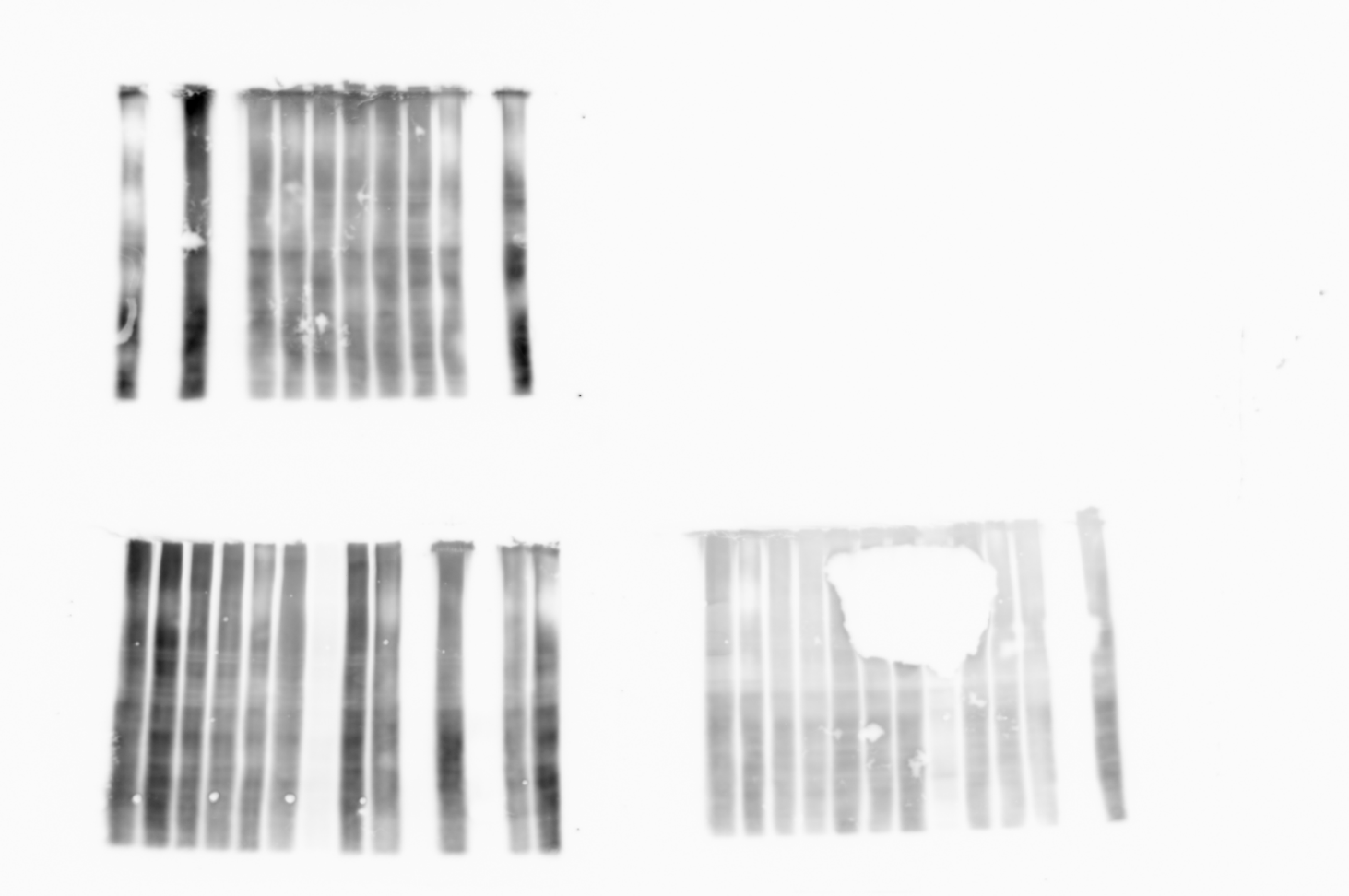

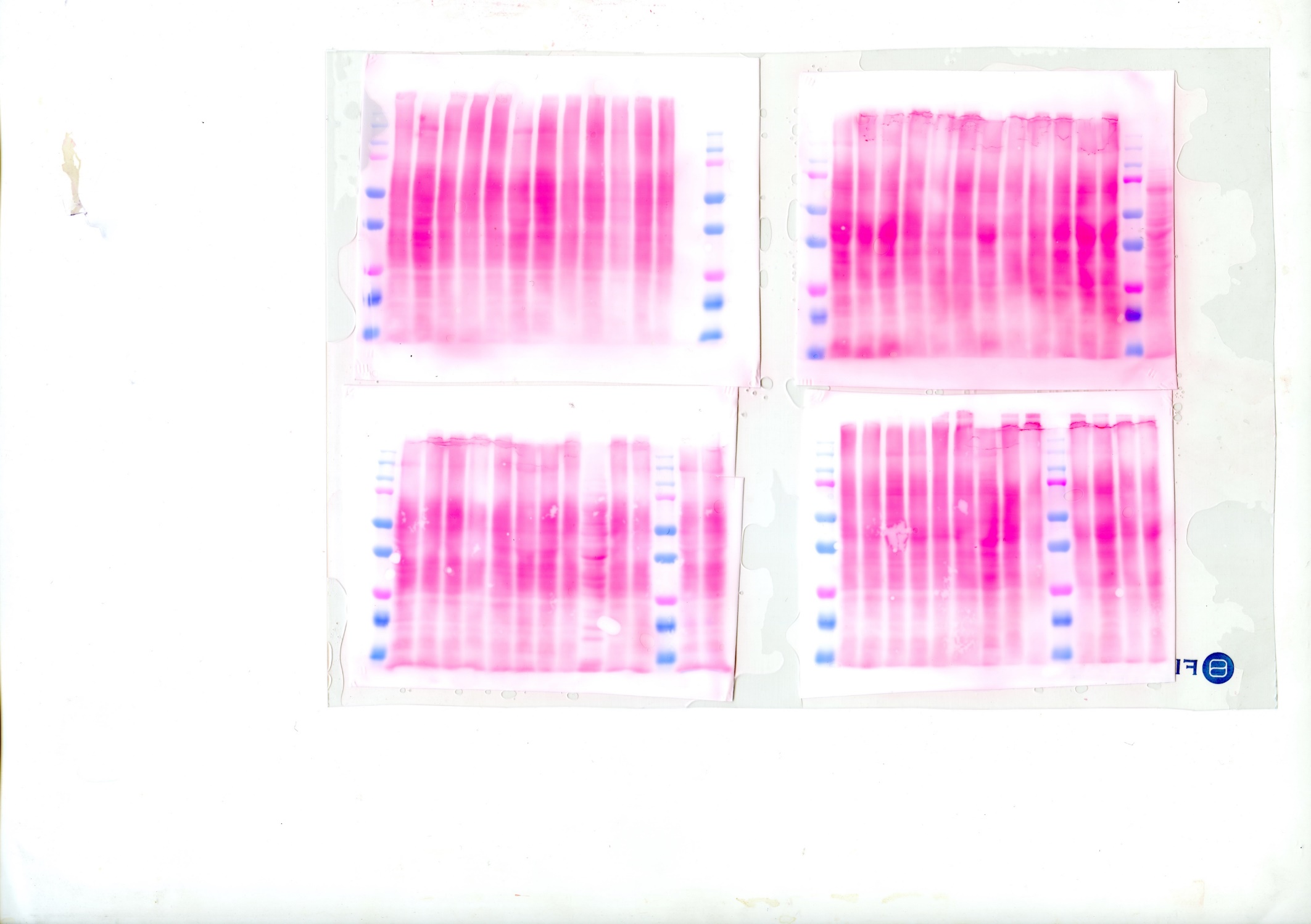


(F)


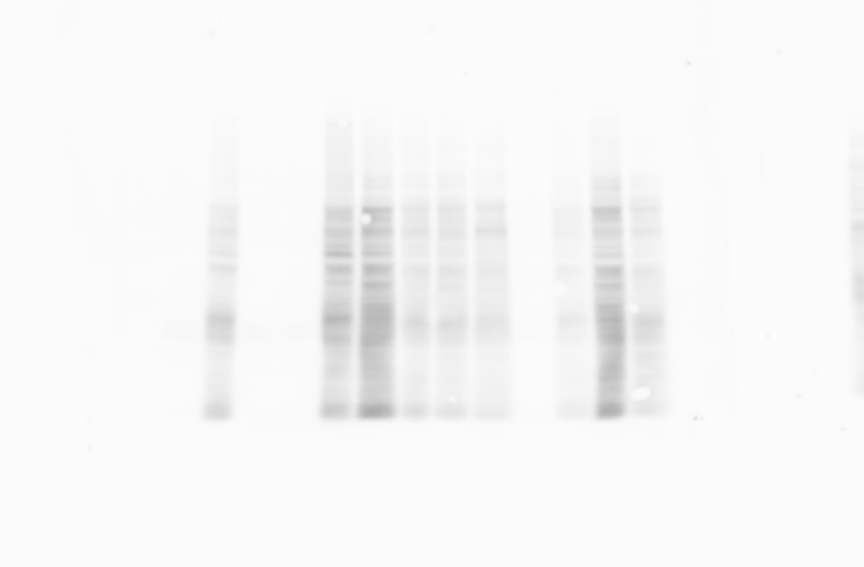

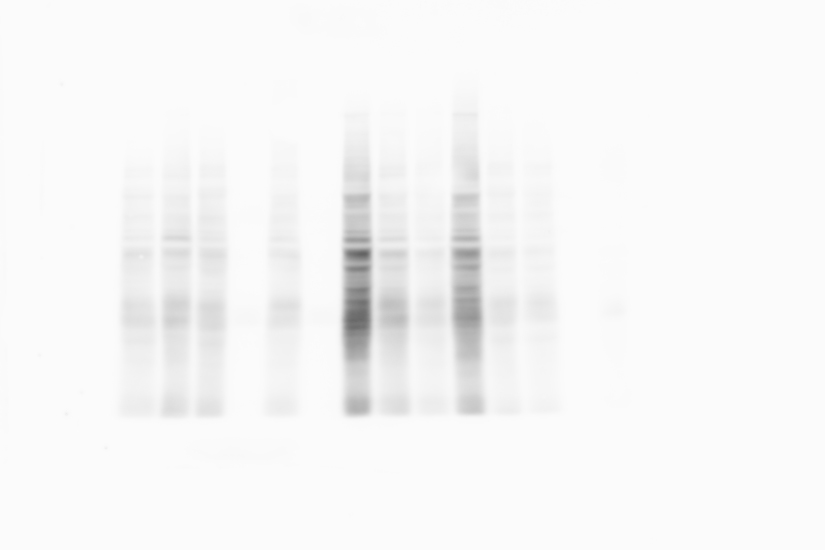

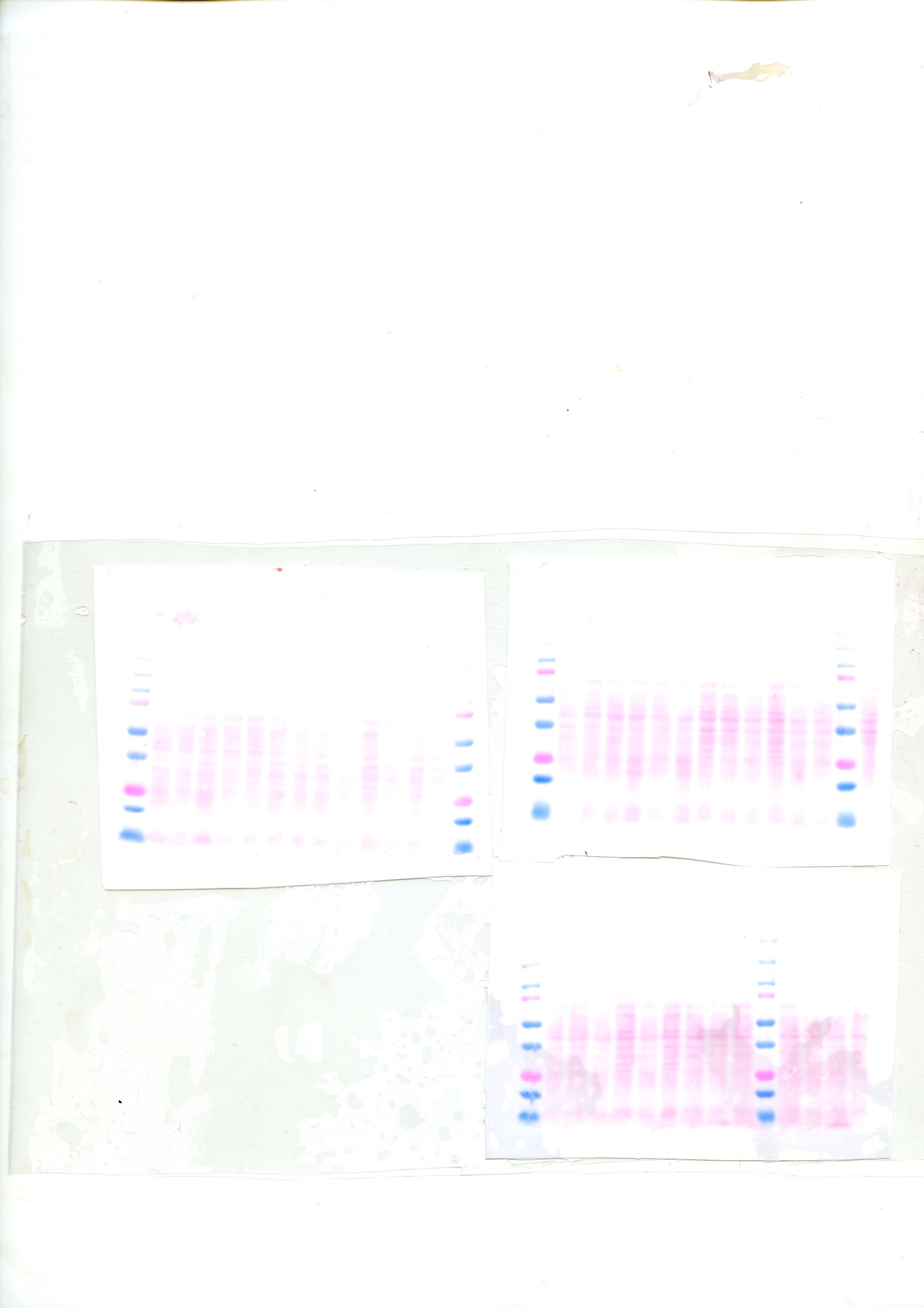

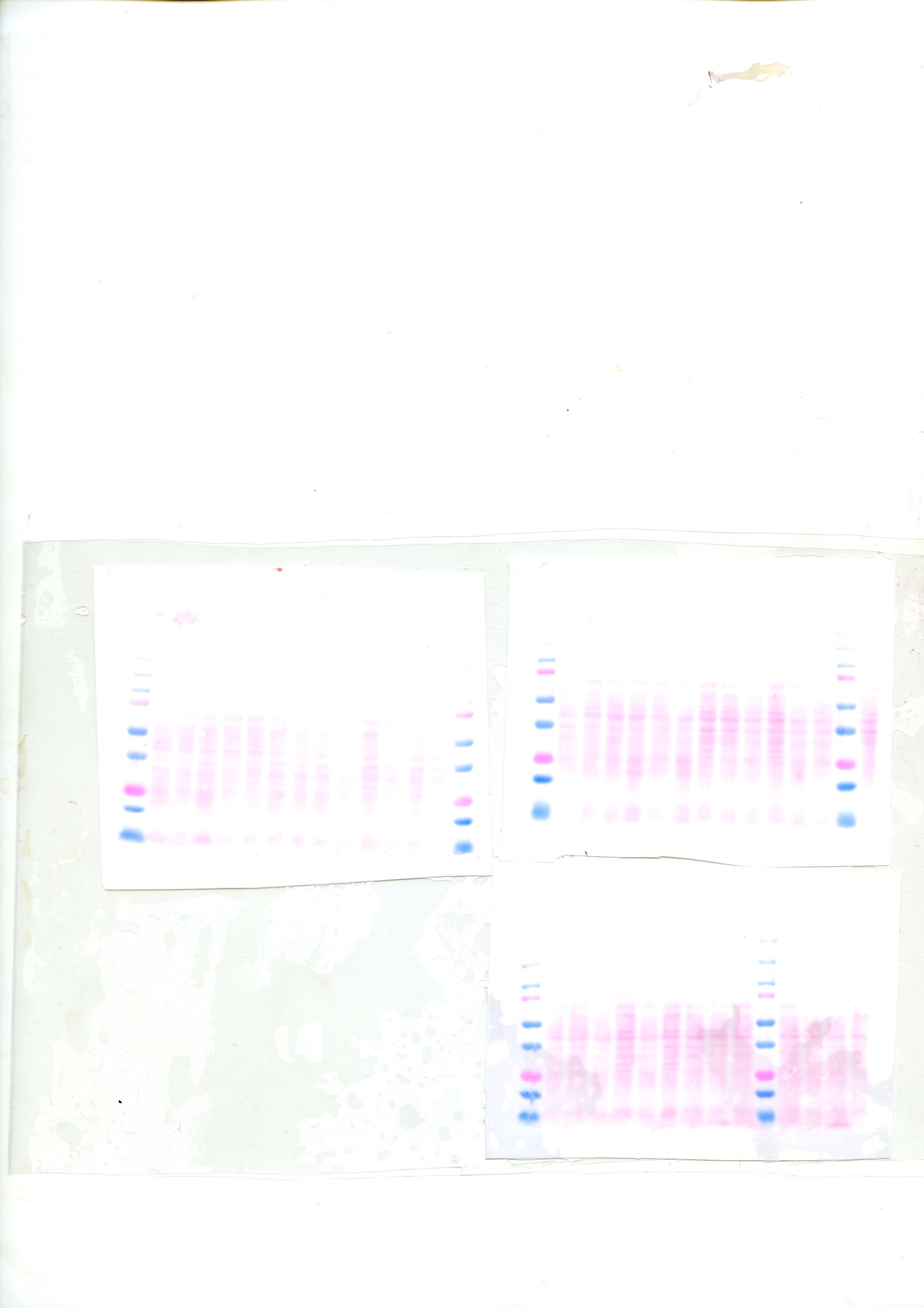


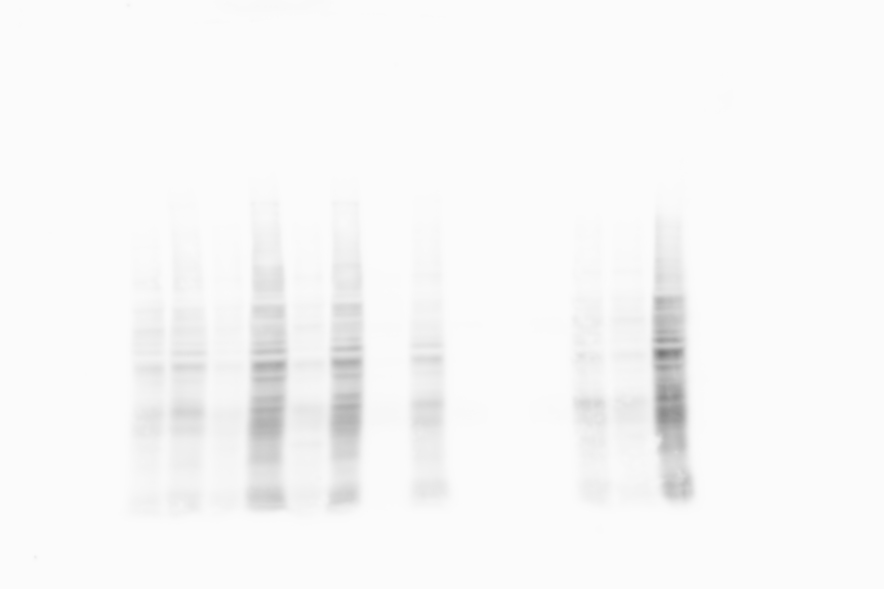


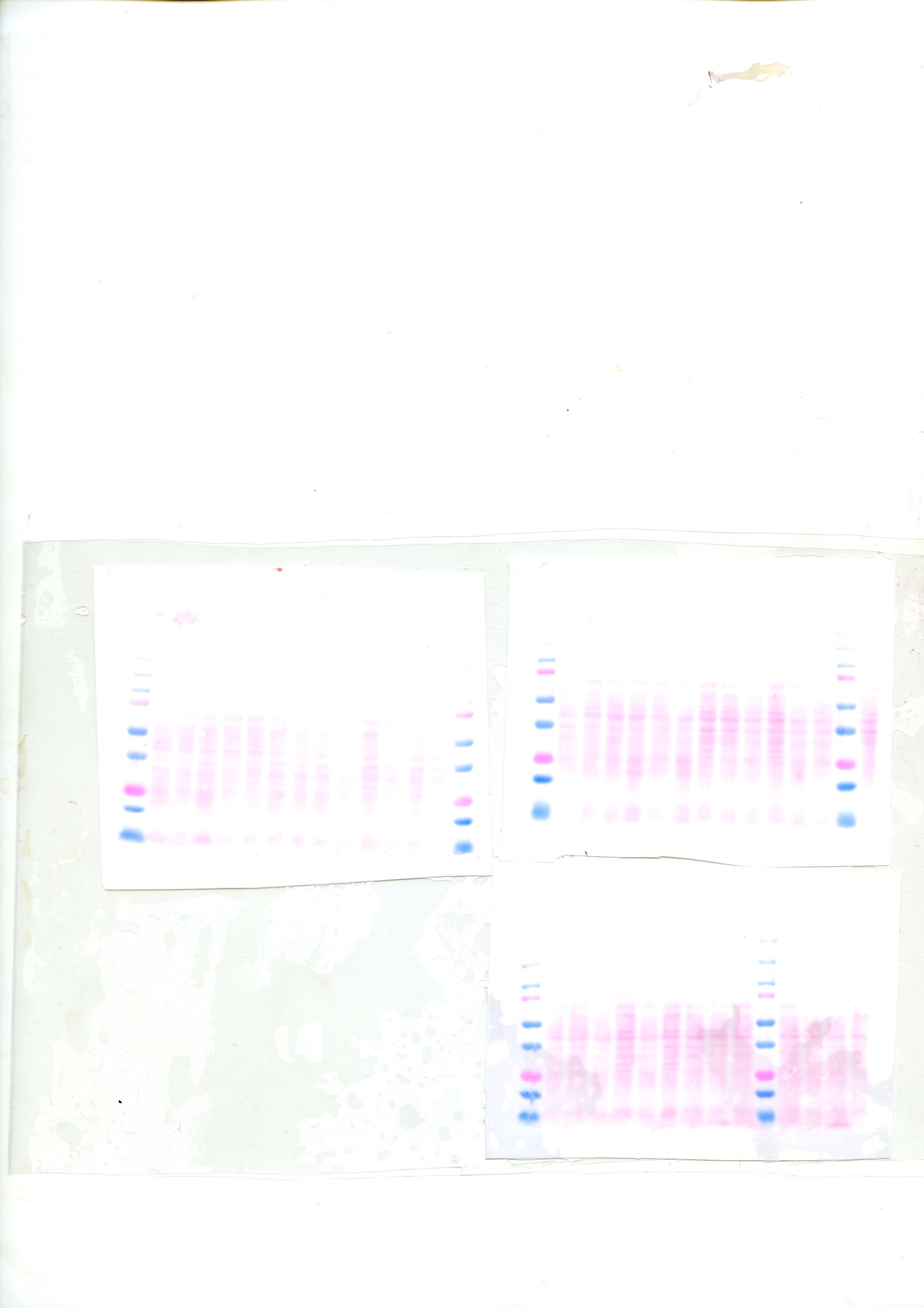


(G)


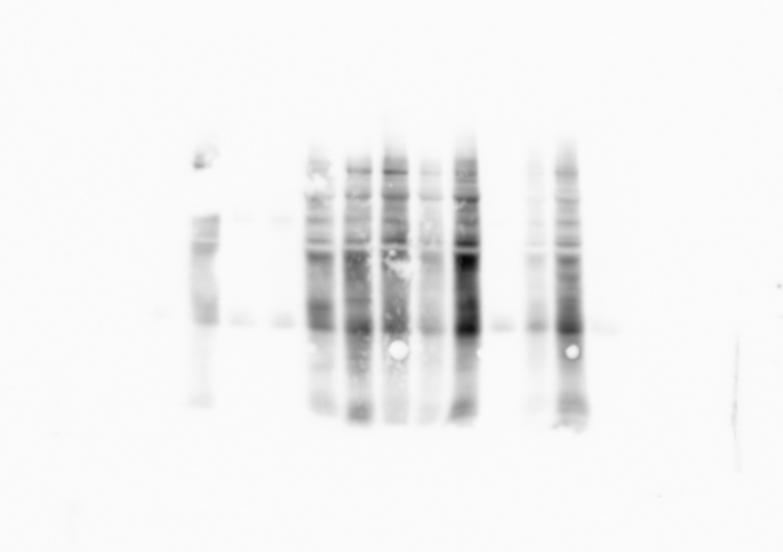

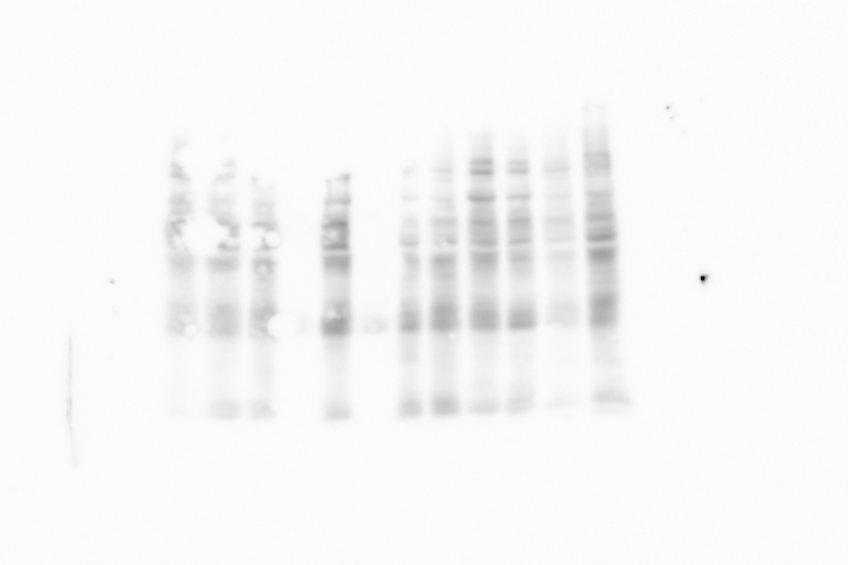

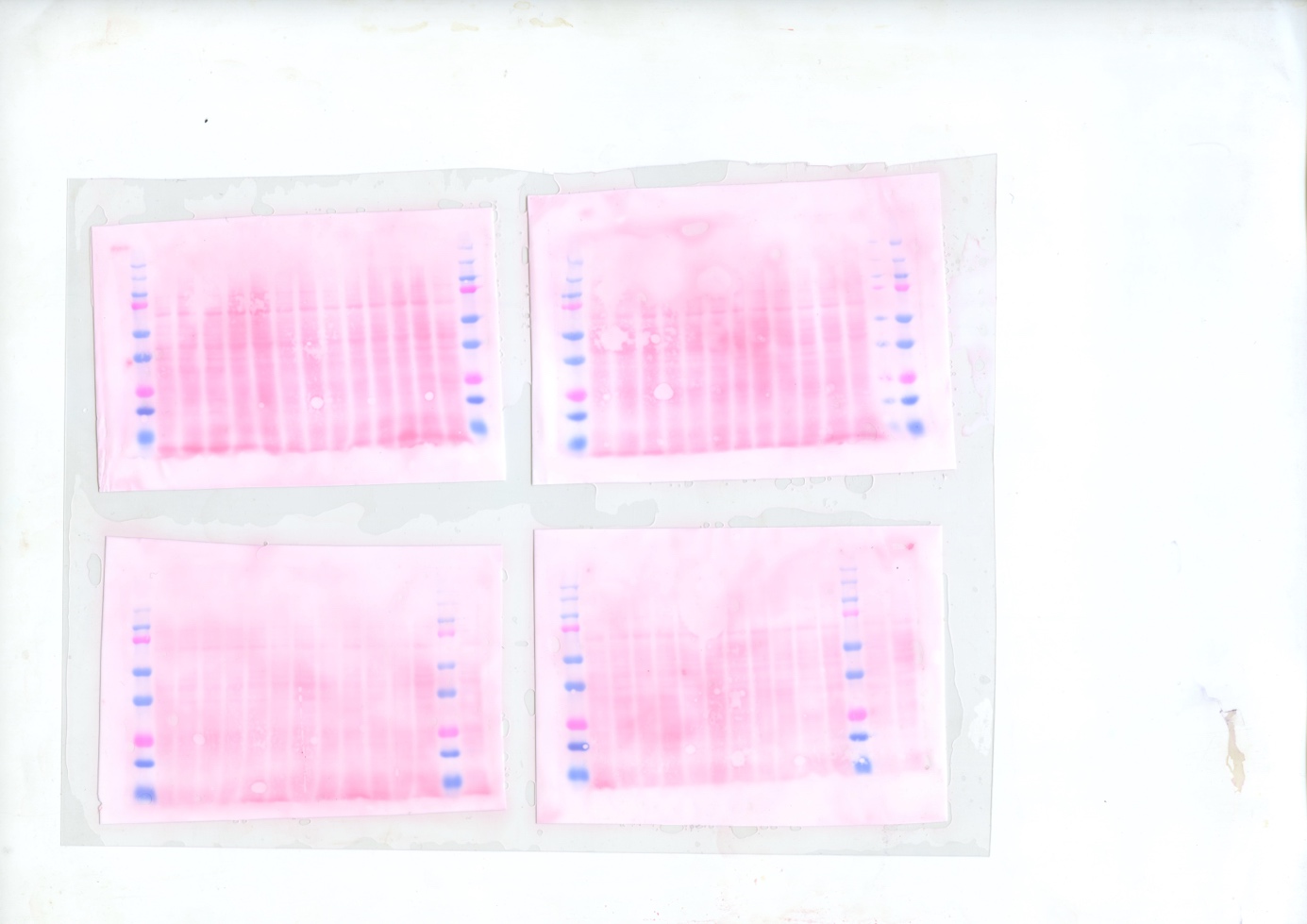

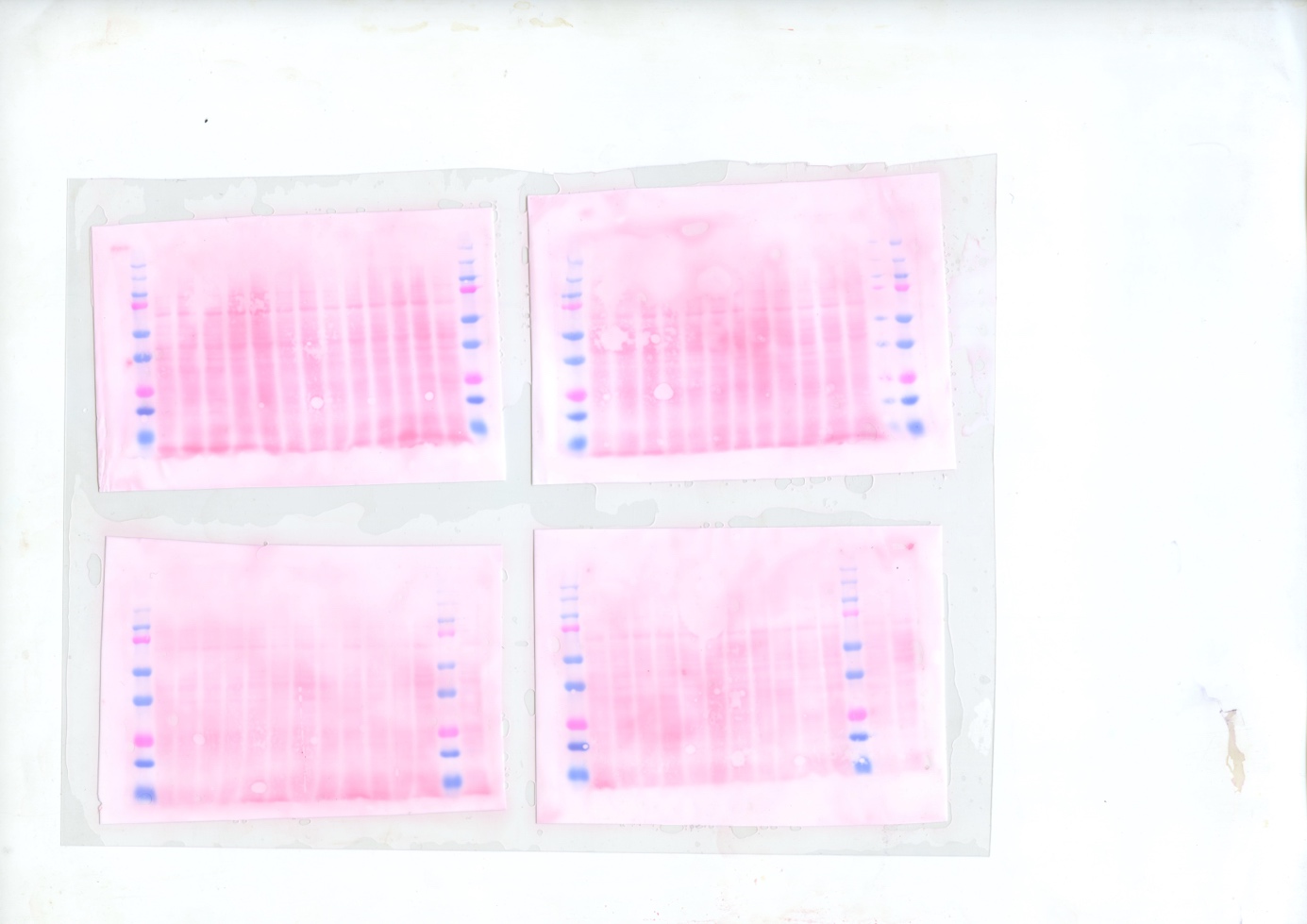


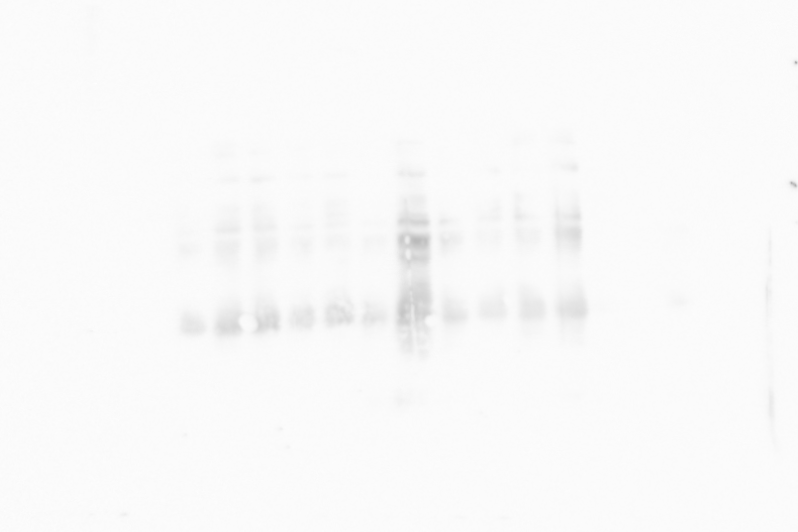

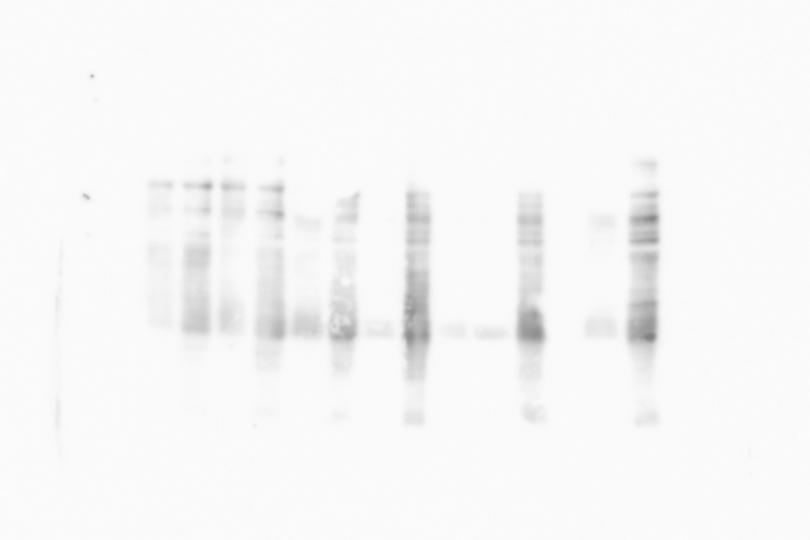


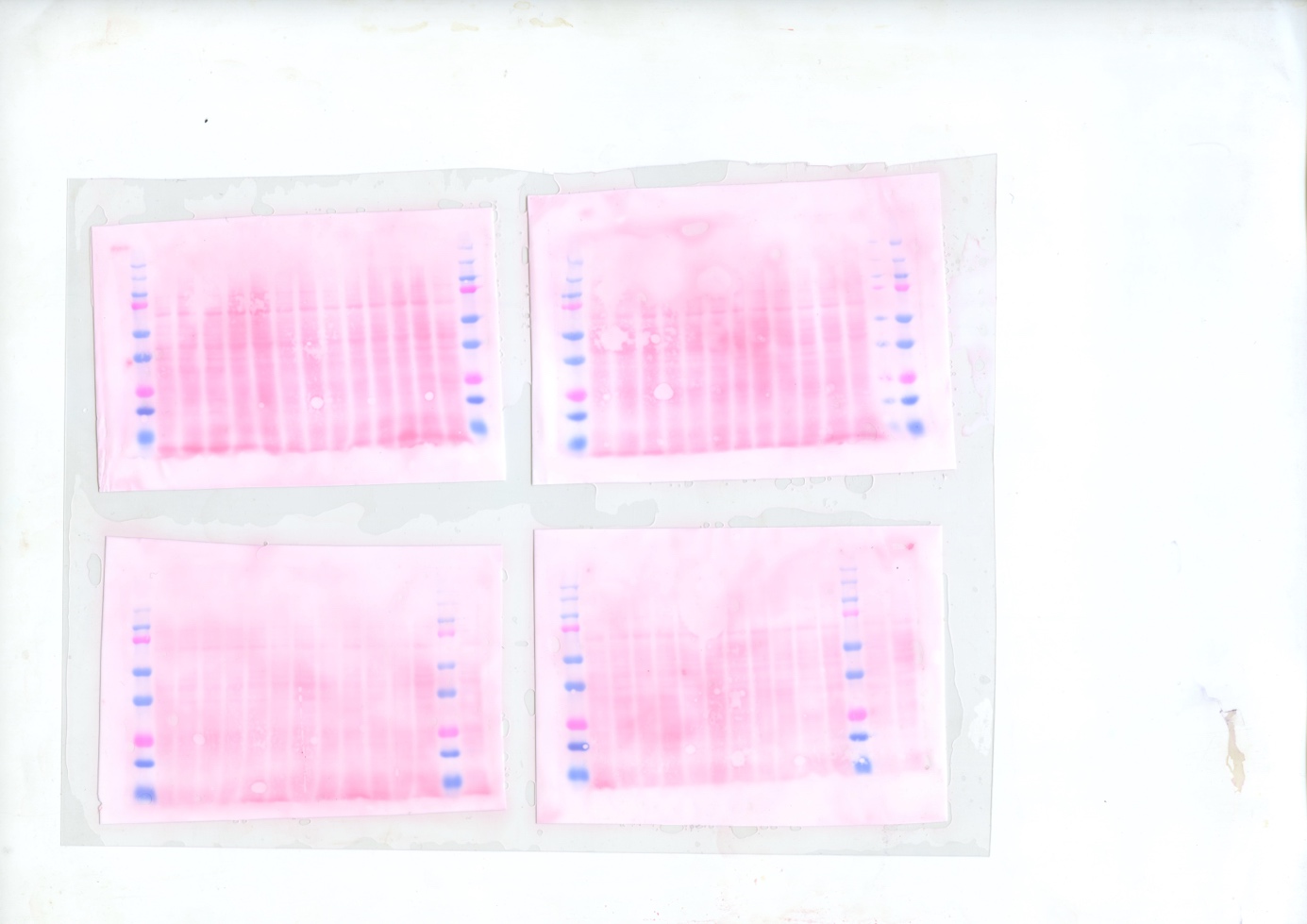

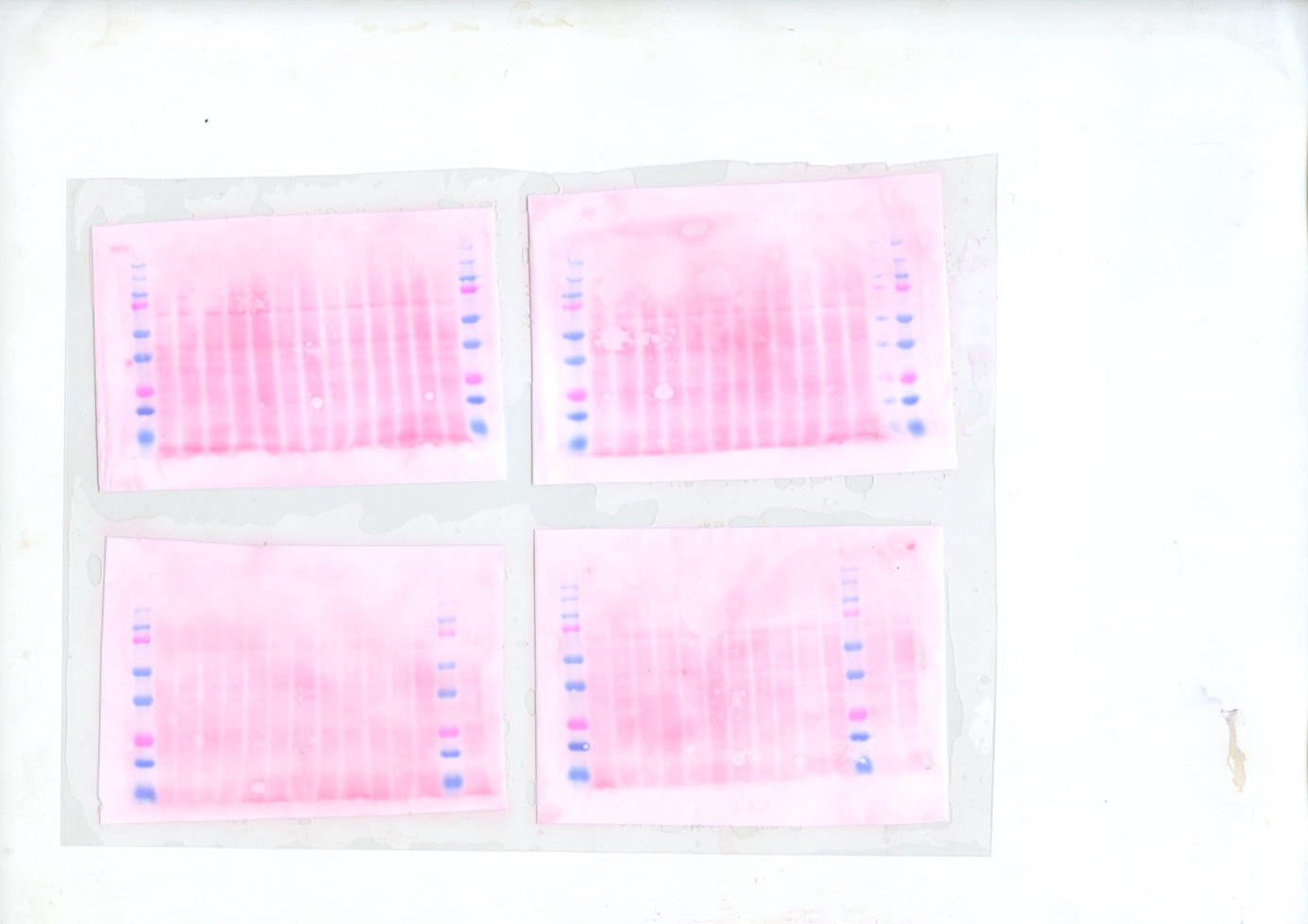


**Figure S1. Western blot images of tissue protein synthesis and muscle proteolysis**

Western blot images (A) Tibialis total protein synthesis, (B) Tibialis 4EBP1 phosphorylation on serine 65, (C) Tibialis Atrogin1 expression, (D) Tibialis Murf1 expression, (E) Liver total protein synthesis, (F) Jejunal mucosa total protein synthesis and (G) Ileal mucosa total protein synthesis. (A) (C) (D) (E) (F) (G) Western Blot images of puromycin followed by ponceau membrane, (B) Western Blot images of 4EBP1 phosphorylation on serine 65 followed by total form of 4E-BP1.
