# Supplementary material for: Protein intake in cancer: Does it improve nutritional status and/or modify tumour response to chemotherapy?
Source: J Cachexia Sarcopenia Muscle. 2023 Sep 4;14(5):2003–15. doi: 10.1002/jcsm.13276 (PMC10570073; doi:10.1002/jcsm.13276)
Supplement: Supplementary file 2 — Figure S2. Western blot images of tumor protein synthesis. Western blot images of puromycin followed by ponceau membrane. [file JCSM-14-2003-s002.docx]

**TUMEUR synthèse protéique**


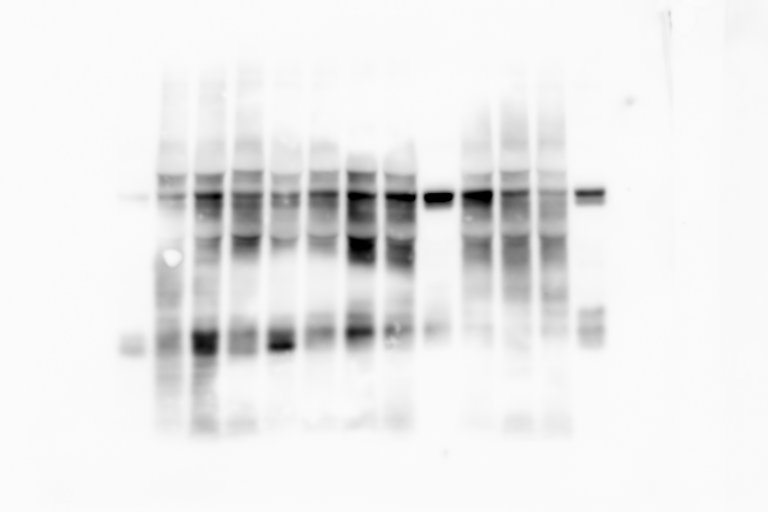

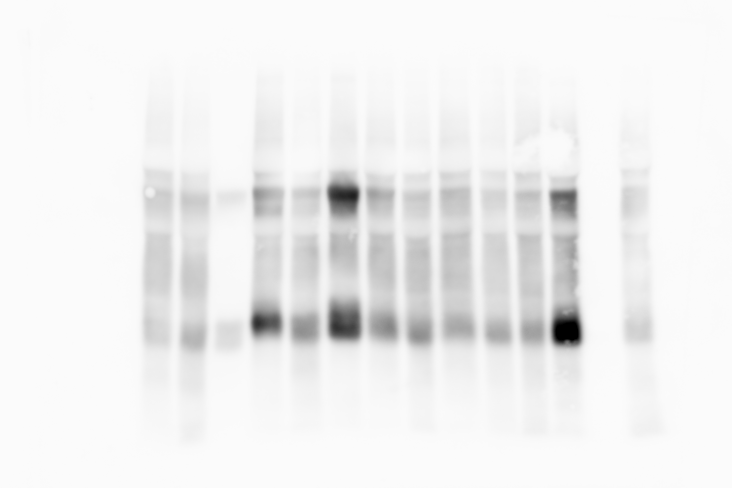


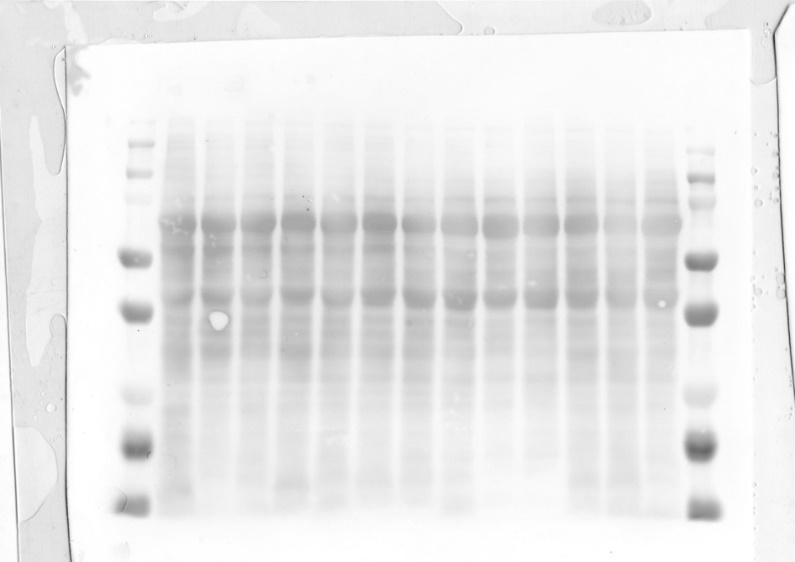

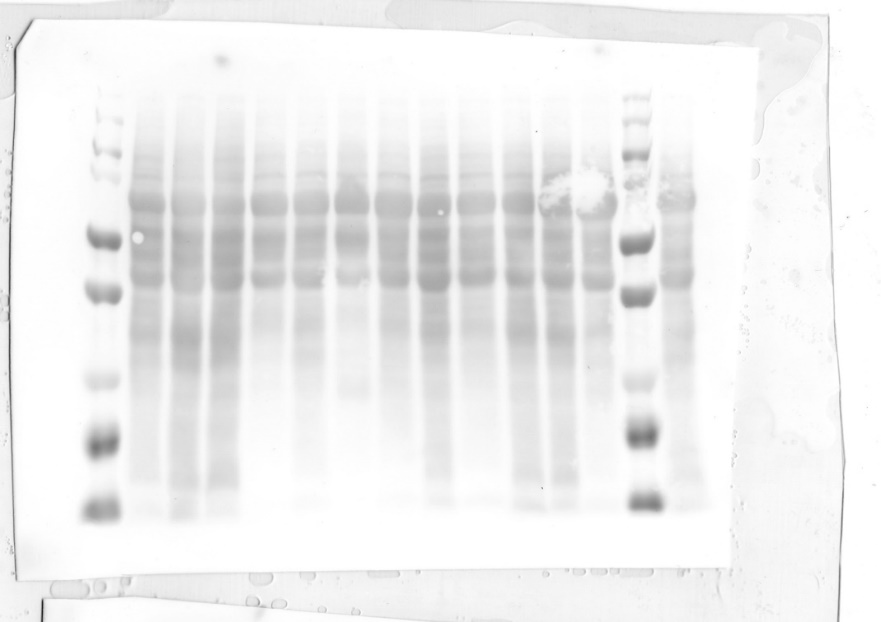


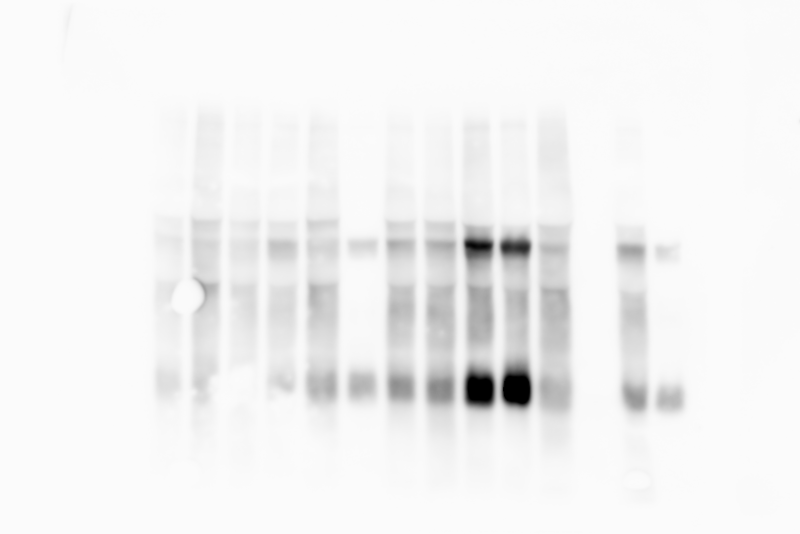

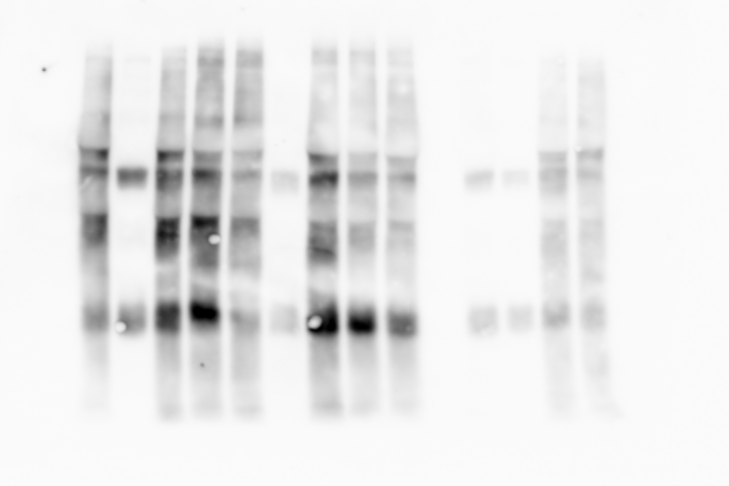

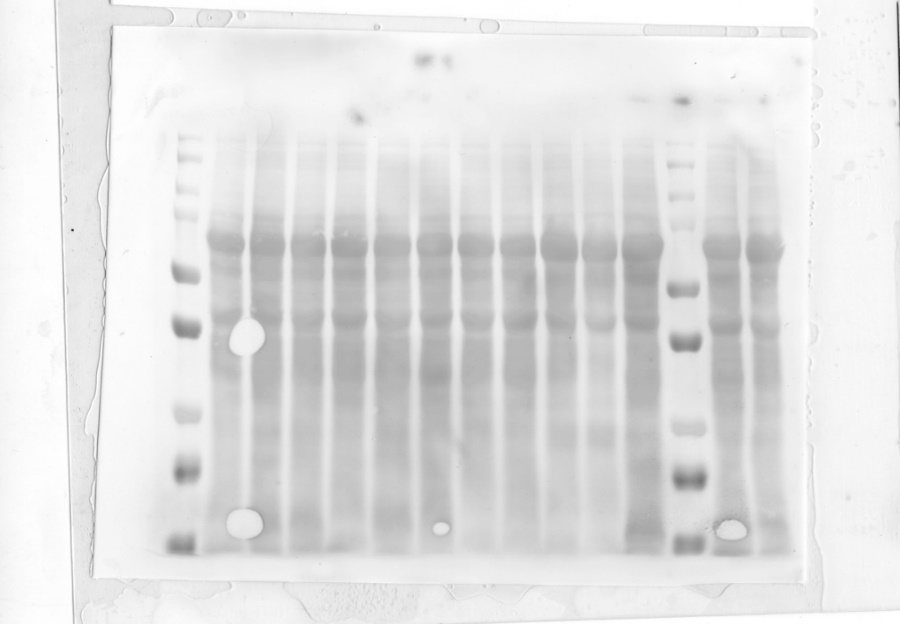

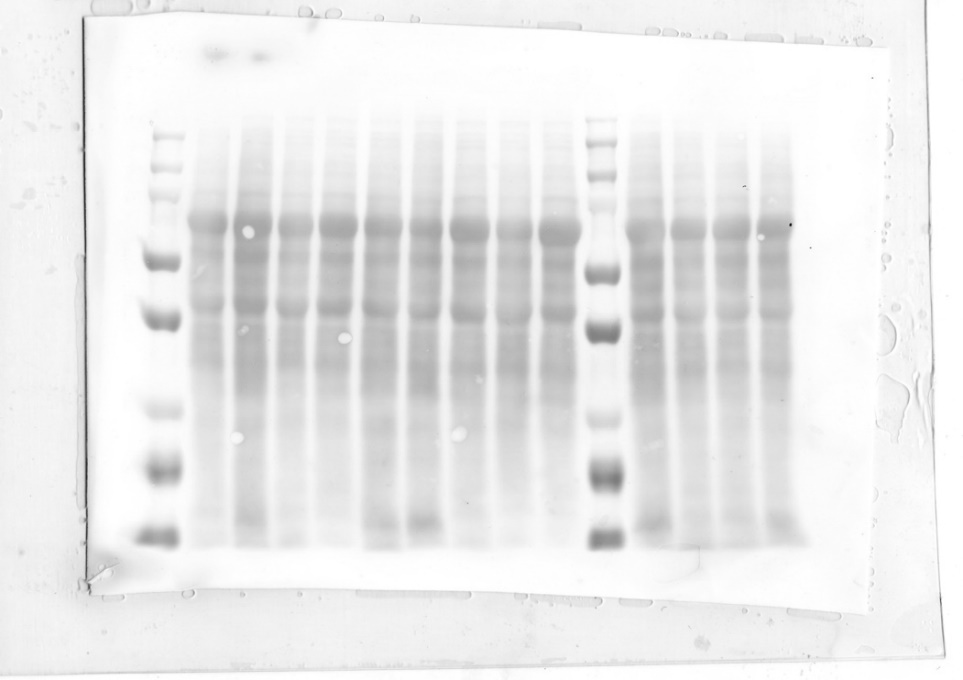


**Figure S2. Western blot images of tumor protein synthesis**

Western blot images of puromycin followed by ponceau membrane.
