# Supplementary material for: Protein intake in cancer: Does it improve nutritional status and/or modify tumour response to chemotherapy?
Source: J Cachexia Sarcopenia Muscle. 2023 Sep 4;14(5):2003–15. doi: 10.1002/jcsm.13276 (PMC10570073; doi:10.1002/jcsm.13276)
Supplement: Supplementary file 3 — Figure S3. Immune exploration in blood [file JCSM-14-2003-s003.docx]

**Supplementary data** Immune exploration in blood

(c)

(b)

(a)


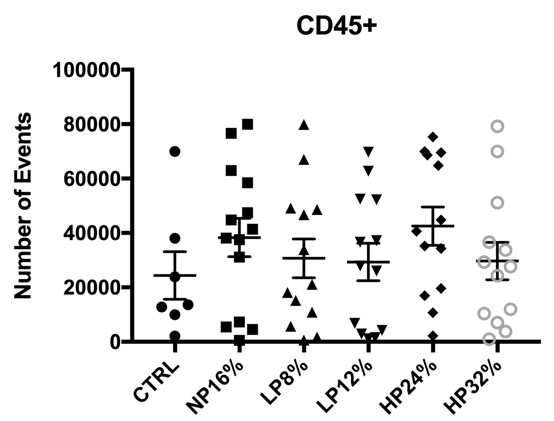

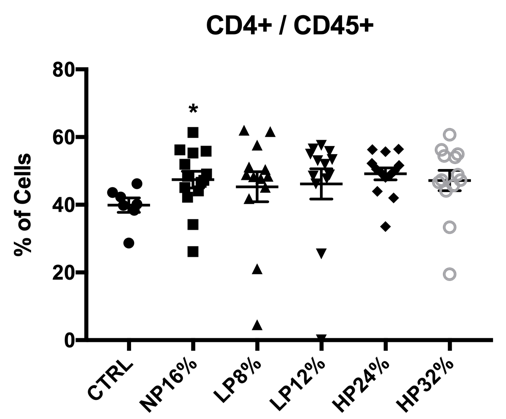

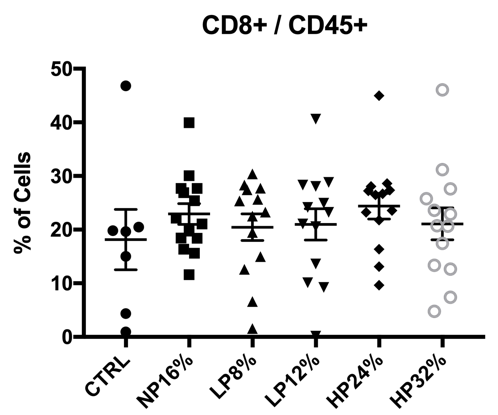


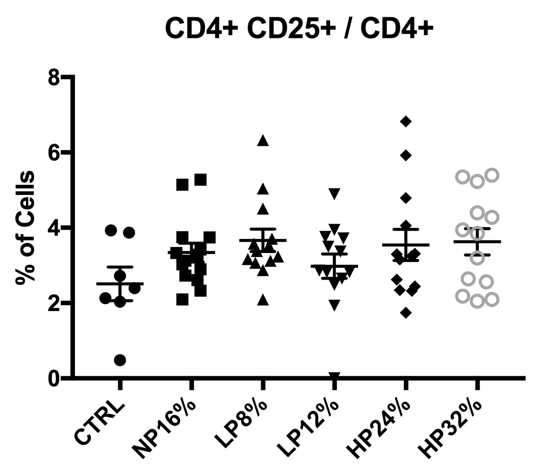

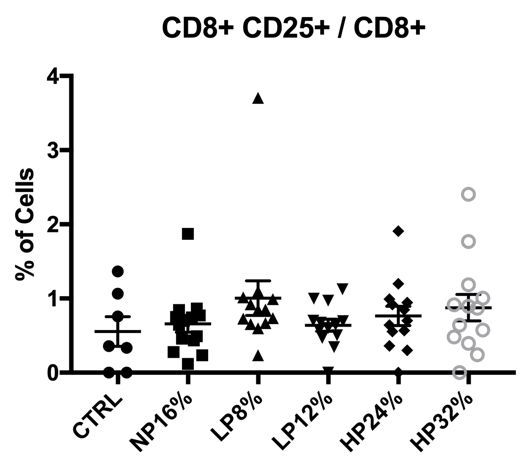

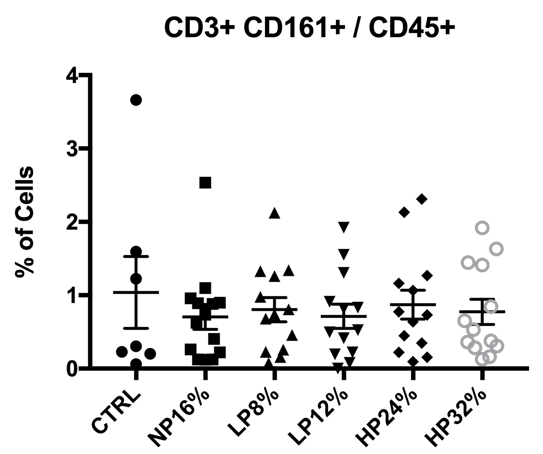


(f)

(e)

(d)


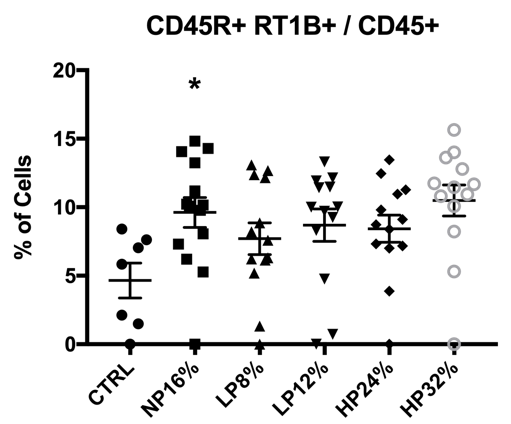

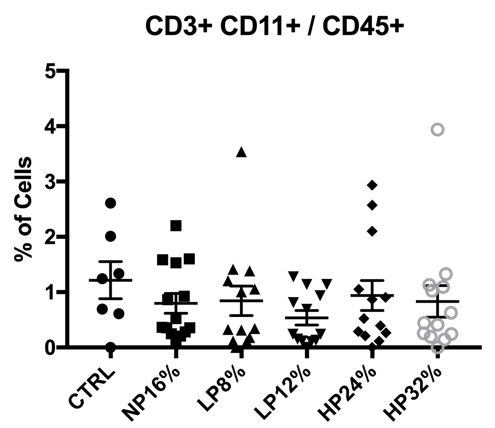

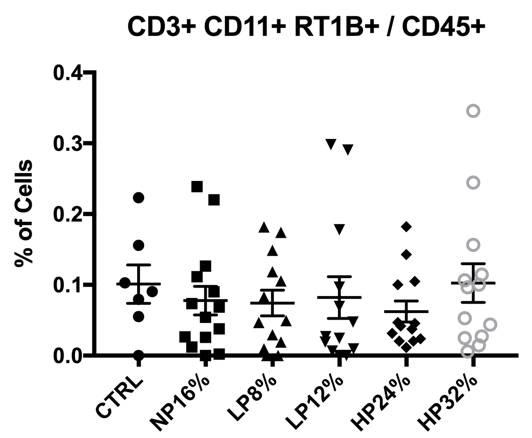


(i)

(h)

(g)


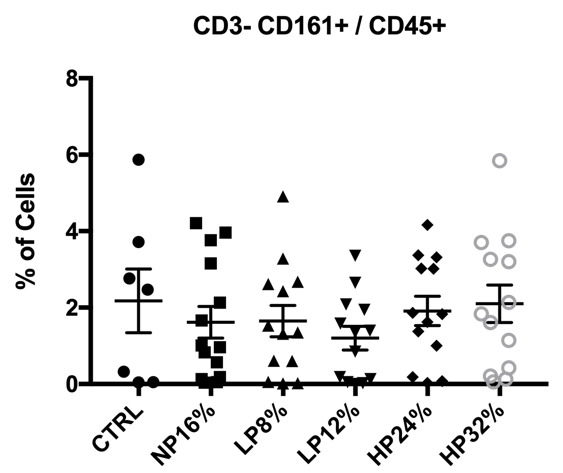


(j)

Number of events for total leukocytes and percentage of cells for other figures of immune cells identified in blood (*Table* S1). (a) CD45+ leukocyte cells; (b) CD4+ T-cells; (c) CD8+ T-cells; (d) CD4+ regulatory T-cells; (e) CD8+ regulatory T-cells; (f) CD3+ CD161+ Natural killer T-cells; (g)CD45R+RT1B+ B-cells; (h) CD3+ CD11+ monocytes; (i)CD3+ CD11+ RT1B+ dendritic cells; (j) CD3- CD161+ Natural killer cells. Scatterplots showing mean ± SEM. * is significantly different at p = 0.05.

(a) (b) (c) (d) (e) (f) (g) (h) (i) (j) CTRL, n=7; NP16%, n=14; LP8%, n=13; LP12%, n=13; HP24%, n=13; HP32%, n=13.

**Supplementary data** Immune exploration in spleen

(a)


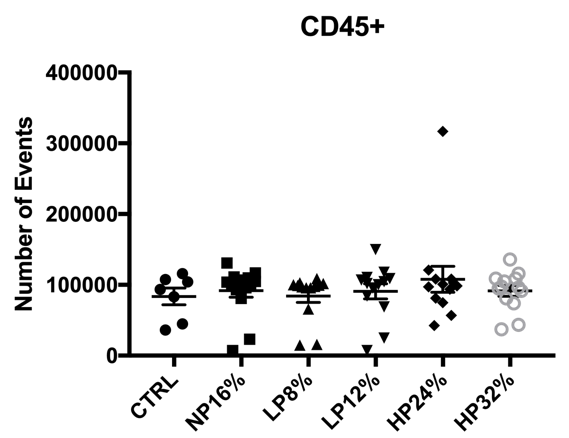

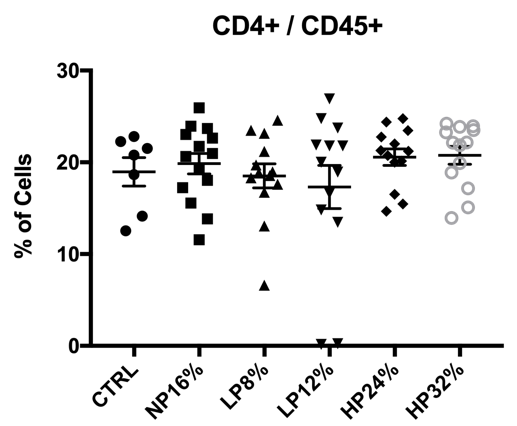

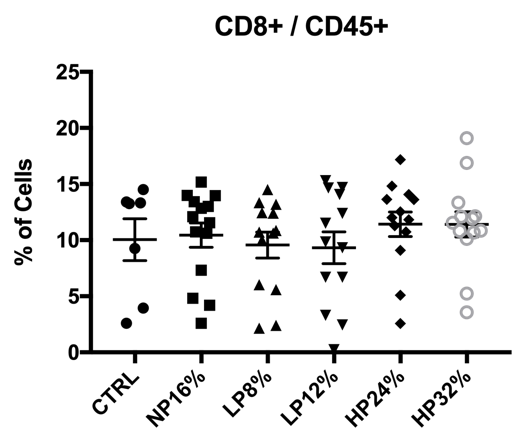


(c)

(b)


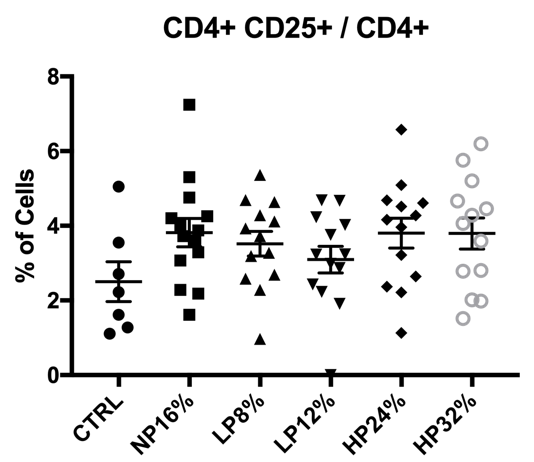

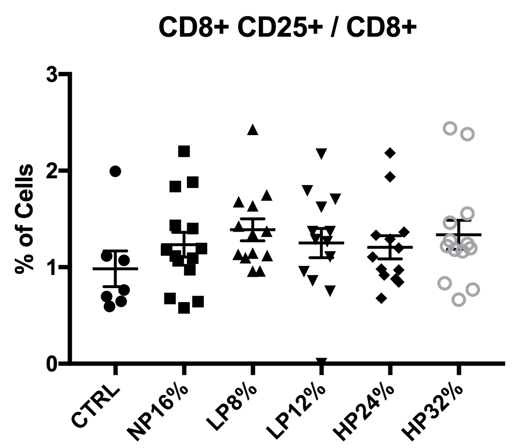

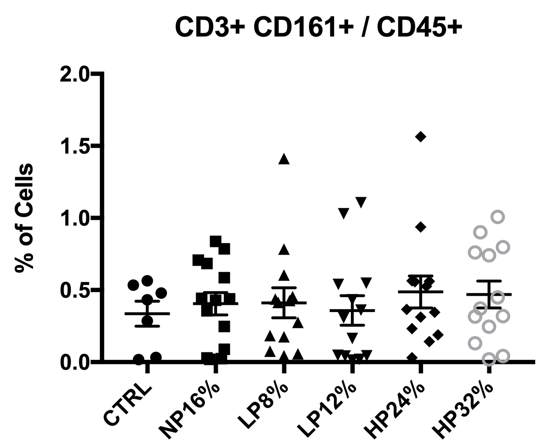


(d)

(e)

(f)

(g)

(h)

(i)


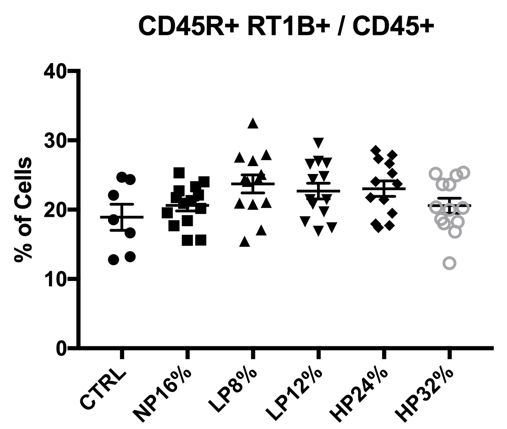

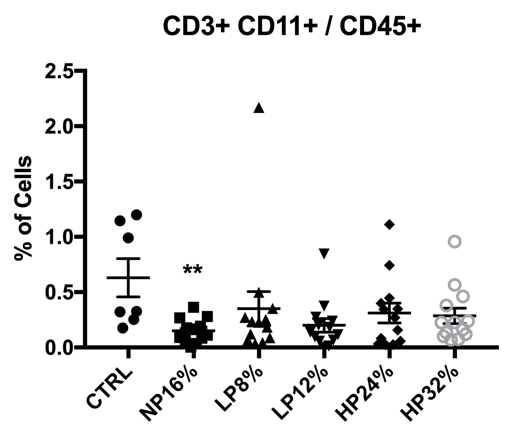

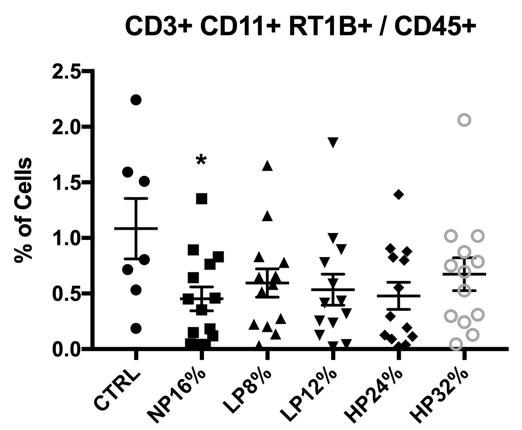


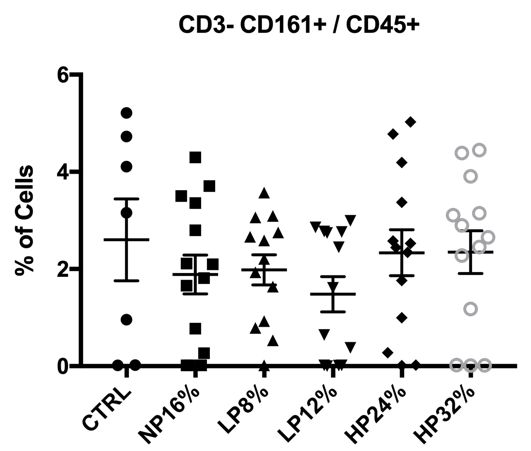


(j)

Number of events for total leukocytes and percentage of cells for other figures of immune cells identified in spleen (*Table* S1). (a) CD45+ leukocyte cells; (b) CD4+ T-cells; (c) CD8+ T-cells; (d) CD4+ regulatory T-cells; (e) CD8+ regulatory T-cells; (f) CD3+ CD161+ Natural killer T-cells; (g)CD45R+RT1B+ B-cells; (h) CD3+ CD11+ monocytes; (i)CD3+ CD11+ RT1B+ dendritic cells; (j) CD3- CD161+ Natural killer cells. Scatterplots showing mean ± SEM. * is significantly different at p = 0.05.

(a) (b) (c) (d) (e) (f) (g) (h) (i) (j) CTRL, n=7; NP16%, n=14; LP8%, n=13; LP12%, n=13; HP24%, n=13; HP32%, n=13.

**Supplementary data** Immune exploration in Peyer’s patches


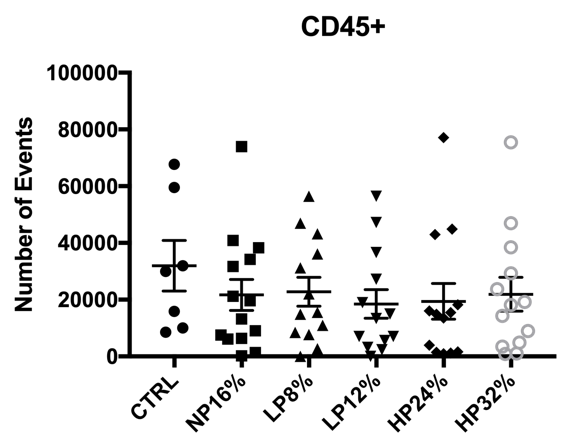

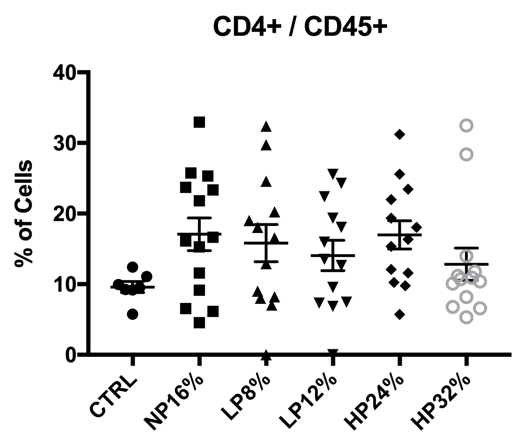

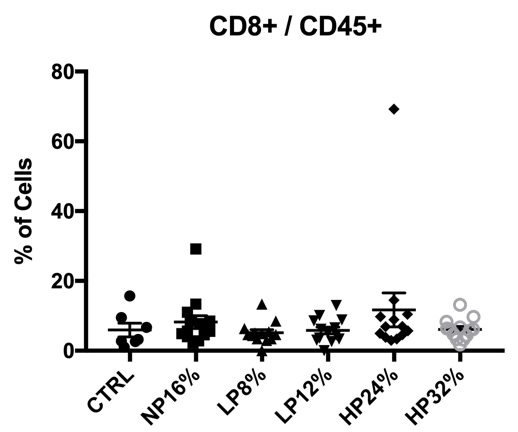


(f)

(e)

(d)

(c)

(b)

(a)


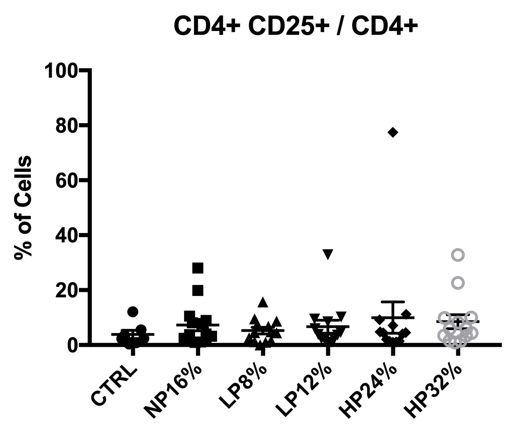

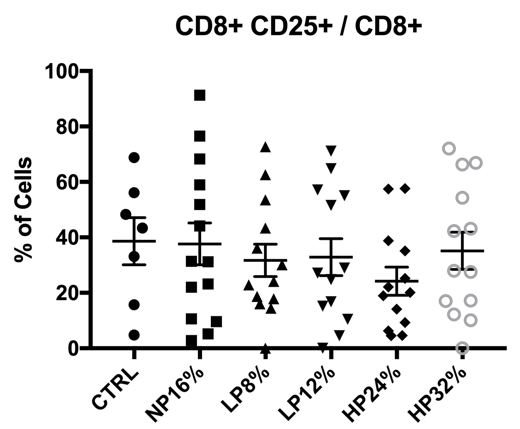

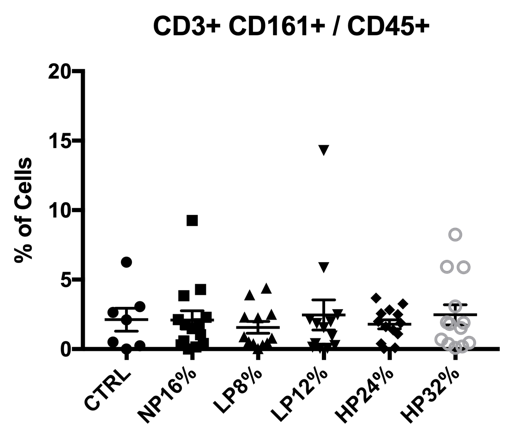


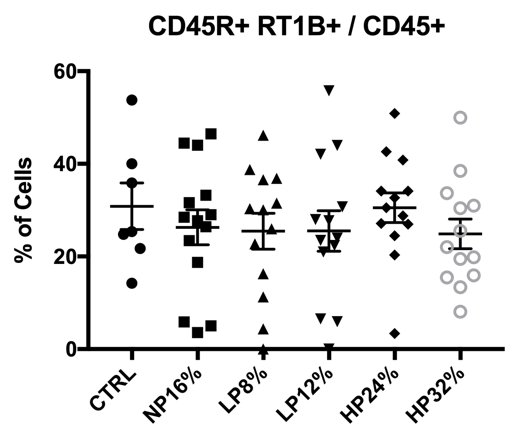

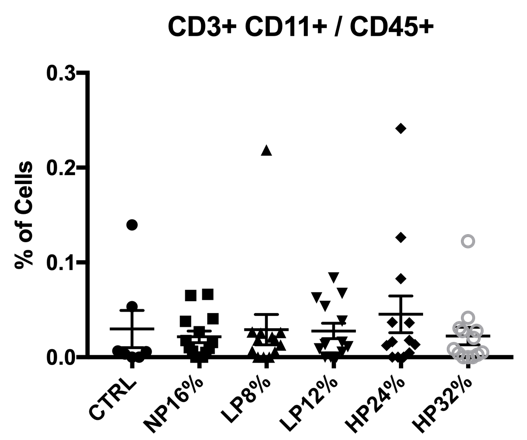

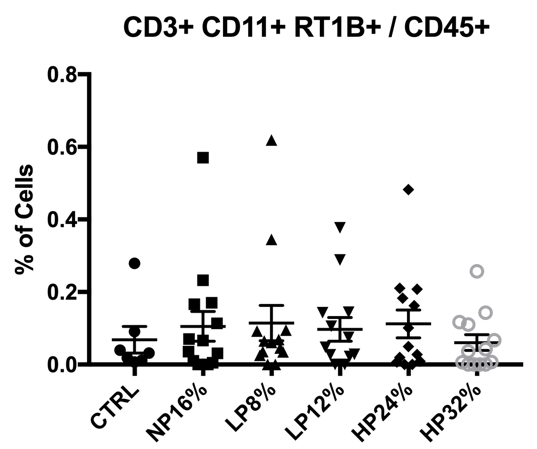


(g)

(h)

(i)


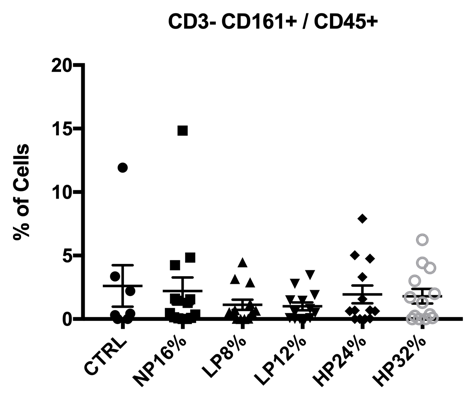


Number of events for total leukocytes and percentage of cells for other figures of immune cells identified in Peyer’s patches (*Table* S1). (a) CD45+ leukocyte cells; (b) CD4+ T-cells; (c) CD8+ T-cells; (d) CD4+ regulatory T-cells; (e) CD8+ regulatory T-cells; (f) CD3+ CD161+ Natural killer T-cells; (g)CD45R+RT1B+ B-cells; (h) CD3+ CD11+ monocytes; (i)CD3+ CD11+ RT1B+ dendritic cells; (j) CD3- CD161+ Natural killer cells. Scatterplots showing mean ± SEM. * is significantly different at p = 0.05.

(a) (b) (c) (d) (e) (f) (g) (h) (i) (j) CTRL, n=7; NP16%, n=14; LP8%, n=13; LP12%, n=13; HP24%, n=13; HP32%, n=13.

(j)

**Supplementary data** Immune exploration in tumour tissue


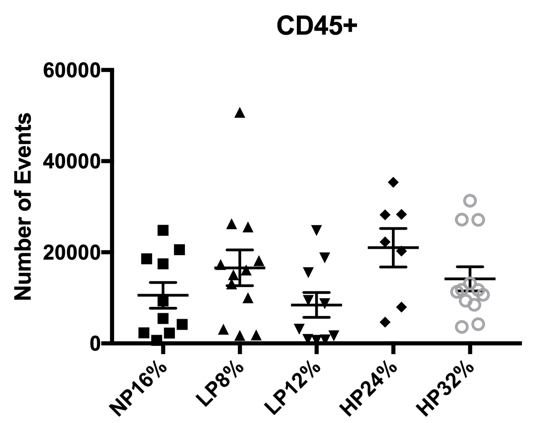

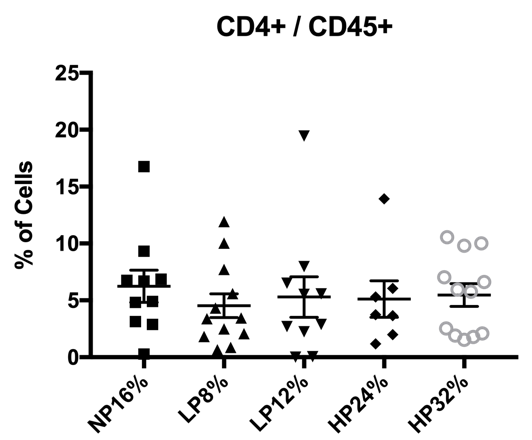

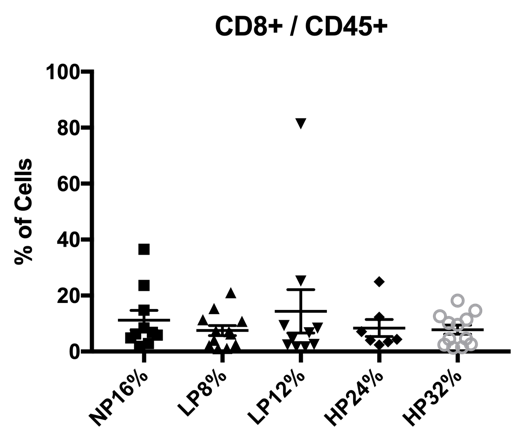


(d)

(e)

(c)

(b)

(a)


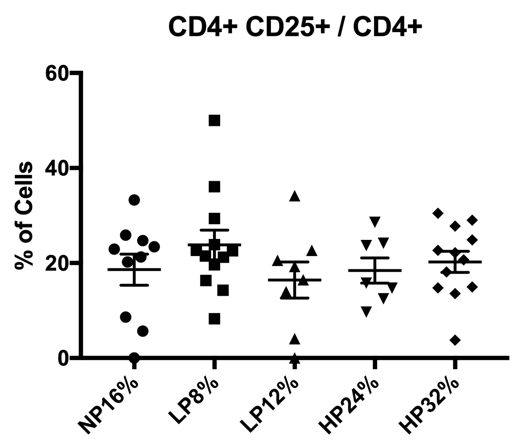

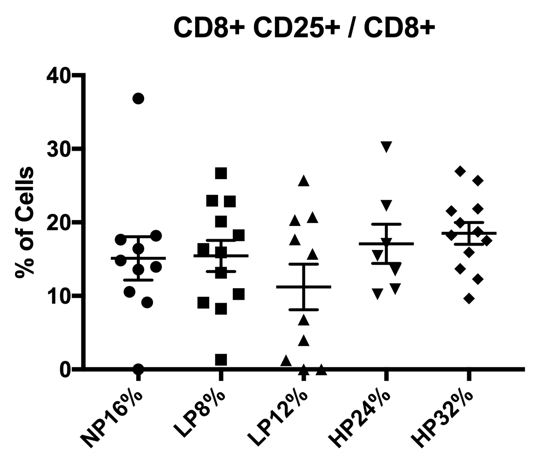

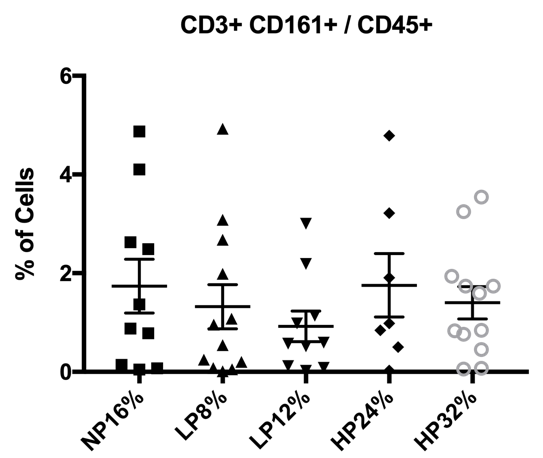


(f)


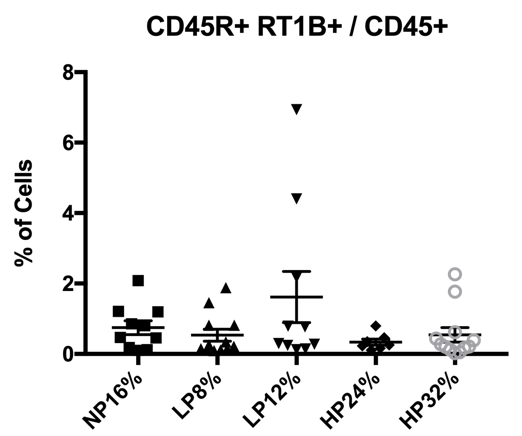

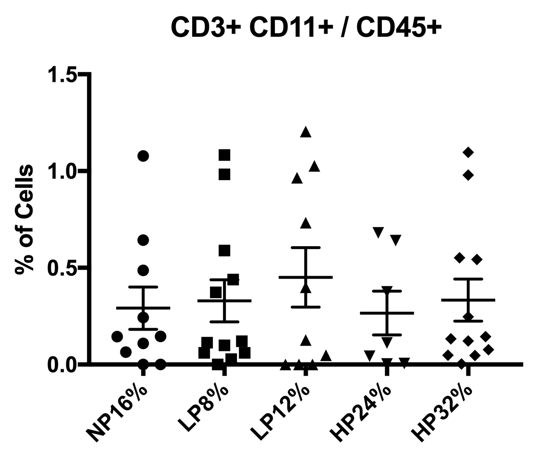

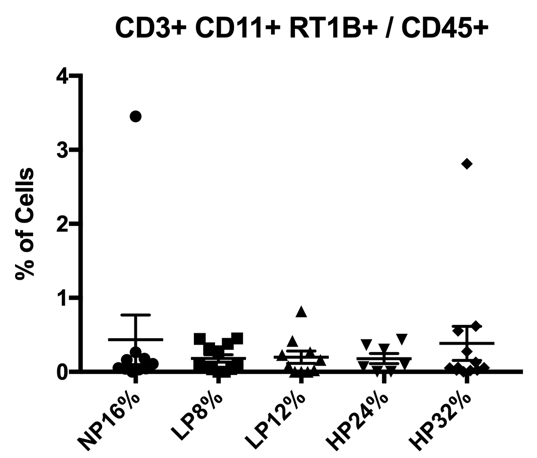


(j)

(i)

(h)

(g)


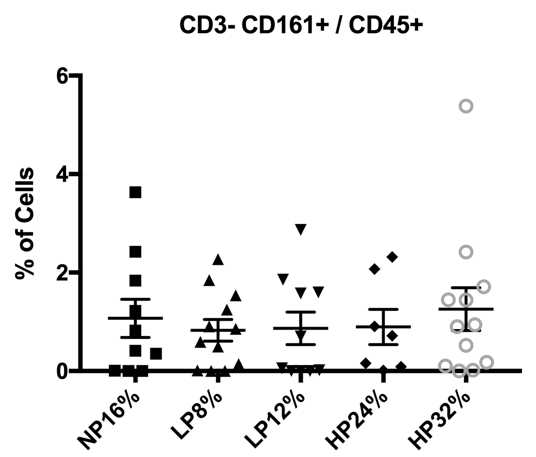


Number of events for total leukocytes and percentage of immune cells identified in tumour tissue (*Table* S1). (a) CD45+ leukocyte cells; (b) CD4+ T-cells; (c) CD8+ T-cells; (d) CD4+ regulatory T-cells; (e) CD8+ regulatory T-cells; (f) CD3+ CD161+ natural killer T-cells; (g)CD45R+RT1B+ B-cells; (h) CD3+ CD11+ monocytes; (i)CD3+ CD11+ RT1B+ dendritic cells; (j) CD3- CD161+ Natural killer cells. Scatterplots showing mean ± SEM. * is significantly different at p = 0.05.

(a) (b) (c) (d) (e) (f) (g) (h) (i) (j) NP16%, n=10; LP8%, n=12; LP12%, n=10; HP24%, n=7; HP32%, n=12.
