# Supplementary material for: Protein intake in cancer: Does it improve nutritional status and/or modify tumour response to chemotherapy?
Source: J Cachexia Sarcopenia Muscle. 2023 Sep 4;14(5):2003–15. doi: 10.1002/jcsm.13276 (PMC10570073; doi:10.1002/jcsm.13276)
Supplement: Supplementary file 4 — Table S1 Cell populations analyzed for immune exploration [file JCSM-14-2003-s001.docx]

**Table S1** Cell populations analyzed for immune exploration

|  | CD45 | CD3 | CD4 | CD8 | CD161 | CD25 | CD45R | CD11 | RT1B |
| --- | --- | --- | --- | --- | --- | --- | --- | --- | --- |
| T-cells | + | + | + | + |  | + |  |  |  |
| B-cells | + | - | - |  |  |  | + | - | + |
| NK cells | + | - |  |  | + |  |  |  |  |
| NKT cells | + | + |  |  | + |  |  |  |  |
| Monocytes and dendritic cells | + | - | + |  |  |  | - | + | - |
| Monocytes | + | + | - |  |  |  | - | + | - |
| Dendritic cells | + | + | - |  |  |  | - | + | + |
